# Tunable AMPA receptor function via recurrent evolution of heterotetramers

**DOI:** 10.1085/jgp.202614039

**Published:** 2026-08-27

**Authors:** Yuhong Wang, Alexander Edwards, Jelena Baranovic, Timothy Lynagh

**Affiliations:** 1 Michael Sars Centre, University of Bergen, Bergen, Norway; 2 School of Biological Sciences, University of Edinburgh, Edinburgh, UK

## Abstract

Most ionotropic neurotransmitter receptors are hetero-oligomers of three to five homologous subunits assembled into membrane-spanning ion channels. Mammalian AMPA-type glutamate receptors (AMPARs), which emerged in animals with centralized nervous systems and now mediate most of the excitatory synaptic signaling in our brain, readily assemble as homotetramers, yet often occur as heterotetramers of two to three different AMPAR subunits. Here, we looked for unifying functional properties of the AMPAR family using molecular phylogenetics, together with electrophysiological and pharmacological characterization of heterologously expressed AMPARs from the three major lineages within bilaterian animals. Our results suggest that the hallmark of AMPAR evolution is selective activation by glutamate, fast kinetics, and the duplication of AMPAR genes independently in new animal lineages, such that most major bilaterian groups have relatively unique complements of AMPAR genes. Moreover, in diverse bilaterians, these novel AMPAR subunits have (1) come to rely on each other for functional expression, resulting in obligate heterotetrameric AMPARs, and (2) sub-functionalized, with various subunits contributing differently to channel activation or ion permeation. Remarkably, this evolution appears to have independently converged on a complement of calcium-sensitive and calcium-insensitive AMPARs in mammals and worms via biophysically similar but genetically different mechanisms.

## Teaser

AMPA receptors are glutamate receptors specific to bilaterian animals with complex nervous systems. Wang et al. investigated the biophysical function, pharmacology, and kinetics of AMPA receptors from diverse bilaterians, uncovering convergent evolution on functional hallmarks, such as heteromerization and channel properties, throughout the AMPA receptor family.

## Introduction

Most excitatory synaptic signals between neurons in the mammalian brain are mediated by postsynaptic ionotropic glutamate receptors (iGluRs). IGluRs are tetramers in which four homologous subunits each have an extracellular ligand-binding domain (LBD) and together form a central cation-permeable channel. Long-standing functional and pharmacological data divide mammalian iGluRs into N-methyl-D-aspartate–sensitive iGluRs (NMDARs), α-amino-3-hydroxy-5-methyl-4-isoxazolepropionate–sensitive iGluRs (AMPARs), kainate–sensitive iGluRs (KRs), and Δ-type iGluRs (Hansen et al., 2021). These are reflected in more recent molecular phylogenies that show four major branches of animal iGluR genes: λ, NMDA, ε, and AKDF, with the latter comprising AMPAR, KR, Δ, and several related branches of genes not found in mammals (Ramos-Vicente et al., 2018; Moroz et al., 2021; Stroebel and Paoletti, 2021; Singh et al., 2025).

AMPARs are present at most excitatory synapses where their rapid kinetics shape the more exquisitely timed synaptic signaling in mammal brain (Yang et al., 2011; Tasic et al., 2018). AMPAR channels open within a millisecond and deactivate within 1–5 ms, which is ∼100-fold faster than NMDARs (Hestrin et al., 1990; Clements et al., 1992). And although open AMPAR channels can close within a few milliseconds with glutamate still bound to their LBDs in a process called desensitization, recovery from desensitization is also fast, some 100-fold faster than in closely related KRs (Otis et al., 1996; Carbone and Plested, 2012). Each mammalian AMPAR gene, *GRIA1*–*GRIA4*, encodes a subunit, GluA1–GluA4, that readily assembles into a glutamate-activated homotetramer when heterologously expressed (Keinänen et al., 1990; Sobolevsky et al., 2009; Hansen et al., 2021). This is in contrast to NMDARs, mostly obligate heteromers of glycine-binding GluN1 subunits and glutamate-binding GluN2 subunits, and heteromeric KRs, and instead reflects other KRs, numerous Δ iGluRs, and invertebrate AKDF and ε iGluRs that form functional homotetramers (Rosano et al., 2024; Seljeset et al., 2024; Gangwar et al., 2025; Singh et al., 2025; Wang et al., 2025).

Most native AMPARs in mammals, however, seem to be heterotetramers of various subunit combinations, with GluA1 and/or GluA3 subunits adopting the “A” and “C” positions, and GluA2 or GluA4 subunits adopting the “B” and “D” positions (Wenthold et al., 1996; Lu et al., 2009; Herguedas et al., 2019; Zhao et al., 2019; Yu et al., 2021; Fang et al., 2025; Scrutton et al., 2026). This has substantial functional consequences during channel gating, as all AMPAR structures to date indicate that although all four LBDs bind glutamate, glutamate-bound LBDs pull open the channel-forming helices of subunits B and D more so than subunits A and C (Chen et al., 2017; Twomey et al., 2017). Furthermore, the presence of two GluA2 subunits endows AMPARs with low Ca^2+^ permeability, which is further modulated by auxiliary subunits in most principal neurons in the mammalian brain (Sommer et al., 1991; Geiger et al., 1995; Miguez-Cabello et al., 2025). This is because GluA2 mRNA is selectively edited after transcription to encode a positively charged arginine residue in the AMPAR channel pore, practically abolishing Ca^2+^ conductance and decreasing but sparing Na^+^ and K^+^ conductance, both in simple GluA2 homotetramers and in, e.g., GluA1/GluA2 heterotetramers (Swanson et al., 1997; Heiser et al., 2025). Ca^2+^ conduction, channel gating, and cell surface expression can also be increased or decreased by alternative splicing of GluA subunits and by auxiliary proteins that associate with AMPARs (Schwenk et al., 2019; Cull-Candy and Farrant, 2021; Miguez-Cabello et al., 2025; Bowie, 2026). Thus, biophysical properties and physiological function of AMPARs depend on the presence of other proteins and on posttranscriptional and posttranslational modifications, in addition to evolutionary divergence of GluA genes themselves.

Molecular phylogenetic studies suggest that an AMPAR/KR-like ancestral gene emerged from duplications of more primitive AKDF genes (Ramos-Vicente et al., 2018; Moroz et al., 2021; Stroebel and Paoletti, 2021). Subsequently, the first AMPAR gene probably emerged from the duplication of an AMPAR/KAR-like gene early in Bilateria—the lineage of animals including worms, flies, and mammals, generally possessing a central brain and nerve cord(s)—after Bilateria diverged from Cnidaria, animals with simpler nervous systems (Bosch et al., 2017; Ramos-Vicente et al., 2018). This raises the possibility that AMPAR function is linked to the evolution of a sophisticated nervous system (Wang et al., 2008; Ramos-Vicente et al., 2018; Ramos-Vicente and Bayes, 2020; Bowie, 2026). Assessing this possibility requires the study of AMPARs from diverse bilaterians, especially since AMPAR genes may have diversified independently in different bilaterian animals (Ramos-Vicente et al., 2018).

Bilateria comprises three major lineages: Deuterostomia, including vertebrates and their close relatives; Protostomia, made up of spiralians, e.g., snails and flatworms, and ecdysozoans, e.g., insects and roundworms; and Xenacoelomorpha, a group of relatively simple worms. Studies of the protostomes *Caenorhabditis elegans* and *Aplysia californica* show that AMPARs are in postsynaptic membranes and important for behavior (Rongo et al., 1998; Li et al., 2009; Hoerndli et al., 2013). Heterologous expression of AMPARs from the invertebrate deuterostome *Ciona intestinalis* and the ecdysozoan protostomes *C. elegans*, *Drosophila melanogaster*, and *Apis mellifera* reveal glutamate-gated homotetramers, although in some cases only with co-expression of auxiliary proteins, and deactivation kinetics have not been assessed (Ultsch et al., 1992; Walker et al., 2006; Li et al., 2016; Hirai et al., 2017; Brunello et al., 2022). Perhaps poor heterologous expression is behind the fact that AMPARs from spiralian protostomes have not been studied in detail, although spiralians offer slower-evolving models of nervous system evolution and have on average 4× more AMPAR genes than ecdysozoans (Simakov et al., 2013; Northcutt et al., 2016; Ramos-Vicente et al., 2018). Moreover, Xenacoelomorpha, which likely branched off before the other two Bilaterian lineages and are thus crucial to dissecting putative ancestral Bilaterian features (Cannon et al., 2016; Martín-Durán et al., 2018; Liu et al., 2024), are lacking from all experimental and most phylogenetic studies.

The potential link between AMPARs and bilaterian evolution, and the molecular basis of AMPAR function, would both benefit from combined phylogenetic and functional studies of the AMPAR family, including oligomerization, pharmacology, ion conduction, and channel kinetics across diverse bilaterians. We therefore traced the evolutionary history of AMPARs and asked if AMPARs from each of the major bilaterian lineages show recognizable biophysical and pharmacological properties that might set them apart from other subfamilies. To do this, we used molecular phylogenetics and a comprehensive experimental comparison of AMPA receptors from a deuterostome, a protostome, and a xenacoelomorph. Our results confirm that new AMPAR subunits emerged independently in most bilaterian lineages and reveal that the resulting new subunits take on different functional roles in cognate receptor complexes, in some cases converging remarkably on key architectural and functional properties of AMPARs. This combined approach establishes convergent functional and architectural outcomes among diverse AMPARs despite their independent origins.

## Materials and methods

### Molecular phylogenetics

**Figure S1.**
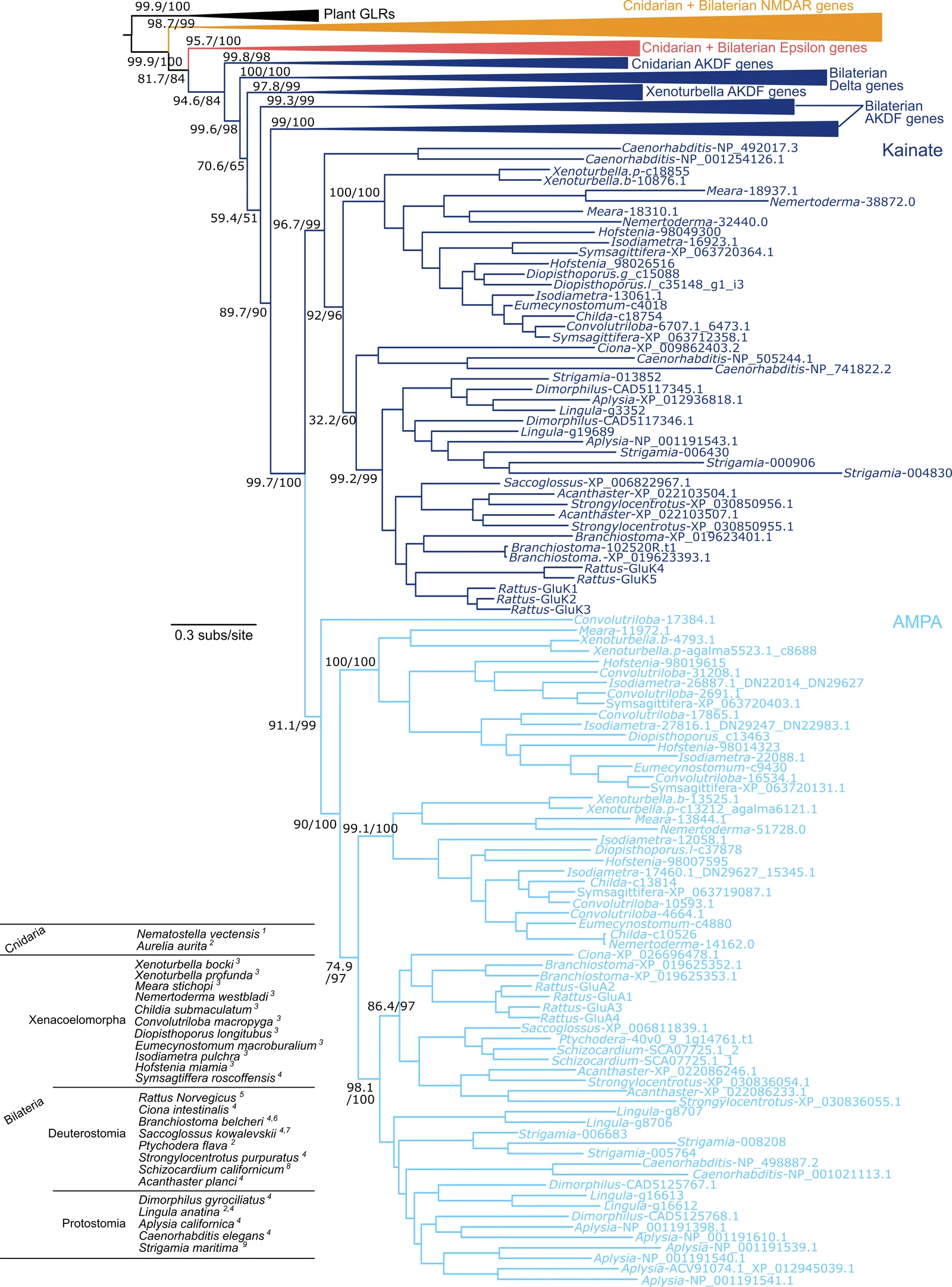
**Animal iGluR gene tree (manually trimmed alignment).** Maximum likelihood tree based on manually trimmed alignment of 386 cnidarian and bilaterian iGluRs and two plant GLRs as outgroup (see Materials and methods, Molecular phylogenetics). Branch support values are SH-aLRT and ultrafast bootstrap. Full alignment and tree available in Data S1 and S2. Inset table shows full species names; superscript numbers indicate databases used (websites listed in Materials and methods): 1, KEGG; 2, OIST; 3, published Xenacoelomorph datasets (Andrikou et al., 2019); 4, NCBI/GenBank; 5, UniProt; 6, LanceletDB; 7, *Saccoglossus* genome v3; 8, published *Schizocardium* datasets (Lin et al., 2024); 9, ENSEMBL Metazoa.

**Figure S2.**
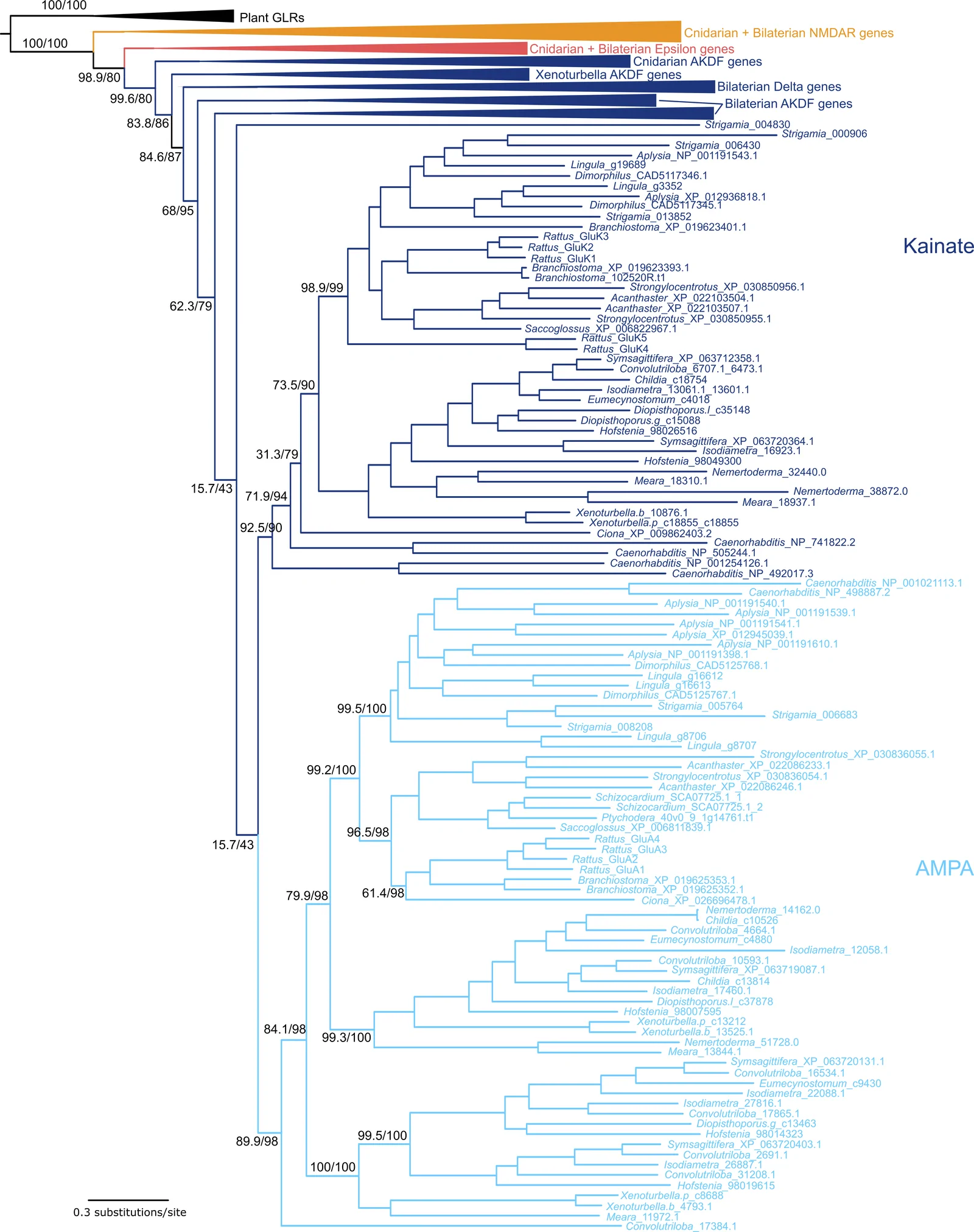
**Animal iGluR gene tree (untrimmed alignment).** Maximum likelihood tree based on untrimmed alignment (Data S3 and S4), including gaps and variable regions, of the same 388 iGluR sequences as Fig. S1 (see Materials and methods, Molecular phylogenetics). Branch support values are SH-aLRT and ultrafast bootstrap.

iGluR amino acid sequences were extracted via BlastP (Altschul et al., 1997) or tBlastN from diverse cnidarians and bilaterians using NCBI/GenBank (https://blast.ncbi.nlm.nih.gov/Blast.cgi?PAGE=Proteins), UniProt (https://www.uniprot.org/), ENSEMBL (http://metazoa.ensembl.org/index.html), KEGG (https://www.genome.jp/tools/blast/), OIST Marine Genomics Unit (https://marinegenomics.oist.jp/), LanceletDB (You et al., 2019), previously published xenacoelomorph (Andrikou et al., 2019; Gehrke et al., 2019) and hemichordate (Simakov et al., 2015; Lin et al., 2024) datasets, and updated (v3) *Saccoglossus kowalevskii* genome (http://bioinfo.szn.it/genoma-repo/Skowalewski/OIST/Saccoglossus_kowalevskii_v3.fasta.tbz2). Species names and their database sources are listed in Fig. S1. Sequences were aligned using MAFFT v7.490 (Katoh et al., 2002) within Geneious Prime software (Dotmatrix). Sequences that were >95% identical to others or aligned poorly due to obvious repeated sequences within genes were removed. All remaining sequences were realigned and checked for the presence of the conserved “SYTANLAA” (or similar) amino acid motif in the putative channel-forming M3 helix. We also included two plant glutamate receptor-like sequences from *Arabidopsis thaliana* as an outgroup. The sequence alignment contained 388 sequences and 6,689 columns (including gaps). After manually removing gaps and highly variable regions according to the consensus sequence, a final alignment with 672 columns was used to generate a maximum likelihood tree with IQ-TREE (Minh et al., 2020) using an LG + I + G4 substitution model (Minh et al., 2021) and SH-aLRT (Guindon et al., 2010) and ultrafast bootstrap (Hoang et al., 2018) for branch support (Fig. S1). The tree based on the trimmed alignment was used in the main figures (alignment and tree available as Data S1 and S2), but a tree based on the untrimmed alignment is shown in Fig. S2 (and available as Data S3 and S4).

The transmembrane AMPAR auxiliary protein (TARP) gene tree was generated via the same methods, however, in focusing specifically on the TARP subfamily of the overarching TARP-CACNG-GSG1 family, we ensured we retrieved TARP-like sequences from all animals of interest by blasting with rat TARP-γ2, GSG1L, and CACNG1 (Fig. S1 for species and source databases), by running preliminary trees with our retrieved sequences and selecting for genes in TARP and closely related CACNG and GSG1L branches; there are more genes in the overarching family but outside of these branches, e.g., EMP1 and PMP22, but we excluded most of them and used only human EMP1 and PMP22 as outgroups, based on previous work on this family (Ramos-Vicente and Bayes, 2020). The sequence alignment contained 65 sequences and 1,056 columns (including gaps). After manually removing gaps and highly variable regions according to the consensus sequence, a final alignment with 181 columns was used to generate a maximum likelihood tree with IQ-TREE (Minh et al., 2020) using an LG + F + I + G4 substitution model (Minh et al., 2021) and SH-aLRT (Guindon et al., 2010) and ultrafast bootstrap (Hoang et al., 2018) for branch support (see Fig. S6). The tree based on the trimmed alignment was used in the main figures (alignment and tree available as Data S5 and S6), but a tree based on the untrimmed alignment is shown in Fig. S7 (and available as Data S7 and S8).

### Plasmids and molecular biology

**Figure S3.**
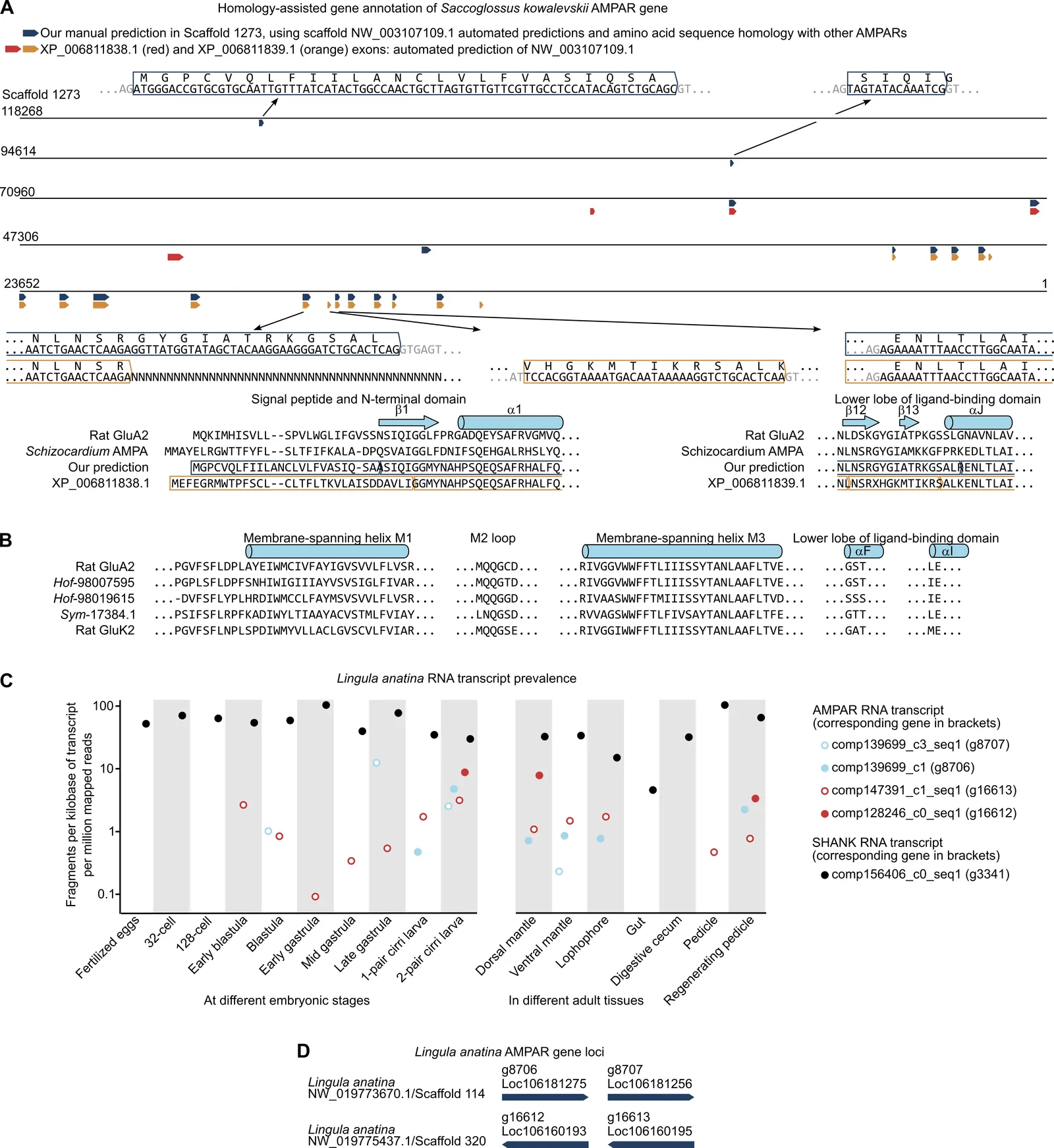
**AMPAR gene expression, gene loci, and gene annotation AMPAR gene expression, gene loci, and gene annotation. (A)** Scaffold 1273 from *S. kowalevskii* genome version 3 http://bioinfo.szn.it/genoma-repo/Skowalewski/OIST/Saccoglossus_kowalevskii_v3.fasta.tbz2 (similar to genome version 2/GCF_000003605.2, scaffold 2021/NW_003107109.1 at NCBI GenBank [Simakov et al., 2015]), base pair numbering at left of black lines, with our manually annotated exons shown as blue arrows. Also shown are previous, automatically annotated exons (red and orange arrows) corresponding to NCBI entries XP_006811838.1 and XP_006811839.1 from NW_003107109.1. Translations above and below highlight selected differences between manual and automatic predictions; amino acid sequence alignments (below) of these segments illustrate how we also used homology with fellow hemichordate AMPAR (*S. californicum*) and rat AMPAR to identify likely exons (secondary structure from published *Rat*-GluA2 PDB:3KG2 [Sobolevsky et al., 2009]). Updated NCBI/GenBank entry XP_085353805.1 is 97% identical to our prediction (99% excluding signal peptide). **(B)** Amino acid sequence alignment of key pore-forming and ligand-binding segments of *S. roscoffensis* AMPAR-like 17384.1 and other genes. **(C)** RNA transcript prevalence data from a previous *L. anatina* genomic study (Luo et al., 2018). SHANK is a postsynaptic protein included to show a gene with higher expression than AMPA genes. **(D)** Adjacent location of *L. anatina* AMPAR genes in indicated scaffolds from genome GCF_001039355.2 (NCBI GenBank) (Luo et al., 2018).

*Rattus norvegicus* GluA1 (flip,Q isoform) and GluA2 (G and flip isoform in LBD, not Q/R edited in pore) in the pRK5 vector and without poly(A) tails were a gift from Dr. David MacLean (University of Rochester Medical Center, Rochester, NY, USA). Human TARP-γ2 (stargazin) in a modified pcDNA3.1(+) vector containing *Xenopus laevis* β-globin 5′ and-3′ UTRs and a poly(A) signal (Chua et al., 2020) was a gift from Stephan Pless (University of Copenhagen, Copenhagen, Denmark). All other novel cDNAs were commercially synthesized (GenScript) and subcloned into either a modified pSP64 vector containing *X. laevis* β-globin 5′ and-3′ UTRs and a poly(A) tail (Seljeset et al., 2024) or into pIRES2-EGFP (Clontech) for expression in HEK293 cells. All novel cDNAs were codon-optimized in silico at iCodon (Diez et al., 2022) for either *X. laevis* or human before synthesis, except for *S. kowalevskii* oocyte constructs. A *S. kowalevskii* GluA sequence was reanalyzed from updated genomic information after the phylogenetic work and used for expression; this is 85% identical to the combined NCBI/GenBank outdated entries XP_006811838.1 and XP_006811839.1 and 97% identical to updated entry XP_085353805.1 (Fig. S3 A). All synthetic DNA sequences are given in Tables S1, S2, S3, and S4. To predict signal peptides, we used SignalP 6.0 (Teufel et al., 2022).

Mutations were made via PCR on the full plasmids using partially overlapping primers and Phusion Hot Start II DNA Polymerase (Thermo Fisher Scientific) according to a published protocol (Xia et al., 2015). Protein-coding inserts were fully sequenced via Sanger sequencing (Genewiz).

For two-electrode voltage clamp, plasmids were linearized via restriction digest at sites downstream of the coding sequence (*Rat*-GluA1, *Rat*-GluA2, and human TARP-γ2) or downstream of the poly(A) segment (all others) and purified via DNA Clean & Concentrator-5 columns (Zymo Research). Capped RNA was transcribed for 2 h at 37°C with mMESSAGE mMACHINE kits (Thermo Fisher Scientific, T7 for pcDNA3.1(+), SP6 for modified pSP64), followed by DNAse treatment and purification with RNA Clean & Concentrator-5 (Zymo Research) or RNeasy (Qiagen) kits.

### Two-electrode voltage clamp

Defolliculated *X. laevis* stage V/VI oocytes (shipped weekly from Ecocyte Bioscience) were stored at 8°C in 50% (in water) Leibovitz’s L-15 medium (Gibco) including additional 0.25 mg/ml gentamicin, 1 mM L-glutamate, and 15 mM HEPES, pH 7.6, in glass Petri dishes or in 35 × 10-mm cell culture plastic dishes on a gentle shaker to prevent flattening. Oocytes were injected with 10 ng (rat AMPAR), 10 ng (*S. kowalevskii* AMPAR) and 1 ng (*S. kowalevskii* TARPs), 10 ng (*Lingula anatina* AMPAR), or 1 ng (*L. anatina* TARPs), 30 ng (*Hofstenia miamia*), or 3 ng (*H. miamia* TARPs) RNA in 40 nl water using a Nanoliter2010 injector and glass micropipettes (World Precision Instruments). Injected oocytes were stored in the same modified L-15 medium (above) at 18°C or 16°C until electrophysiological recordings.

1 to 2 days (rat receptors and *Lingula* receptors) or 3 to 4 days (*Saccoglossus* and *Hofstenia* receptors) after injection, each oocyte was placed in an RC-3Z bath (Warner Instruments) and continuously perfused with bath solution. For most experiments, bath solution contained (in mM) 96 NaCl, 2 KCl, 1.8 CaCl_2_, 1 MgCl_2_, and HEPES 5 mM, pH adjusted to 7.5 with NaOH. For I-V experiments without Ca^2+^, the bath solution instead contained 1.8 mM BaCl_2_. The bath solution was exchanged for one containing the ligand, as indicated by bars in the figures, with a VCS-8-pinch valve control perfusion system (Warner Instruments). Ligands were stored as 100–300 mM stocks in water or bath solution, pH-adjusted with NaOH (monosodium glutamate, monosodium aspartate, CNQX, cyclothiazide, DNQX, and NBQX), and dissolved in bath solution before use. CNQX, DNQX, and NBQX were purchased from Alomone Labs; all others from Merck. Oocytes were impaled with microelectrodes made of borosilicate micropipettes containing silver-chloride wire in 3 M KCl and clamped at −80 mV with an Oocyte Clamp OC-725D amplifier (Warner Instruments) and Axon Digidata 1550B digitizer (Molecular Devices). Current was sampled at 500 or 1,000 Hz and low-pass filtered at 100 or 200 Hz.

### Outside-out patch clamp

HEK293 cells (Sigma-Aldrich) were maintained in minimum essential medium (MEM, Gibco) supplemented with 10% (vol/vol) fetal bovine serum (FBS, Gibco), 2 mM L-glutamine (Gibco), and 50 U ml^−1^ penicillin-streptomycin (Gibco) at 37°C in a humidified 5% CO_2_ incubator and seeded at a density of 3 × 10^6^ onto 13-mm borosilicate glass coverslips (Avantor) pre-coated with poly-L-lysine (4.5 μg ml^−1^, Sigma-Aldrich) for transient transfections 24 h later with Lipofectamine 3000 (Sigma-Aldrich) and AMPAR-GFP and TARP plasmids (see ratios in Table S5). 48–96 h after transfection, coverslips were placed in an external recording solution containing (in mM) 150 NaCl, 50 HEPES, 1 MgCl_2_, and 1 CaCl_2_, adjusted to pH 7.3 (280 mOsm) on an inverted microscope (IX73; Olympus), and eGFP-positive cells were identified under a pE-300lite LED (CoolLED). As eGFP was common to all constructs, its presence indicated plasmid uptake but did not distinguish between uptake and/or expression of all intended constructs.

Patch-clamp electrodes were pulled from borosilicate glass capillaries (1.5 mm o.d., 1.17 mm i.d.; Harvard Apparatus) and fire-polished to yield a resistance of 3–8 MΩ. The internal pipette solution contained (in mM) 115 NaCl, 10 NaF, 5 Na_4_BAPTA, 0.5 CaCl_2_, 1 MgCl_2_, 5 HEPES, and 10 Na_2_ATP (pH adjusted to 7.3 with NaOH, 280–300 mOsm). Recordings were performed at 22–25°C from outside-out membrane patches using an Axopatch 200B amplifier (Molecular Devices). Signals were low-pass filtered at 5 kHz, digitized at 25 kHz with a Dendrite digitizer (Sutter Instruments). Currents were recorded at holding potentials of −60 mV or −40 mV.

Rapid solution exchange over excised patches was performed using a custom-built, four-barrel borosilicate glass application tool (CM Scientific) mounted on a piezoelectric translator (Physik Instrumente) (Plested and Poulsen, 2021). Solution exchange was achieved by piezo-driven lateral movement of the tool, producing 10–90% solution exchange times below 200 μs. The exchange rate was measured at the end of each experiment by rupturing the patch and rapidly exchanging the solutions over the open pipette tip.

### Data analysis

Two-electrode voltage clamp currents were additionally filtered (50 Hz, Bessel 8-pole) and measured in Clampfit (Molecular Devices). Data were plotted in Prism v9 (GraphPad). For column graphs, we have shown all data points and the mean. For concentration-response and I-V graphs, for clarity, we have shown only the mean ± SD, and then fitted four-parameter Hill equations (concentration-response) or lines (I-V) for display. When reporting EC_50_ values or percentage of modulation, values were calculated for each individual oocyte and then mean ± SD reported. EC_50_ values were calculated by variable slope nonlinear regression (Prism v9). For graphical presentation and statistical analysis of differences in EC_50_ values, we converted these to −log_10_(EC_50_) (pEC_50_) values that are normally distributed and compared with one-way ANOVA and Tukey’s multiple comparisons test in Prism v9. Patch-clamp currents from HEK293 cells were analyzed in SutterPatch software (v3.2.1, Sutter Instruments). Time constants for activation, desensitization, and deactivation were calculated from exponential fits with one component. In paired pulse experiments addressing recovery from desensitization, the extent of recovery was measured by calculating the ratio of the peak current evoked by the second pulse relative to that produced by the initial conditioning pulse. The time course of recovery was characterized by fitting the data with a Hodgkin–Huxley-type function (Carbone and Plested, 2012)

$$N=N_{t}+\left(1-N_{t}\right)\cdot {\left[1-\exp\left(-k_{rec}t\right)\right]}^{n},$$

where *N* is the fraction of receptors recovered at time *t*, *N*_*t*_ represents the fraction of receptors available immediately after the conditioning pulse, *k*_*rec*_ is the recovery rate constant, and *n* is the slope (Hodgkin and Huxley, 1952). During fitting, the maximal recovery was constrained to unity. Statistical analyses of rate constants were performed using two-tailed unpaired *t* tests.

### Online supplemental material

**Figure S4.**
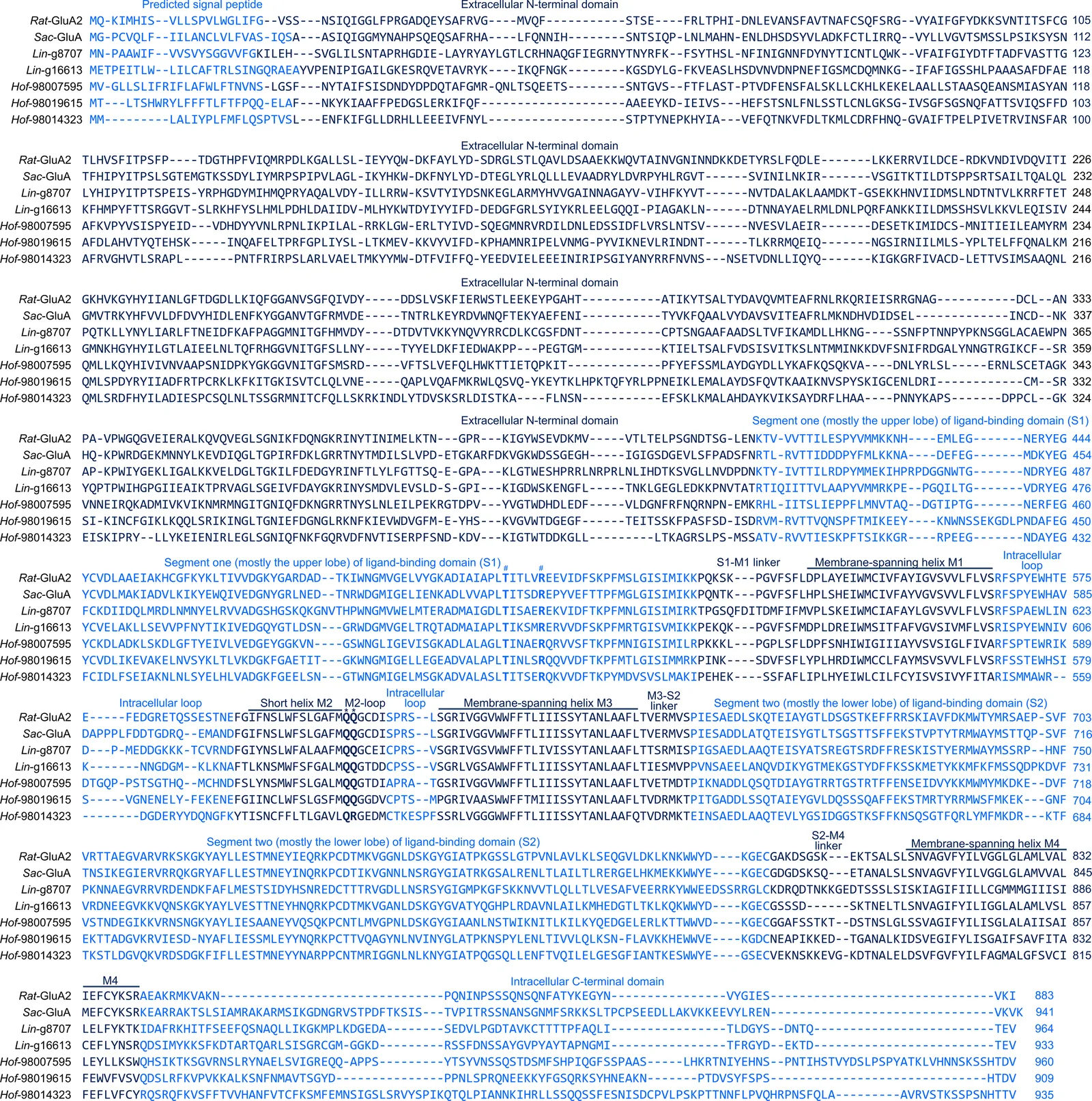
**Amino acid sequence alignment of AMPAR subunits studied here. **The seven subunits were aligned with MAFFT (Katoh et al., 2002). Except for rat GluA2, indicated signal peptides are predictions from SignalP 6.0 (Teufel et al., 2022), with SignalP 6.0 likelihoods (where 1 is maximum) of 0.9991 for *Sac*-GluA, 0.0633 for *Lin*-g8707, 0.9992 for *Lin*-g16613, 0.9048 for *Hof*-98007595, 0.0663 for *Hof*-98019615, and 0.0432 for *Hof*-98014323. Indicated structural/functional domains are inferred from 3D GluA2 structures PDB accession no. 5WEO (Twomey et al., 2017) and PDB accession no. 1FTJ (Armstrong and Gouaux, 2000). #, amino acid residues mutated in Fig. 3 B. *, Q1 and Q2 (or R2) residues discussed in Fig. 4 and Fig. 5 E.

**Figure S5.**
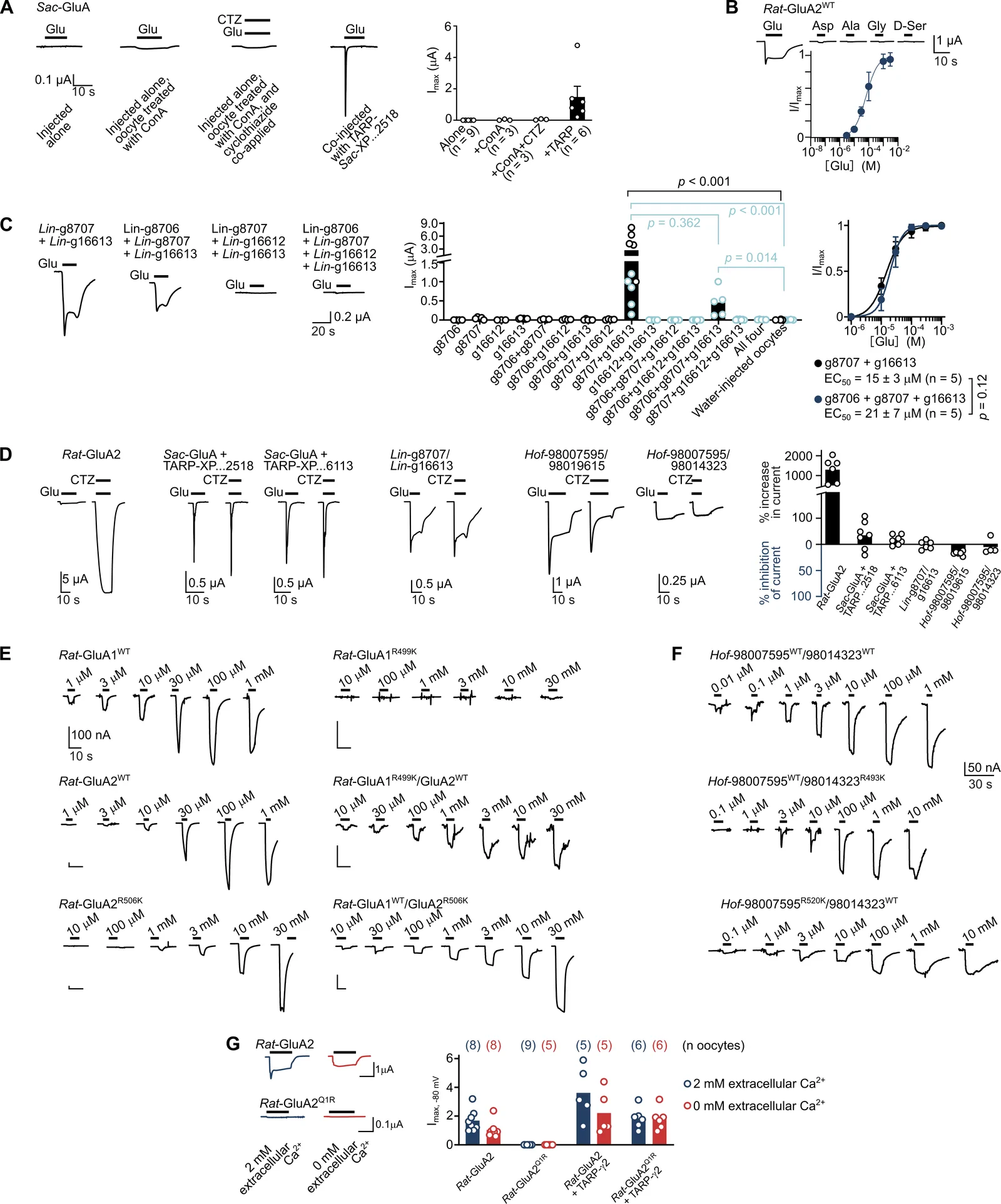
**Functional characterization of diverse AMPARs. (A)** Example recordings and mean (columns) current amplitude of responses to glutamate (1 mM) in oocytes injected with *S. kowalevskii* GluA RNA alone; alone and then with concanavalin A (ConA) treatment; and when co-injected with *Saccoglossus* TARP-XP_006822518.1. **(B)** Example recording of glutamate selectivity of *Rat*-GluA2 and mean ± SD (n = 4) normalized current amplitude in response to increasing concentrations of glutamate at *Rat*-GluA2. **(C)** Left, example recordings; and middle, mean (column) and individual oocyte current responses to 1 mM glutamate. Black data points from one batch of oocytes/experiments; cyan from another. Data were compared within batches, with one-way ANOVA and Tukey’s multiple comparisons test; selected P values shown; all other columns not significantly different from water-injected oocytes from that batch. Right, mean ± SD (n = 5) current (normalized to maximum) in response to increasing glutamate concentrations for indicated cRNA combinations. P value for unpaired *t* test of EC_50_s. **(D)** Left/middle, example recordings showing the effects of cyclothiazide (CTZ, 100 µM) on glutamate (1 mM) -gated currents. Right, mean (column) and individual data (dots) for CTZ-induced percent increase in glutamate-gated current or percentage of inhibition of glutamate-gated current. **(E)** Example recordings of glutamate-gated currents through indicated rat AMPARs, all scale bars as indicated top-left. **(F)** Example recordings of indicated *H. miamia* AMPARs, all scaled according to bars at right. **(G)** Example, mean (column), and individual glutamate-gated current amplitudes (−80 mV) in oocytes expressing indicated WT and mutant Q1R *Rat*-GluA2 with or without human TARP-γ2.

**Figure S6.**
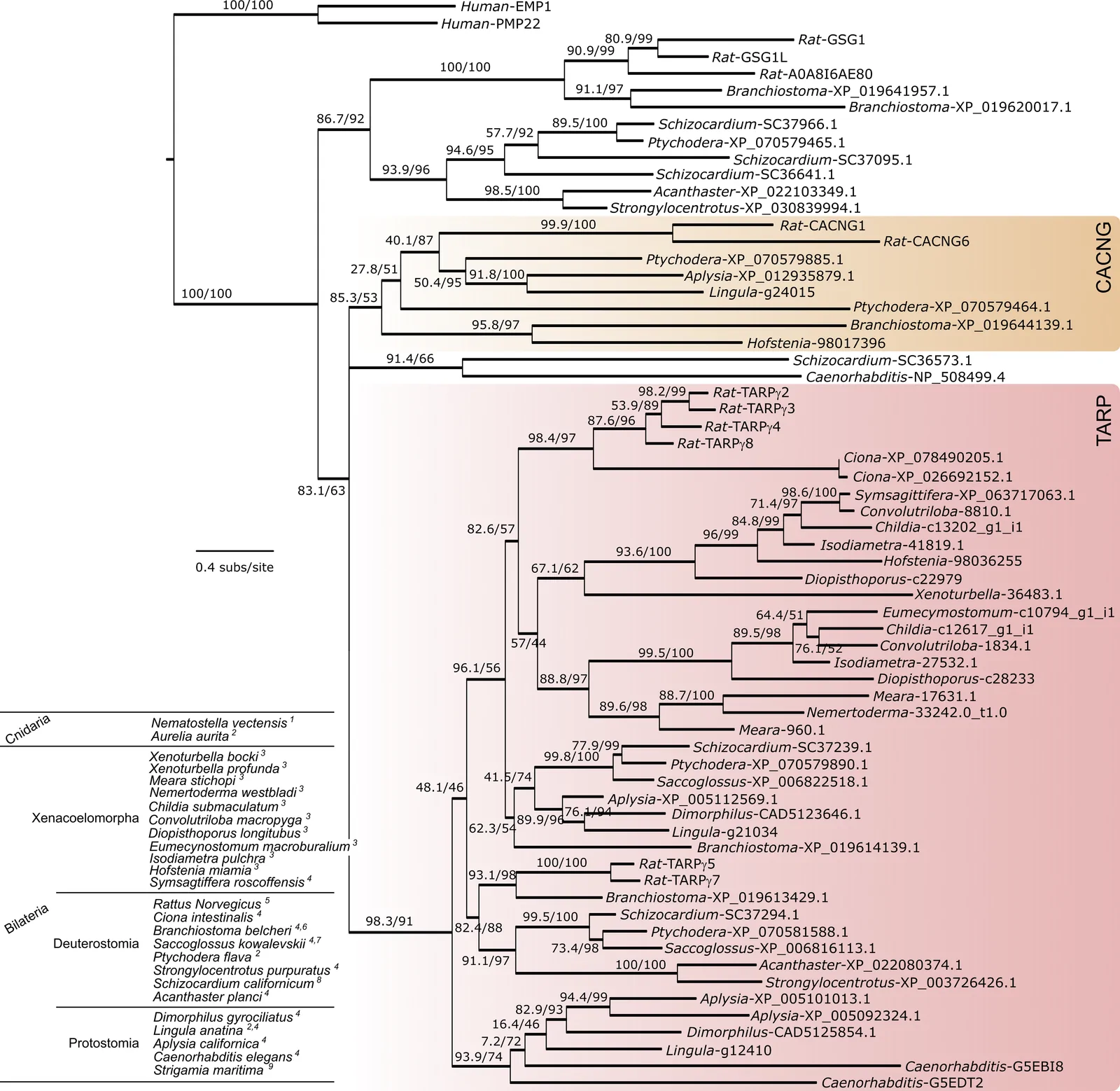
**TARP-CACNG-GSG1 gene tree (manually trimmed alignment).** Maximum likelihood tree based on manually trimmed alignment of 65 TARP-like sequences (see Materials and methods, Molecular phylogenetics). Alignment and tree downloadable as Data S5 and S6. Inset table shows full species names; superscript numbers indicate databases searched (websites listed in Materials and methods): 1, KEGG; 2, OIST; 3, published Xenacoelomorph datasets (Andrikou et al., 2019); 4, NCBI/GenBank; 5, UniProt; 6, LanceletDB; 7, *Saccoglossus* genome v3; 8, published *Schizocardium* datasets (Lin et al., 2024); 9, ENSEMBL Metazoa. Human EMP1 and PMP22 from UniProt. There are more genes in the overarching family outside of these branches e.g., EMP1 and PMP22, but we excluded most of them and used only human EMP1 and PMP22 as outgroups, based on previous work on this family (Ramos-Vicente and Bayes, 2020).

**Figure S7.**
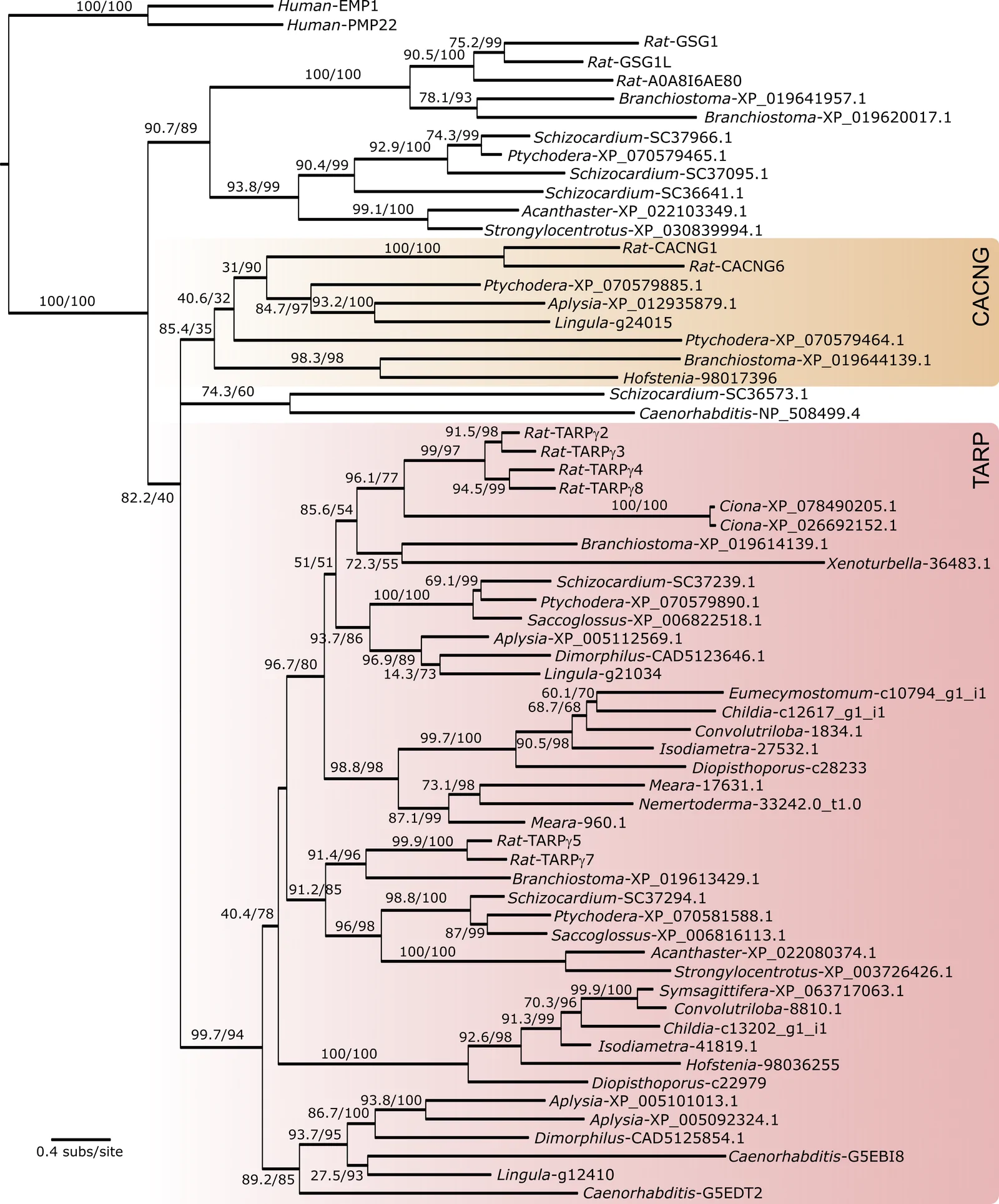
**TARP-CACNG-GSG1 gene tree (untrimmed alignment).** Maximum likelihood tree based on untrimmed alignment (Data S7 and S8), including gaps and variable regions, of the same 65 TARP-like sequences as Fig. S6 (see Materials and methods, Molecular phylogenetics).

**Figure S8.**
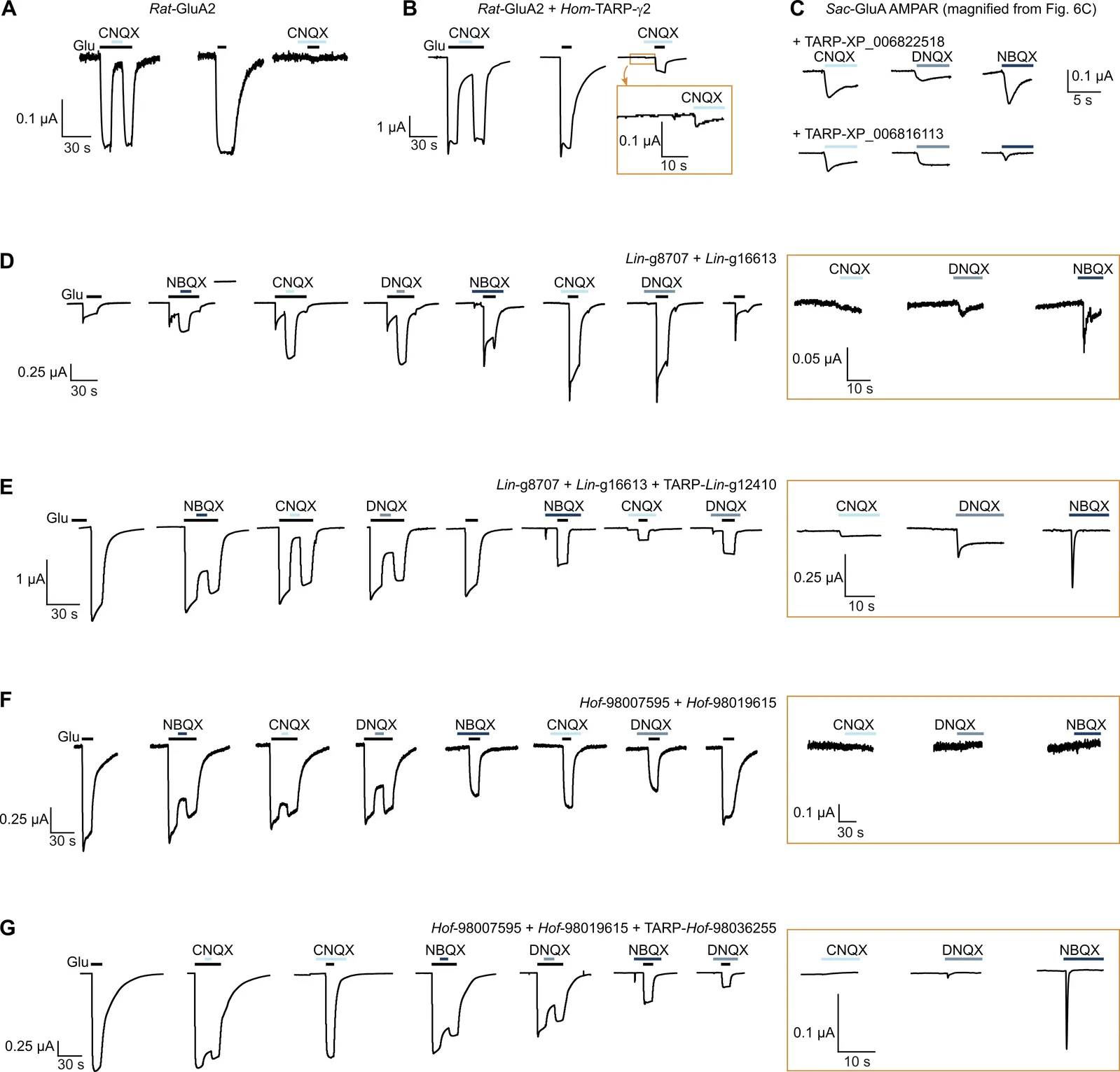
**Effects of quinoxalinediones on diverse AMPARs. (A–** **G** **)** Example recordings from oocytes expressing indicated AMPARs and TARPs. Quinoxalinediones CNQX, DNQX, and NBQX (each at 100 µM) were used in two protocols: glutamate (1 mM) was pre-applied, and during glutamate application the quinoxalinedione was co-applied (e.g., A, left); or quinoxalinedione was pre-applied, and then glutamate was co-applied (e.g., A, right). Note how the quinoxalinedione-mediated decreases in glutamate-gated current amplitude (A–F) or quinoxalinedione-mediated increases in glutamate-gated current amplitude (C) are similar in either protocol. Insets in B–F show magnified views of the same recordings to illustrate currents gated by quinoxalinediones in some receptors (note different scale bars).

Fig. S1 shows the large animal iGluR gene tree giving rise to the AMPAR branch on which we focused. Fig. S2 shows a similar tree but derived from an untrimmed amino acid sequence alignment. Fig. S3 details gene expression, gene loci, and gene annotation of certain AMPARs utilized in this study. Fig. S4 is an amino acid sequence alignment of AMPAR subunits characterized here, including the familiar *Rat*-GluA2 subunit. Fig. S5 shows example recordings behind the main findings in Figs. 2 and 3. Fig. S6 is a fuller gene tree of TARP-like proteins used for the work in Fig. 5. Fig. S7 is a similar TARP tree but derived from an untrimmed alignment. Fig. S8 shows example recordings behind the main findings in Fig. 6. Table S1 details synthetic AMPAR genes used for expression in *X. laevis* oocytes. Table S2 details synthetic TARP genes used for expression in *X. laevis* oocytes. Table S3 details synthetic AMPAR genes used for expression in HEK293 cells. Table S4 details synthetic TARP genes used for expression in HEK293 cells. Table S5 is a summary of outside-out patches. Data S1, S2, S3, S4, S5, S6, S7, and S8 include amino acid sequence alignments (Data S1, S3, S5, and S7) and gene trees (Data S2, S4, S6, and S8) for iGluRs (Data S1 manually trimmed iGluR amino acid sequence alignment [FASTA format] and S2, iGluR gene tree [NEXUS format] based on trimmed alignment; Data S3 untrimmed iGluR amino acid sequence alignment [FASTA format] and S4, iGluR gene tree [NEXUS format] based on untrimmed alignment) and for TARPs (Data S5 manually trimmed TARP amino acid sequence alignment and S6, TARP gene tree based on trimmed alignment; Data S7 untrimmed TARP amino acid sequence alignment and S8, TARP gene tree based on untrimmed alignment).

## Results

### Recurrent AMPAR gene duplications in diverse bilaterian lineages

**Figure 1.**
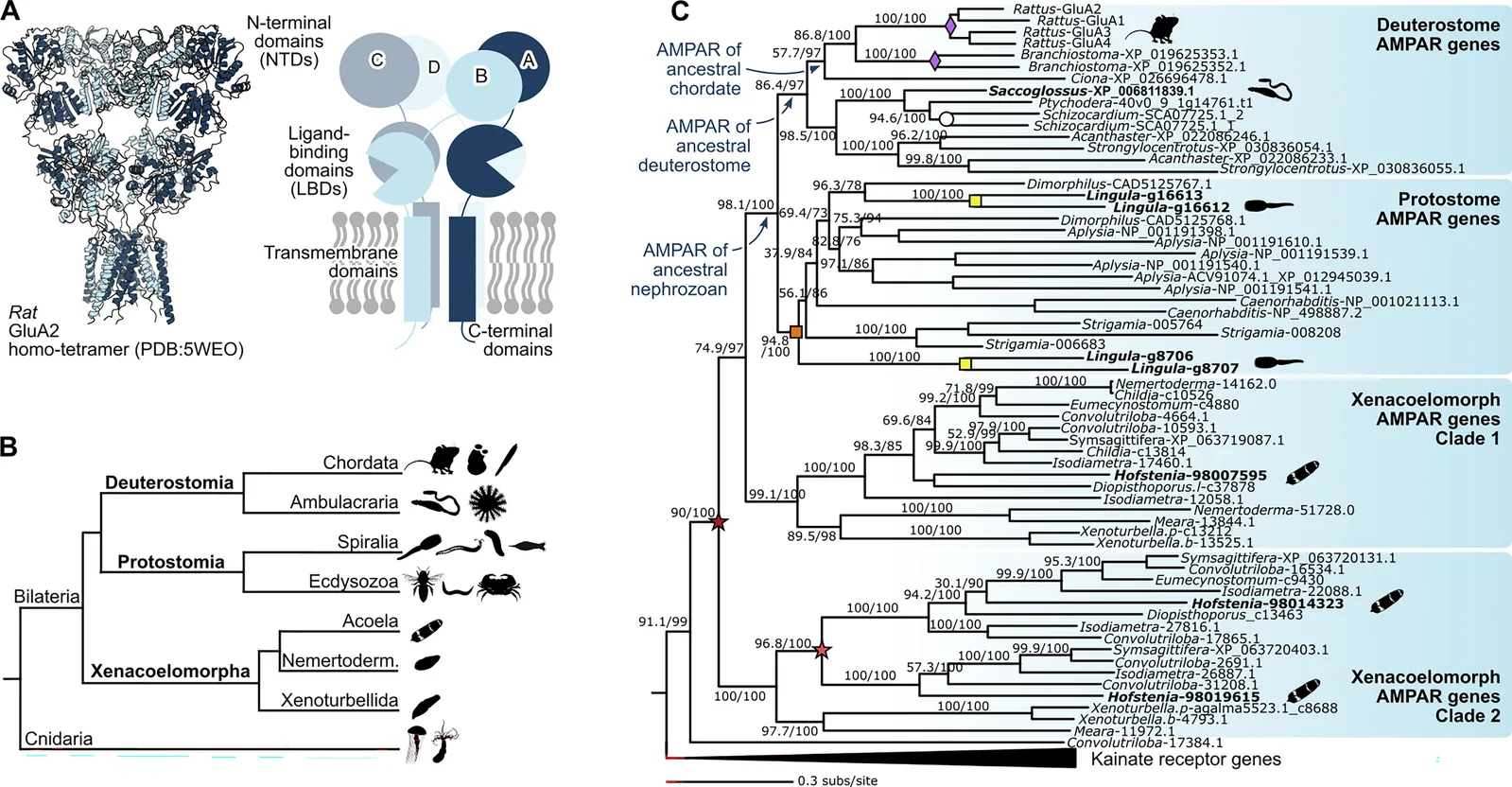
**Lineage-specific gene duplications dominate AMPAR evolution. (A)** Left, rat AMPAR structure from PDB accession no. 5WEO, and right, cartoon indicating subunits A through D and domains within subunits. **(B)** Animal tree (branches not to scale) showing relationships of Bilateria. Three major lineages in bold. **(C)** AMPAR branch from iGluR gene tree (full tree in Fig. S1, branch support values are SH-aLRT and ultrafast bootstrap). Blue labels/arrows: selected putative ancestral genes arising from speciation. Circles, diamonds, squares, and stars: gene duplications discussed in the main text. Bold gene names: studied in subsequent figures.

Previous phylogenetic studies agree that AMPARs emerged in Bilateria from a duplication of an AMPAR/KR-like ancestor, after Bilateria split from Cnidaria (Fig. 1, A and B; Ramos-Vicente et al., 2018; Moroz et al., 2021; Stroebel and Paoletti, 2021; Singh et al., 2025). To closely investigate AMPAR evolution, we generated an iGluR gene tree based on amino acid sequences from cnidarians and bilaterians, but in contrast to most previous studies, we incorporated recent data from Xenacoelomorpha, the earliest branching bilaterian lineage, and thus key to resolving the evolution of bilaterian genes (Cannon et al., 2016; Heger et al., 2020). Our analysis returns a well-supported AMPAR branch, with SH-aLRT and ultrafast bootstrap supports of 91.1 and 99 (Fig. 1 C), and with similar topology when based on alignments with or without variable regions (Figs. S1 and S2). Our phylogeny confirms that AMPAR genes are absent from cnidarians and shows that AMPAR genes are indeed present in each major lineage of bilaterians—Deuterostomia, Protostomia, and Xenacoelomorpha (Fig. 1 C). This indicates that AMPARs evolved extremely early in the bilaterian lineage, shortly after the Bilateria/Cnidaria split.

Our phylogeny also shows that most animals considered have several AMPAR genes that seem specific to that animal lineage. This could be the result of numerous AMPAR genes in an ancestral bilaterian, which must have resulted from numerous closely timed duplications, followed by independent losses of many of those genes together with selective retention of just a few specific AMPAR genes in most lineages. Alternatively, it could be that the ancestral bilaterian had a relatively small number of AMPAR genes, and well after the divergence of the three major bilaterian lineages from each other, AMPAR genes have undergone several duplications numerous times independently. We favor the second alternative as more parsimonious because the first involves more numerous duplications and losses in total and curious patterns of shared loss but selective retention in different animals (Hahn, 2007). Another study that explored numerous protostome AMPAR and KR genes also favored the scenario of independent gene duplication for AMPARs (Ramos-Vicente et al., 2018).

One example of lineage-specific gene duplications regards the four classical AMPAR subunits, GluA1–GluA4. These are unique to vertebrates, suggesting GluA1–GluA4 resulted from duplications occurring after vertebrates diverged from other chordates, much like the two AMPAR genes in the cephalochordate *Branchiostoma belcheri* (Fig. 1 C, purple diamonds). Similarly, numerous spiralian protostomes (flatworms, annelids, mollusks, brachiopods, etc.) have several AMPAR subunits, some arising early in the protostome lineage and some arising later. For instance, an early duplication (Fig. 1 C, orange square) led to two daughter genes inherited by Brachiopoda (e.g., *L. anatina*), with one of these lost from other protostomes. More recently, both daughter genes duplicated in Brachiopoda (Fig. 1 C, yellow squares), giving rise to four genes in *L. anatina*.

This pattern of evolution is loosely reflected in Xenacoelomorpha, where, e.g., the xenoturbellid *Xenoturbella bocki*, the nemertodermatid *Meara stichopi*, and the acoel *H. miamia* each have multiple iGluR genes that are specific to Xenacoelomorpha (Fig. 1, B and C). However, there are two major clades of Xenacoelomorph AMPARs: clade 1 is closely related to nephrozoan (Protostomia + Deuterostomia) AMPARs, whereas clade 2 is more basally branching and found only in Xenacoelomorpha (Fig. 1 C). One interpretation of this is that the ancestral bilaterian may have had two AMPAR genes (Fig. 1 C, dark red star), and both were retained in xenacoelomorphs, but one (equivalent to clade 2) was lost in an early nephrozoan. Subsequently, a duplication of the clade 2 AMPAR gene after acoels diverged from other xenacoelomorphs (Fig. 1 C, light red star) has resulted in at least two clade 2 AMPAR genes in acoels, e.g., *Hofstenia*-98019615 and *Hofstenia*-98014323 in Fig. 1 C. A second possible explanation for this topology, however, is that the first bilaterian and then the proto-nephrozoan and proto-xenacoelomorph had only one AMPAR gene. And subsequently, a gene duplication in the proto-xenacoelomorph gave rise to clade 1 and 2 AMPAR genes, and the clade 2 gene diverged to the point where it branches basally in our tree. Although the former explanation is supported by similar branch lengths for the two clades in our analysis, definitively establishing whether early bilaterians had one or two AMPAR genes could be difficult. It presumably requires sequences from more numerous protostomes, which not only diverged from each other rapidly, but also underwent substantial independent rounds of gene gain and gene loss, making gene tree reconstruction difficult (Liebeskind et al., 2015; Howard et al., 2020; Serra Silva and Telford, 2026).

*Convolutriloba macropyga* (acoel) gene 17384.1 appears as a sister gene to other AMPARs (Fig. 1 C, toward the bottom). This could represent another early AMPAR gene that was lost in other bilaterians, but given its unique occurrence in this species, the gene would have to have been lost independently in other acoels, in nemertodermatids, in xenoturbellids, and in an early nephrozoan (Fig. 1 B), which seems unparsimonious. An alternate possibility is that *Convolutriloba* 17384.1 is related to clade 1 or 2 xenacoelomorph AMPARs but branches separately in our tree due to rapid evolution and divergence. *Convolutriloba* 17384.1 indeed diverges from other receptors in key amino acid positions (Fig. S3 B), but this could derive from either scenario.

There are exceptions to the pattern of recurrent, independent duplications; i.e., we see animals with only one AMPAR gene, indicating that AMPAR duplications have not occurred in certain lineages, or were not captured by the species we studied, or were outweighed by potential gene losses in those lineages. For example, the hemichordates *S. kowalevskii* and *Ptychodera flava* possess just a single hemichordate AMPAR gene, and only in fellow hemichordate *Schizocardium californicum* has a more recent duplication occurred (Fig. 1 C, white circle).

Thus, several AMPAR losses have occurred, e.g., in early nephrozoans and in early protostomes, and a few lineages retain just a single AMPAR gene, e.g., certain hemichordates. However, our analysis shows the family is dominated by recurrent, independent gene duplications. As a result, most bilaterians have two-to-six AMPAR genes, but those genes are relatively specific to that animal and its close relatives. This pattern contrasts with NMDARs, for example, where most bilaterians possess GluN1 and GluN2 subunits, both of which derive from a duplication that preceded the Bilateria/Cnidaria split, and consequently the GluN1 genes of most animals are highly orthologous (Ramos-Vicente et al., 2018; Stroebel and Paoletti, 2021).

### Convergence of diverse AMPARs on preferential heteromerization

**Figure 2.**
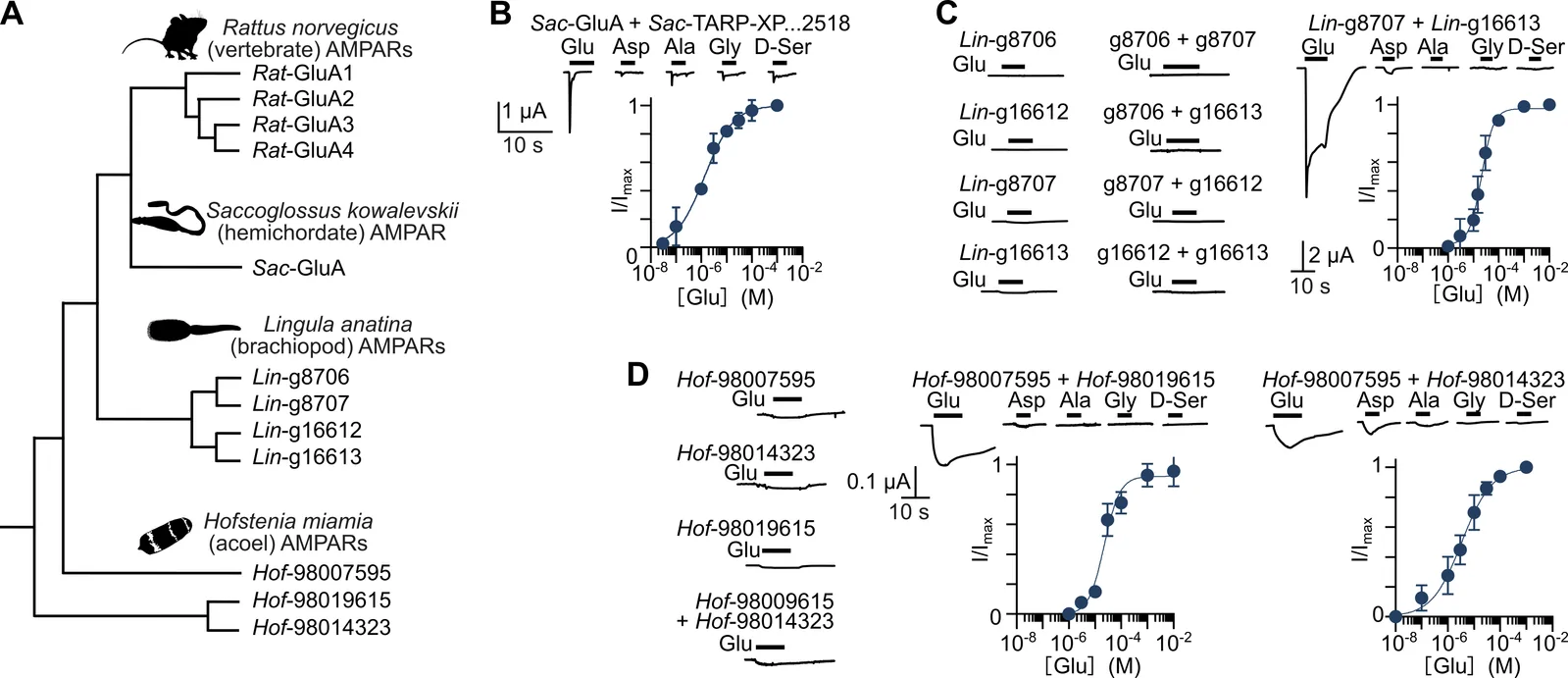
**Obligate heteromeric function of AMPAR genes that duplicated independently. (A)** Lineage-specific complements of AMPAR genes. **(B–D)** Current traces, example currents recorded from oocytes injected with single AMPAR cRNAs or combinations of different AMPAR cRNAs, as indicated, in response to ∼10 s application of 1 mM Glu, or other amino acids. Graphs, mean ± SD (*n* = 4–5) current amplitude (normalized to maximum current amplitude) in response to increasing concentrations of Glu at oocytes injected with indicated cRNAs. *Sac*-TARP-XP…2518 in B refers to XP_006822518.1.

Seeking insight into the functional ramifications of recurrent evolution of novel AMPAR subunits in different bilaterian lineages, we sought to heterologously express AMPAR genes from each of the three major lineages in *X. laevis* oocytes and measure their activity with two-electrode voltage clamp. We therefore studied the single gene from the hemichordate *S. kowalevskii*, all four genes from the spiralian (*sensu lato*) protostome *L. anatina*, and all three genes from the acoel xenacoelomorph *H. miamia* (Fig. 2 A and Fig. S4). *Saccoglossus* AMPAR gene *Sac*-GluA showed no functional expression in preliminary experiments (Fig. S5 A), so we tried co-expressing it with *Sac*-XP_006822518, a putative TARP, whose mammalian homologues enhance AMPAR expression (Jackson and Nicoll, 2011). When co-expressed with TARP-*Sac*-XP_006822518, *Sac*-GluA formed glutamate-gated channels reminiscent of previously characterized AMPARs, with rapid current activation, desensitization in the continued presence of glutamate, and a half-maximal excitatory glutamate concentration (EC_50_) of 1.5 ± 0.5 μM (here and elsewhere: mean ± SD; *n* = 4; Fig. 2 B). *Sac*-GluA receptor desensitization was complete, with no current remaining after ∼2 s in the presence of glutamate (Fig. 2 B).

Remarkably, the four protostome genes *Lin*-g8706, *Lin*-g8707, *Lin*-g16612, and *Lin*-g16613 failed to form ligand-gated channels on their own, but when co-injected, *Lin*-g8707 and *Lin*-g16613 successfully formed heteromeric channels specifically gated by glutamate, with an EC_50_ of 22 ± 0.3 µM (*n* = 5, Fig. 2 C). *Lin*-g8707/g16613 heteromeric receptor currents reflected those through *Rat*-GluA2 homomers in oocytes, with a small transient current and a more consistent sustained current (Fig. 2 C and Fig. S5 B). We also tested for higher-order heteromers by co-injecting oocytes with three or four cRNAs, each time injecting with the same total amount of cRNA. Co-injection of *Lin*-g8706 with *Lin*-g8707 and *Lin*-g16613 resulted in a slightly, but not significantly, smaller current amplitude than *Lin*-g8707/*Lin*-g16613, with no apparent change in glutamate potency (Fig. S5 C). We interpret this as expression of only *Lin*-g8707/*Lin*-g16613 heteromers upon co-injection of three cRNAs, perhaps at a lower level due to dilution of individual cRNAs. This tentatively suggests that *Lin*-g8706 cannot form tri-heterotetramers with these subunits. Upon co-injection of *Lin*-g16612 with *Lin*-g8707 and *Lin*-g16613, however, we observed no glutamate currents significantly greater than in water-injected oocytes (Fig. S5 C). Similarly, oocytes injected with all four *Lingula* AMPAR cRNAs showed no significant glutamate-gated currents (Fig. S5 C). No other combinations led to significant currents. Thus, we find solid evidence only for *Lin*-g8707/*Lin*-g16613 heteromers, although *Lin*-g16612 seems to reduce functional expression of other subunits. Whether this is via heteromerization and reduced surface expression, or via some other mechanism, we have not pursued.

The three xenacoelomorph genes *Hof*-98007595, *Hof*-98019615, and *Hof*-98014323 also failed to form detectable homomeric AMPARs on their own, but again, we found evidence for heteromeric receptors. Clade 1 *Hof*-98007595 formed heteromeric AMPARs with clade 2 *Hof*-98019615 and with clade 2 *Hof*-98014323, whereas closely related *Hof*-98019615 and *Hof*-98014323 failed to form functional AMPARs with each other (Fig. 2, A and D). Oocytes expressing *Hof*-98007595/98019615 heteromers showed larger glutamate-gated currents than oocytes expressing *Hof*-98007595/98014323 heteromers, but glutamate EC_50_ values were similar for both combinations (24 ± 2 µM and 38 ± 1 µM, respectively; both *n* = 5; Fig. 2 D). Cyclothiazide, which binds between adjacent LBDs in rat AMPARs to impair desensitization and thus increase current amplitude (Sun et al., 2002), had no effect on *Saccoglossus*, *Lingula*, or *Hofstenia* receptors (Fig. S5 D).

Heterologous expression of a maximally diverse set of AMPAR genes thus indicates that throughout Bilateria, AMPARs appear to mediate fast responses specifically to glutamate (Fig. 2, B–D). Moreover, the data suggest that during the evolution of the different bilaterian lineages, when AMPAR genes duplicated, the new genes rapidly diversified in a complementary manner, such that heteromerization of two genes became a requirement for functional receptors, which is supported by native co-expression of the complementary genes in different cell types in *Hofstenia* (Hulett et al., 2023).

### Sub-functionalization of novel AMPAR subunits

**Figure 3.**
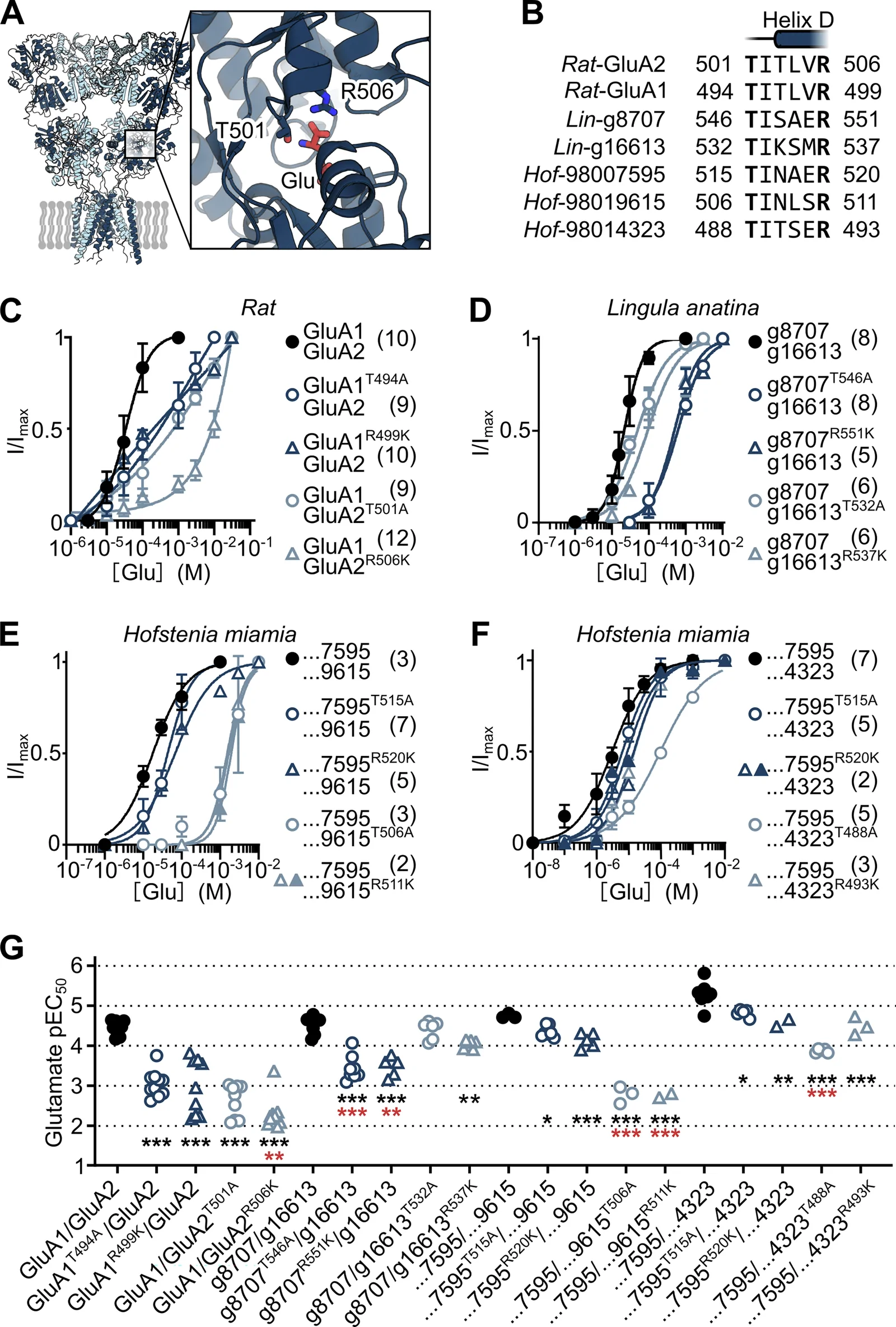
**Novel subunits make different functional contributions to heteromeric AMPARs. (A)** Full-length AMPAR (left, PDB accession no. 5WEO [Twomey et al., 2017]) and *Rat*-GluA2 LBD X-ray structure (right, PDB accession no. 1FTJ [Armstrong and Gouaux, 2000]). **(B)** Selected *Rat*-GluA2 ligand-binding residues (bold) aligned with other receptor subunits (full alignment in Fig. S4). **(C–F)** Mean (±SD, *n* in brackets in legends) normalized (to maximum in each recording) current responses to increasing concentrations of glutamate for indicated AMPAR subunit combinations (mutations in superscript). **(G)** pEC_50_ values from experiments in C–F. WT and cognate mutants were compared with one-way ANOVA and Tukey’s multiple comparisons test, where *P < 0.05, **P < 0.01, and ***P < 0.001 compared with WT (black asterisks) or compared with homologous mutation in other subunit (red asterisks). *n* values shown in parentheses in C–F.

Questioning whether novel AMPAR subunits adopt different functional roles after their emergence, we tested whether mutations of glutamate-binding residues in the LBD are more detrimental to channel activity in either of the two subunits of heterotetramers (Fig. 3, A and B). We first tested our approach with *Rat*-GluA1/GluA2 heterotetramers, given the knowledge that *Rat*-GluA1 and *Rat*-GluA2 preferentially heteromerize, and the GluA2 subunit prefers the B/D positions and undergoes greater glutamate-induced conformational changes during gating (Twomey et al., 2017; Herguedas et al., 2019; Zhao et al., 2019; Yu et al., 2021). We observed that T501A and R506K mutations in the GluA2 subunit shifted the heterotetramer EC_50_ from 38 ± 16 µM (*n* = 10) to 1.1 ± 0.2 mM and 6.5 ± 0.5 mM (*n* = 9 and 12). In contrast, equivalent T494A and R499K mutations in the GluA1 subunit shifted the heterotetramer EC_50_ only half as much, to 0.58 ± 0.07 mM and 2.6 ± 0.9 mM (*n* = 9 and 10), although the difference in pEC_50_ values (used for comparison with normal distributions) between homologous mutations in different subunits was only significant for the R-K mutation (Fig. 3, C and G, red asterisks). We are confident that this assay recorded currents of mostly heteromers rather than unmutated homomers, because, for example, whereas WT GluA2 homomers responded to micromolar glutamate and R499K mutant GluA1 homomers barely responded to even 10 and 30 mM glutamate, cells expressing WT GluA2 and R499K GluA1 together responded to high micromolar and low millimolar glutamate with responses saturating between 3 and 10 mM, indicating a unique and dominant population of heteromeric receptors (Fig. S5 E). These data tentatively suggest that decreased glutamate potency in the GluA2 subunits of heterotetramers is more detrimental to channel activation than in GluA1 subunits, consistent with GluA2 occupation of the B/D positions in the tetramer (Herguedas et al., 2019; Zhao et al., 2019).

In protostome and xenacoelomorph obligate heterotetramers, the trend was even more pronounced. In protostome *Lin*-g8707/g16613 AMPARs (WT glutamate EC_50_ = 33 ± 16 µM, *n* = 8), T546A and R551K mutations in *Lin*-g8707 caused 12- to 14-fold decreases in glutamate potency (EC_50_ 480 ± 70 µM and 380 ± 20 µM, *n* = 8 and 5), whereas the equivalent T532A and R537K mutations in *Lin*-g16613 caused relatively modest or no decreases in glutamate potency (EC_50_ 36 ± 13 µM and 91 ± 23 µM, both *n* = 6; difference in pEC_50_ values between homologous mutations in different subunits P < 0.001 for T-A and P < 0.01 for R-K mutations, Fig. 3, D and G). Similarly, in xenacoelomorph *Hof*-98007595/98019615 AMPARs, mutations in the *Hof*-98019615 subunit caused 100-fold decreases in glutamate potency (EC_50_ 18 ± 2 µM, *n* = 3 at WT, 1.8 ± 0.9 mM and 1.8 ± 0.3 mM, *n* = 3 and 2 at mutants), whereas the equivalent mutations in *Hof*-98007595 caused only 3-fold to 4-fold decreases in glutamate potency (EC_50_ 47 ± 14 µM and 79 ± 34 µM, *n* = 7 and 5 at mutants; difference in pEC_50_ values between homologous mutations in different subunits P < 0.001 for T-A and R-K mutations, Fig. 3, E and G). This trend continued in *Hof*-98007595/98014323 AMPARs, where *Hof*-98014323 mutations were generally more detrimental to glutamate potency than *Hof*-98007595 mutations (Fig. 3 F), although this was only significant for the T-A mutation (P < 0.001, Fig. 3 G) and was more difficult to determine in *Hof*-98007595/98014323 AMPARs due to smaller glutamate-gated currents (Fig. S5 F). Notably, this indicates that although *Hof*-98007595 is the obligatory subunit in the two xenacoelomorph heterotetramers, it does not dominate channel activation by glutamate. Together, these data point to the trend that when heteromeric AMPARs emerge from a gene duplication, one of the subunits may adopt a more dominant role in channel activation by glutamate.

### Independent evolution of voltage-independent AMPAR currents via similar biophysical mechanisms

**Figure 4.**
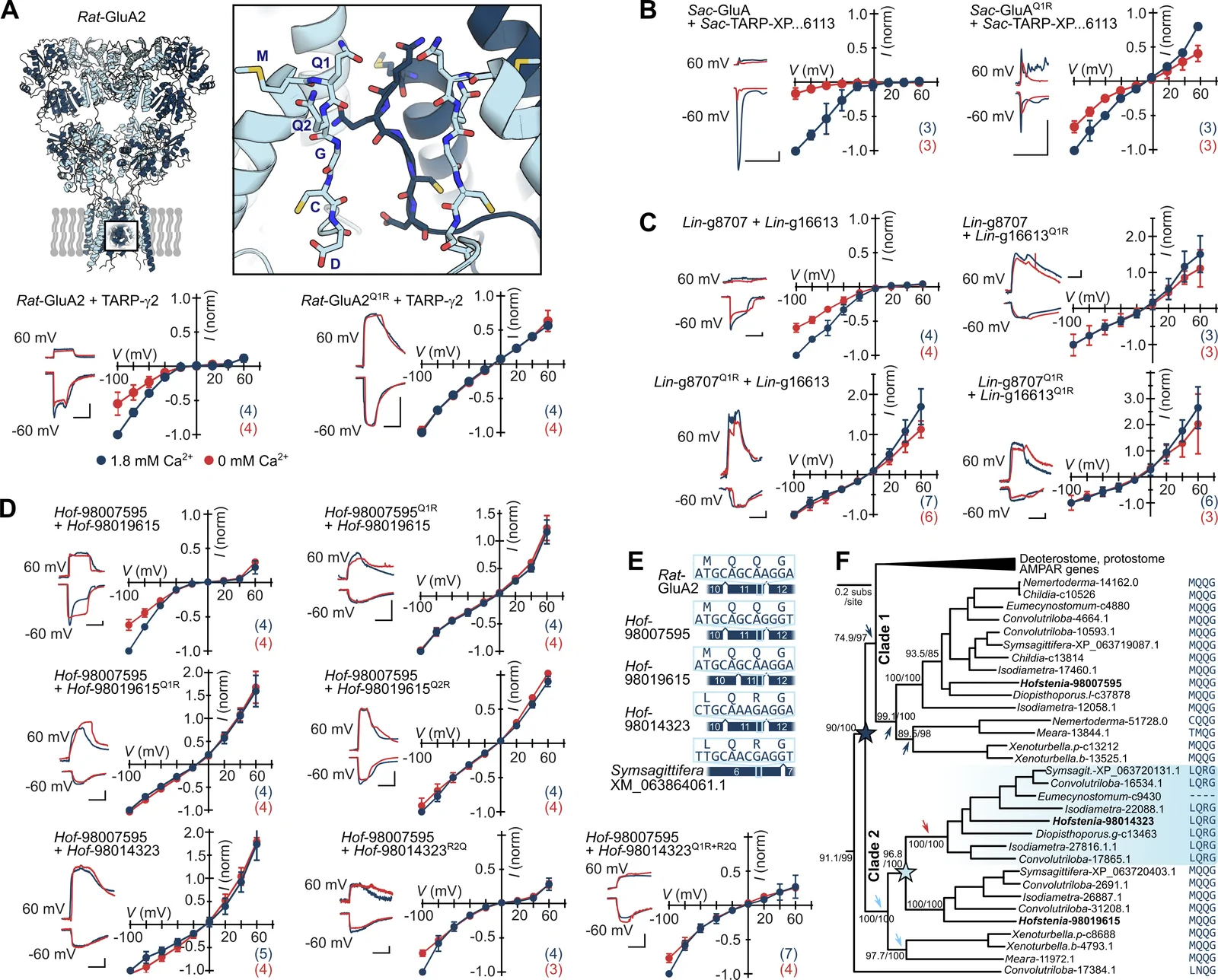
**Independent evolution of voltage-independent currents in acoel AMPARs. (A)** Main, lower half of the channel pore in *Rat*-GluA2 AMPARs, subunits shaded in blue, one subunit omitted from foreground, selected amino acid residues labeled in one subunit. TARP-y2 subunits omitted for clarity. From PDB accession no. 5WEO. **(A–D)** Current traces, example currents at indicated membrane potentials with Ca^2+^-free (red) and 1.8 mM Ca^2+^-containing (blue) extracellular solutions, and graphs, normalized current amplitude (I)—membrane potential (V) relationships for indicated AMPAR subunit combinations (data points mean ± SD, connected by straight lines, *n* in brackets) for vertebrate deuterostome (A), invertebrate deuterostome (B), protostome (C), and xenacoelomorph (D) AMPARs. *Sac*-TARP-XP…6113 in B refers to XP_006816113.1, which was used here because currents through *Sac*-GluA^Q1R^ + TARP-XP_006822518.1 were small and difficult to work with. Scale bars: x = 10 s; y = 1 µA (A and B) or 0.1 µA (C and D). We co-expressed human TARP-γ2 with *Rat*-GluA2, as homotetrameric *Rat*-GluA2^Q1R^ mutant channels showed no glutamate-gated currents (Fig. S5 G). **(E)** Exons (dark blue bars) encoding pore-lining residues in selected AMPAR genes from rat and from acoels *H. miamia* and *Symsagittifera roscofensis*. White labels indicate exon number within each gene. For the highlighted portion of exon 11 or exon 6, the unedited nucleotide sequence and amino acid sequence are shown above. **(F)** Acoel AMPAR branch from AMPA gene tree (Fig. S1), including alignment of pore-lining residues (right), with *Hofstenia*-98014323 orthologues highlighted in light blue. Stars, arrows: gene duplications, mutations, or speciation events referred to in the main text. Gaps in alignment; the Eumecynostomum-c9430 sequence was partial.

Mammalian AMPARs of different subunit compositions have different I-V relationships. GluA2-containing receptors in many cortical principal neurons conduct small currents that increase in amplitude linearly with membrane potential, whereas GluA2-lacking AMPARs in numerous other cell types conduct large inward currents at negative potentials and relatively small outward currents between 0 and 40 mV (inward rectification) (Geiger et al., 1995; Cull-Candy and Farrant, 2021; Miguez-Cabello et al., 2025). The small currents and linear I-V in GluA2-containing receptors relate to decreased Na^+^ and K^+^ conductance, loss of Ca^2+^ permeation, and loss of block by intracellular polyamines at positive potentials due to a positively charged arginine residue instead of a neutral glutamine at the “Q1” position (Fig. 4 A; Cull-Candy and Farrant, 2021). AMPAR currents are also further tuned by auxiliary proteins, Ca^2+^ block, and allosteric modulation by extracellular cations (Kalappa et al., 2015; Dawe et al., 2016; Miguez-Cabello et al., 2025). A detailed study of any of these factors in AMPAR current would be a substantial undertaking. But simply measuring glutamate-gated I-V relationships of heterologously expressed AMPARs in different extracellular ions offers a qualitative assessment of the pore properties of different AMPAR complexes (Hollmann et al., 1991; Li et al., 2016). This often reflects the activity of native receptors (Geiger et al., 1995; Miguez-Cabello et al., 2025). We therefore measured glutamate-gated I-V relationships in diverse AMPARs to further investigate sub-functionalization of novel AMPAR genes. For each AMPAR, we measured I-V curves in the presence and absence of Ca^2+^ to test if Ca^2+^ affects the I-V properties as it does in rat AMPARs. We also introduced arginine at the Q1 position in the pore of each subunit (Fig. 4 A), to test if its presence would change the amplitudes and rectification properties of the currents the same way it does for rat AMPARs. As we could not detect currents in mutant *Rat*-GluA2^Q1R^ channels, we co-expressed *Rat*-GluA2 channels with human TARP-γ2 (Fig. S5 G; the human cDNA was available to us).

The homomeric *Saccoglossus* AMPAR/TARP complex behaved similarly to rat AMPARs, although inward rectification and larger currents in the presence of Ca^2+^ were even more noticeable in the unmutated *Saccoglossus* complex. The block was relieved to a slightly lesser extent by the engineered Q1R mutation compared with the rat GluA2/TARP-y2 complex (Fig. 4 B). Similarly, the heteromeric *Lingula* AMPAR showed inward rectification and larger currents in the presence of Ca^2+^ than in the absence of Ca^2+^, and here the Q1R mutation in either subunit of the heteromer was enough to abolish these properties (Fig. 4 C), supporting the notion that our recordings detect primarily heteromeric receptors.

Curiously, when we examined *H. miamia* AMPARs, we observed substantial differences between the two heteromers in their I-V relationships. The *Hof*-98007595/98019615 AMPAR was very similar to nephrozoan (deuterostome + protostome) AMPARs, with inward rectification and larger currents in the presence of Ca^2+^, and both properties were abolished by Q1R mutations in either the *Hof*-98007595 or *Hof*-98019615 subunit (Fig. 4 D, first three plots). In stark contrast, the WT *Hof*-98007595/98014323 AMPAR showed no difference in current amplitude with/without Ca^2+^ and showed a relatively linear I-V relationship (Fig. 4 D, bottom-left). We examined the amino acid sequences of the different subunits looking for a potential explanation for these properties. Although the WT *Hof*-98014323 subunit has a glutamine at the Q1 position, it has an arginine at the Q2 position (Fig. 4 E), which could prevent potential Ca^2+^ permeation and polyamine block. Remarkably, this arginine is encoded by genomic DNA in the *Hof*-98014323 gene, as opposed to *Rat*-GluA2, where the genomic sequence encodes Q1 and Q2 and relies on RNA editing for an R1 residue in the pore (Fig. 4 E). The *Hof*-98014323 R2Q mutation conferred strong inward rectification and a small but noticeable increase in current amplitude in the presence of Ca^2+^ on the *Hof*-98007595/98014323 AMPAR (Fig. 4 D, lower-mid plot), confirming that the R2 residue determines the voltage-independent currents in WT receptors. Interestingly, introducing a Q1R mutation onto the R2Q mutant *Hof*-98007595/98014323 receptor did not reinstate WT-like I-V linearity (Fig. 4 D, bottom-right), suggesting that in this receptor, these properties are more tunable at the second position.

Questioning the evolution of the two AMPAR types in *Hofstenia*—linear I-V and Ca^2+^-insensitive vs. inwardly rectifying and Ca^2+^-sensitive—we returned to our phylogenetic tree. The tree suggests that xenacoelomorphs retained two early bilaterian AMPAR genes, clade 1 and clade 2, both containing Q1 and Q2 residues in the channel pore (Fig. 4 F, dark blue star). The clade 1 gene, homologous to *Hof*-98007595, the obligatory subunit in *Hofstenia* heteromers, was inherited and retained by the three major xenacoelomorph lineages after their divergence (Fig. 4 F, dark blue arrows) and by nephrozoans. The clade 2 gene was also inherited by all xenacoelomorphs upon their divergence (Fig. 4 E, light blue arrows) but was lost in nephrozoans. Only after Acoela diverged from Xenoturbellida and Nemertodermatida, however, the clade 2 gene duplicated in an ancestral acoel (Fig. 4 F, light blue star). This gave rise to *Hof*-98019615 and *Hof*-98014323 orthologues present in most extant acoels. Shortly after this duplication, one of the resulting genes promptly underwent mutation(s) (Fig. 4 F, red arrow), leading to *Hof*-98014323 orthologues containing an R2 residue (Fig. 4 F, alignment). Thus, an early AMPAR gene duplication in Xenacoelomorpha gave rise to two new subunits. One became relatively passive in channel activation by glutamate but obligatory for heterotetramer assembly. The other became dominant in channel activation and then underwent another duplication. This later duplication gave rise to one subunit contributing to heterotetramers with inwardly rectifying currents that are bigger in the presence of Ca^2+^ and another subunit that forms heterotetramers with linear I-V properties and no apparent effects of Ca^2+^.

### Auxiliary proteins increase the functional diversity of each animal’s AMPAR complement

**Figure 5.**
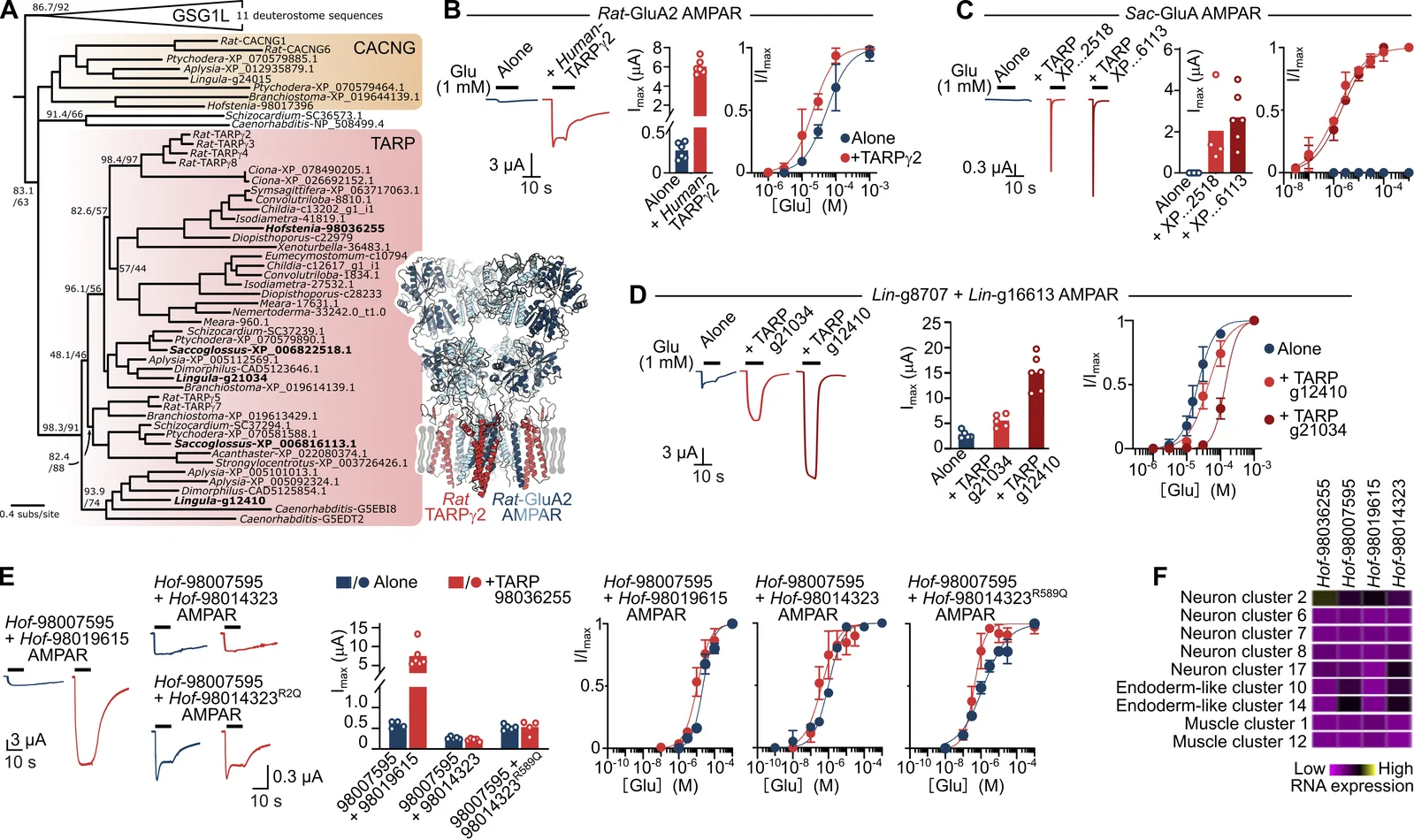
**Modulation of AMPAR function by auxiliary proteins. (A)** TARP branch of a TARP/CACNG/GSG1L gene tree (full tree in Fig. S6). Genes tested here are in bold. Inset structure (PDB accession no. 5WEO) shows *Rat*-TARPγ2 in complex with *Rat*-GluA2 AMPAR. **(B–E)** Left, example currents; middle, maximum current amplitude (columns—mean, dots—data from different oocytes); and right, mean ± SD (*n* = 4) current amplitude (normalized to maximum) in response to increasing concentrations of glutamate at oocytes injected with indicated cRNAs: AMPAR alone, or AMPAR with indicated TARP. *Sac*-TARP-XP…2518 and …6113 in C refer to TARP-XP_006822518.1 and XP_006816113.1. **(F)** Relative expression of *H. miamia* TARP (*Hof*-98036255) and AMPAR subunit (other genes) RNAs in indicated cell clusters from publicly available single-cell RNA sequencing data (Hulett et al., 2023).

AMPAR expression and function are profoundly affected by TARPs, four-helical membrane proteins that physically interact with AMPARs and postsynaptic scaffolding proteins (Fig. 5 A, inset structure). Mammalian type I TARPs, such as TARP-γ2 (or “stargazin”), tend to increase surface expression, decrease desensitization, and increase glutamate potency at AMPARs (Fig. 5 B), and mammalian type II TARPs (TARP-γ5 and TARP-γ7) affect surface expression less dramatically and tend to decrease or not affect glutamate potency (Jackson and Nicoll, 2011). TARPs from *C. elegans*, *D. melanogaster*, and *A. mellifera* (ecdysozoan protostomes) and *C. intestinalis* (an invertebrate deuterostome) appear to greatly increase heterologous surface expression of cognate AMPARs, which otherwise express barely at all (Walker et al., 2006; Hirai et al., 2017; Brunello et al., 2022). We therefore considered whether the function of diverse deuterostome, protostome, and xenacoelomorph AMPARs differs in the presence of cognate TARPs.

Our phylogeny of TARPs and their close relatives, also including genes of the Xenacoelomorpha lineage, confirms a previous report that the TARP family is specific to bilaterians (Ramos-Vicente and Bayes, 2020). Also, like that study, we find poor support for many branches in the TARP tree relative to the AMPAR tree. The TARP branch in our tree has SH-aLRT and ultrafast bootstrap branch support values of 98.3 and 91—very high and modestly high for those methods, respectively (Guindon et al., 2010; Hoang et al., 2018). But within the TARP branch, support for three branches of interest is much lower. A branch including vertebrate type I TARPs, all xenacoelomorph TARPs, and several other deuterostome and protostome TARPs has branch support values of 96.1 and 56. A branch including vertebrate Type II TARPs and several other deuterostome TARPs has support values of 82.4 and 88. And a basally branching TARP-like branch of protostome sequences has support values of 93.9 and 74. A phylogeny based on an untrimmed alignment only returned two of these three “sub-branches” but returned the same TARP branch (Figs. S6 and S7), and we therefore considered all genes in this overarching branch as TARPs (red in Fig. 5 A), but we treat with caution the relationships of other TARPs to vertebrate type I and II TARPs.

We identify two putative TARPs in the deuterostome *S. kowalevskii*: *Sac*-XP_006822518 appears in the vertebrate type I branch, and *Sac*-XP_006816113 appears in the vertebrate type II branch, both with orthologues in other hemichordates (Fig. 5 A). In our experiments, AMPAR expression was drastically increased in the presence of both TARPs, as we could in fact only measure glutamate-gated currents when either TARP was present (Fig. 5 C). Both TARPs led to similar-sized macroscopic currents and similar glutamate potency (Fig. 5 C), with EC_50_ values of 1.5 ± 0.5 µM (*n* = 4) and 2.0 ± 0.5 µM (*n* = 6). With the caveat of low branch support described above, this shows that certain deuterostome TARPs of the type II branch can greatly enhance expression or function, unlike mammalian Type II TARPs.

The spiralian protostome *L. anatina* also has two TARPs. TARP *Lin*-g21034 has homologues in most other protostomes and appears related to type I vertebrate TARPs, but this relationship is only supported by one of our confidence methods (Fig. 5 A). TARP *Lin*-g12410 also has homologues in numerous other protostomes, including previously characterized *C*. *elegans* STG-2 (G5EBI8 in Fig. 5 A). *Lin*-g21034 increased maximum current amplitude in *Lin*-g8707/g16613 heteromeric AMPARs ∼5-fold, with a modest, 2-fold decrease in glutamate potency (EC_50_ 46 ± 15 µM, *n* = 10), whereas *Lin*-g12410 increased maximum current amplitude ∼15-fold and caused a 6-fold decrease in glutamate potency (EC_50_ 130 ± 30 µM, *n* = 7; Fig. 5 D). Xenacoelomorph *H. miamia* has only one TARP, *Hof*-98036255, with homologues in other xenacoelomorphs, and from our analysis it’s difficult to conclude its relationship to previously characterized TARPs (Fig. 5 A, Fig. S6, and Fig. S7). When co-expressed with *Hof*-98007595/98019615 AMPARs, *Hof*-98036255 caused a large increase in current amplitude and little, if any, change in glutamate potency (EC_50_ with TARP 12 ± 2 µM, *n* = 6, compared with 24 ± 2 µM without TARP, *n* = 5; Fig. 5 E). In contrast, the activity of the second heteromer, *Hof*-98007595/98014323, was not altered by TARP *Hof*-98036255, although current amplitude was generally small in this heteromer and more difficult to assess (Fig. 5 E). This led us to test TARP modulation of Ca^2+^-sensitive *Hof*-98007595/98014323^R2Q^ mutant AMPARs, but despite larger currents, we again observed no apparent modulation of receptor function via TARP *Hof*-98036255 (Fig. 5 E).

Notably, when we searched an existing single-cell RNA sequencing dataset of *H*. *miamia*, we saw that TARP *Hof*-98036255 is highly expressed by one particular cluster of putative neurons, and this is the cluster in which AMPAR subunits *Hof*-98007595 and -98019615 are highly co-expressed (Fig. 5 F; Hulett et al., 2023). In contrast, *Hof*-98007595 and -98014323 are co-expressed in cell clusters with endoderm-like transcription profiles and without TARP *Hof*-98036255 expression (Fig. 5 F). Thus, one set of neurons in *Hofstenia* seems to express inwardly rectifying, Ca^2+^-sensitive, TARP-modulated AMPARs, and another set of cells expresses voltage-independent, Ca^2+^-insensitive AMPARs in the absence of TARPs.

### Stark pharmacological differences between diverse AMPARs

AMPA receptor pharmacology is important as a tool for isolating different iGluR populations in physiological experiments, for the design of selective modulators and potential therapies, and historically for iGluR nomenclature. We therefore investigated the pharmacological properties of diverse bilaterian AMPARs, starting with classical synthetic agonists that lend their name to mammalian iGluRs.

**Figure 6.**
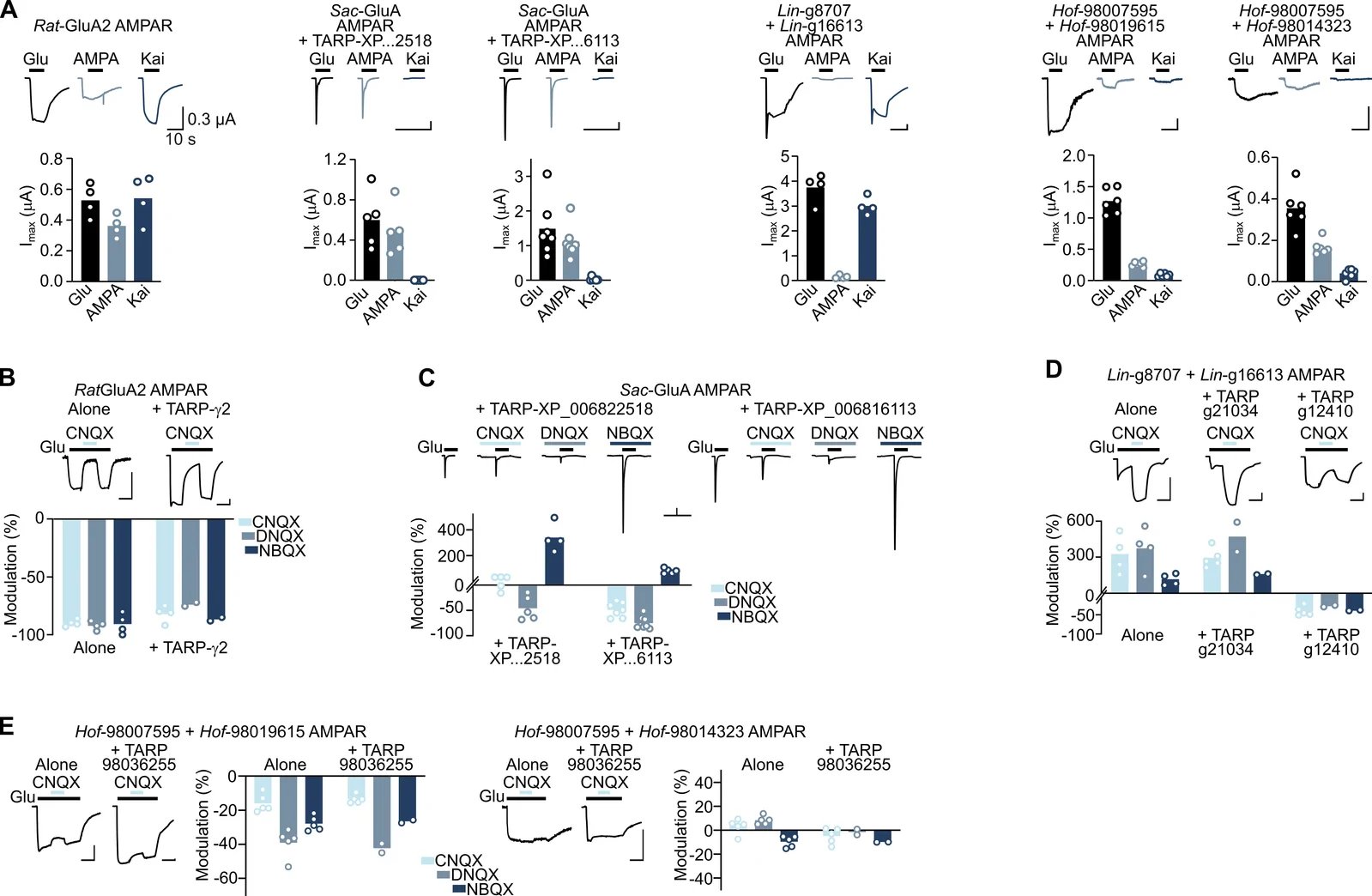
**AMPAR pharmacology. (A)** Example (upper panels) and individual data points and means (dots and columns in lower panels) for indicated agonist-gated currents in oocytes expressing indicated AMPARs and TARPs from *R. norvegicus* (*Rat*, *n* = 4), *S. kowalevskii* (*Sac*, *n* = 5–7), *L. anatina* (*Lin*, *n* = 4), and *H. miamia* (*Hof*, *n* = 6). **(B–E)** Upper or left panels, example glutamate-gated current in absence and presence of indicated drugs. Lower or right panels, data points and means (dots and columns) of percentage of modulation of glutamate-gated current by CNQX, DNQX, and NBQX (*n* = 2–5 as shown). “−100% modulation” equates to complete inhibition. 100% modulation equates to doubling of current amplitude. *Sac*-TARP-XP…2518 and …6113 in A and C refer to TARP-XP_006822518.1 and XP_006816113.1.

The *Saccoglossus* AMPAR-GluA/TARP-XP_006822518.1 and AMPAR-GluA/TARP-XP_006816113.1 complexes are, indeed, strongly activated by AMPA, which elicits current amplitudes that are 81 ± 6% and 78 ± 8% that of glutamate (*n* = 5 and 6), respectively. At the same time, kainate generates barely detectable currents at these complexes (*n* = 5 and 6; Fig. 6 A). AMPA also activated larger currents than kainate at both of the *Hofstenia* AMPARs, although AMPA- and kainate-gated currents were both much smaller than glutamate-gated currents (AMPA 21 ± 2% and kainate 7 ± 2% at *Hof*-98007595/98019615, both *n* = 6; AMPA 50 ± 12% and kainate 17 ± 12% at *Hof*-98007595/98014323, both *n* = 8; Fig. 6 A). In contrast to deuterostome and xenacoelomorph AMPARs, protostome *Lingula* heteromeric AMPARs were more responsive to kainate. Here, kainate-gated current amplitude was 92 ± 25% that of glutamate, and AMPA was 10 ± 13% (both *n* = 5; Fig. 6 A). This is similar to fellow protostome *D. melanogaster* AMPARs (Ultsch et al., 1992; Li et al., 2016), and these results suggest that among synthetic agonists, AMPA is relatively efficacious at most bilaterian AMPARs, but protostome AMPARs have evolved such that they show smaller responses to AMPA than to kainate.

Quinoxalinedione compounds CNQX, DNQX, and NBQX are classical competitive antagonists of AMPARs that bind to the same site as glutamate. They potently inhibit glutamate-induced activation of mammalian AMPARs and are thus used as tools to isolate NMDAR currents, although when applied alone to AMPAR/TARP complexes, CNQX and DNQX can activate small currents (Menuz et al., 2007). As expected, when co-applied with glutamate during glutamate-gated currents in *Rat*-GluA2 homotetramers, all three compounds caused nearly 100% inhibition both in the absence or presence of TARP-γ2 (Fig. 6 B). Inhibition of *Rat*-GluA2 receptors was similar when the inhibitor was pre-applied and then glutamate was co-applied (Fig. S8, A and B), and we used this protocol to assess inhibition of *Saccoglossus* AMPARs, which showed no sustained current (Fig. 6 C). DNQX inhibited 47 ± 24% and 74 ± 12% of the current through *Saccoglossus* AMPAR-GluA/TARP-2518 and AMPAR-GluA/TARP-6113 complexes, respectively (*n* = 5 and 8). In contrast, CNQX had no effect on AMPAR-GluA/TARP-2518 and inhibited only the AMPAR-GluA/TARP-6113 complex (48 ± 15%, *n* = 7), whereas NBQX enhanced currents 359 ± 111% and 99 ± 20% in AMPAR-GluA/TARP-2518 and AMPAR-GluA/TARP-6113 complexes (*n* = 4 and 5; Fig. 6 C).

We also observed that all three compounds enhanced glutamate-gated currents in *Lin*-g8707/g16613 heterotetrameric AMPARs, even in the absence of TARPs, with 321 ± 161% and 369 ± 174% enhancement by CNQX and DNQX and 108 ± 47% enhancement by NBQX (each *n* = 4; Fig. 6 D). In the presence of TARP *Lin*-g21034, which may be related to mammalian TARP-γ2, the pattern of enhancement was very similar to that without TARPs (Fig. 6 D). But remarkably, the presence of protostome-specific TARP *Lin*-g12410 converted enhancement to 32–45% inhibition by all three compounds (*n* = 5 for CNQX, *n* = 2 for DNQX and NBQX; Fig. 6 D). *Hofstenia* AMPARs were generally less sensitive to all three compounds, with 15 ± 8%, 39 ± 8%, and 29 ± 4% inhibition of *Hof*-98007595/98019615 AMPARs by CNQX, DNQX, and NBQX, respectively (each *n* = 5), and similar effects in the presence of TARP *Hof*-98036255 (Fig. 6 E). Compounds showed little if any modulation of *Hof*-98007595/98014323 AMPARs (each *n* = 5), regardless of co-expression with TARP *Hof*-98036255 (*n* = 5 for CNQX, *n* = 2 for DNQX and NBQX; Fig. 6 E). As with rat receptors, the inhibitory or enhancing effects of CNQX, DNQX, and NBQX on *Lingula* and *Hofstenia* AMPARs were similar when the compounds were co-applied after glutamate pre-application or when pre-applied, and then glutamate was co-applied (Fig. S8, D–G). Pre-application of the compounds also showed that NBQX, to a lesser extent DNQX, and still less CNQX, activated small currents in *Lingula* AMPARs with and without TARPs, and in *Hofstenia* AMPARs with TARPs (Fig. S8, D–G). This contrasts with rat AMPARs, which are only activated by CNQX and DNQX, and not NBQX, in the presence of TARPs (Menuz et al., 2007). The *Saccoglossus* AMPAR, when co-expressed with TARPs, was activated by all three, although more consistently by CNQX (Fig. S8 C).

These experiments show that early-branching xenacoelomorph AMPARs, like classical mammalian AMPARs, are activated more efficaciously by AMPA than by kainate. It therefore seems fitting that this family bears the AMPAR name, despite the fact that in the protostome lineage there are several AMPARs that are more efficaciously activated by kainate. We also learn, however, that classical competitive antagonists act as potentiators and weak agonists at certain AMPARs, and care should therefore be taken when trying to isolate non-AMPARs in physiological experiments via the use of such compounds.

### Diverse heterotetrameric AMPARs are suited to fast synaptic signaling

**Figure 7.**
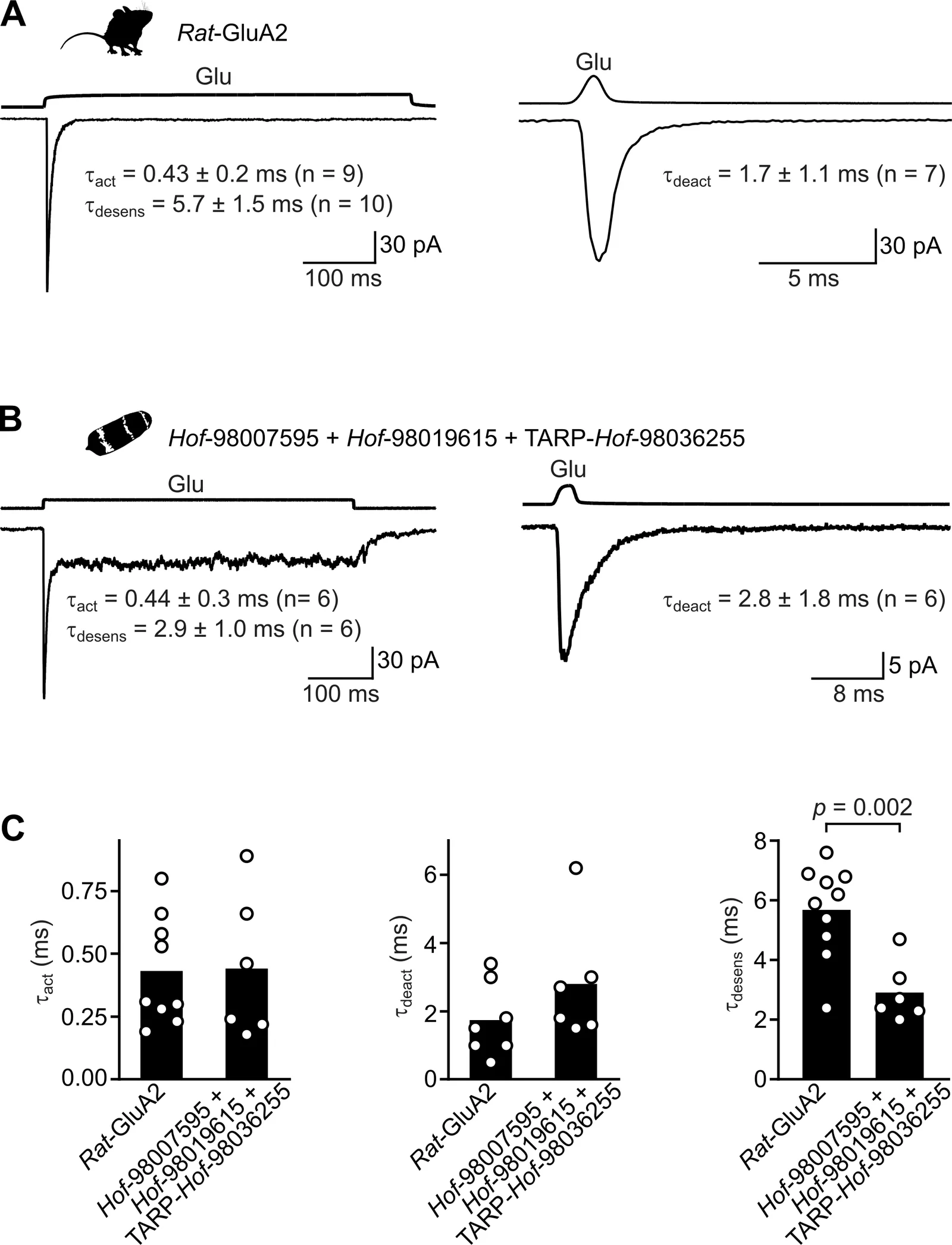
**Fast activation and deactivation of diverse AMPARs. (A and B)** Outside-out patch-clamp recordings of rat and *H. miamia* AMPARs in HEK cells. Left, desensitizing currents in response to 500 ms application of glutamate (10 mM). Right, deactivating currents in response to a 1 ms pulse of glutamate (10 mM). Holding potential was −60 mV. **(C)** Summary of monoexponential fits to currents, such as the ones shown in A and B. Time constants for activation, desensitization, and deactivation (τ_act_, τ_desens_, and τ_deact_) are mean ± SD. P value from two-tailed unpaired *t* test.

Our results point toward obligate heteromerization and selective activation by glutamate throughout the AMPAR family. But our macroscopic experiments with oocytes lack fast solution exchange and thus preclude the measurement of diverse AMPAR responses to brief glutamate exposure and thus their propensity for fast synaptic signaling. We therefore turned to outside-out patches from HEK293 cells and fast solution exchange to measure the kinetics of AMPAR function. As expected, rat AMPARs showed rapid kinetics, with currents activating in less than a millisecond, and deactivating after glutamate removal or desensitizing in the presence of glutamate within a few milliseconds (Fig. 7, A and C).

We attempted to record currents from *Saccoglossus* and *Lingula* AMPARs by transfecting HEK cells with *Sac*-GluA; *Sac*-GluA and TARP-XP_006822518; *Sac*-GluA and TARP-XP_006816113; *Lin*-8707 and g16613; *Lin*-g8707, g16613, and TARP*-*g21034; and *Lin*-g8707, g16613m, and TARP-g12410. For each of these 6 conditions, 9 to 10 outside-out patches were obtained over 3 or more separate transfections; however, no measurable currents were detected (Table S5). We also tried recording in whole-cell mode but detected no currents. As all plasmids encoded IRES eGFP, it was not possible to ascertain whether the absence of current reflected the misfolding of proteins, unsuccessful uptake of all plasmids, or the absence of a fully assembled receptor at the plasma membrane.

Patches containing *Hof*-98007595/98019615 heterotetramers together with TARP-*Hof*-98036255 showed glutamate-gated currents, allowing us to compare this early branching xenacoelomorph complex with the mammalian homologue (Fig. 7 B). Time constants for activation and deactivation were 0.44 ± 0.3 ms and 2.8 ± 1.8 ms (*n* = 6), respectively, and we observed rapid desensitization in the continued presence of glutamate, with a time constant of 2.9 ± 1.0 ms (*n* = 6). Remarkably, desensitization of *Hofstenia* complexes appears as fast as deactivation and faster than in *Rat*-GluA2 receptors (P = 0.002, *n* = 10 for rat and *n* = 6 for *Hofstenia*). Time courses of activation and deactivation were not significantly different from the *Rat*-GluA2 AMPAR (Fig. 7 C) (P = 0.94 and 0.21, respectively). Despite some currents exhibiting a partial block of desensitization—consistent with rat AMPAR modulation by, e.g., TARPγ2—other recordings showed complete desensitization: in these instances, TARP-*Hof*-98036255 may not be associated with the final receptor assembly.

**Figure 8.**
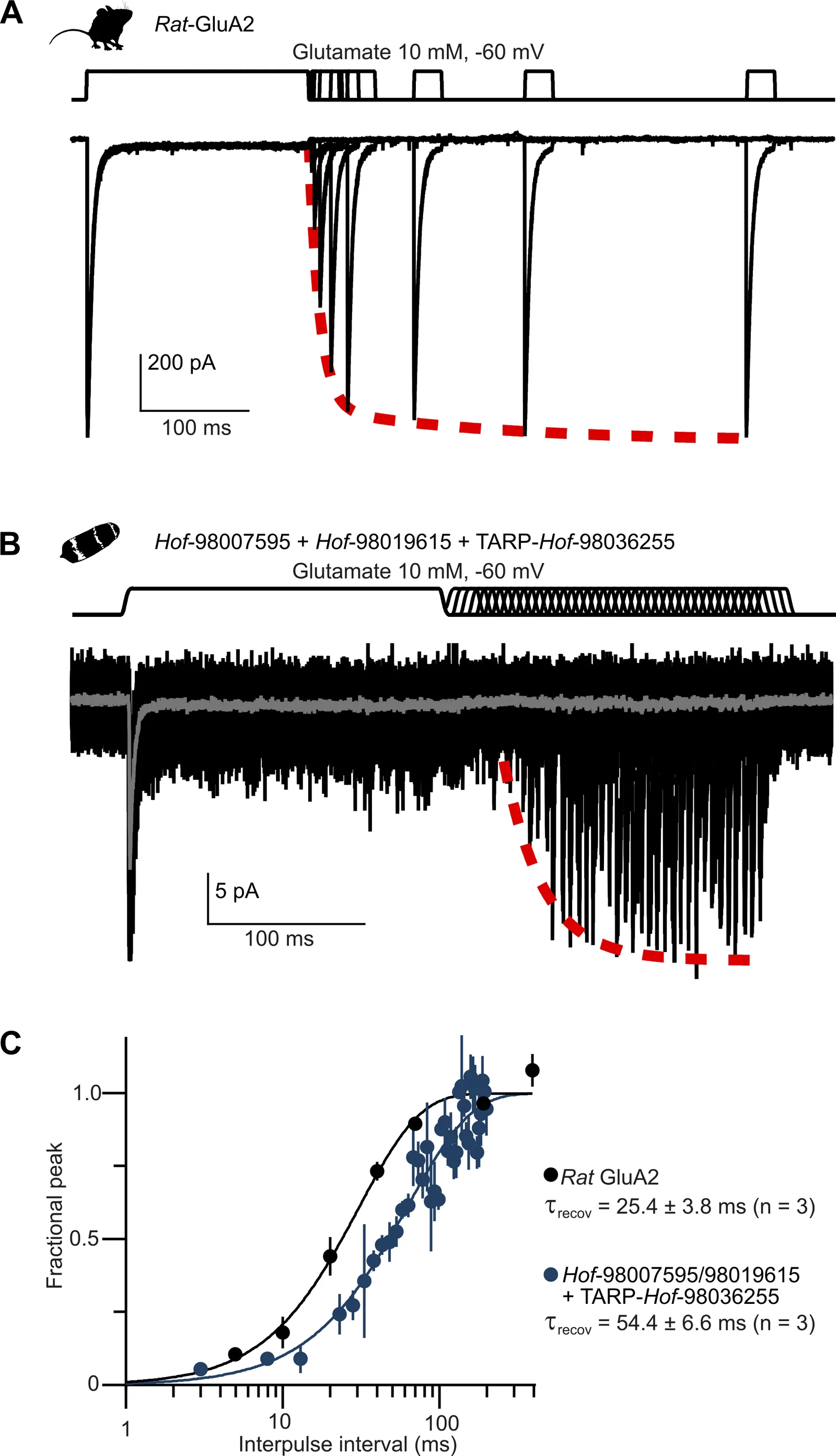
**Rapid recovery from desensitization in diverse AMPARs. (A)** Representative outside-out patch-clamp recording from HEK293 cells expressing *Rat*-GluA2 (flip, Q) AMPARs. Recovery from desensitization was measured using a paired-pulse protocol consisting of an initial 200 ms application of glutamate (10 mM−60 mV), followed by a second glutamate pulse delivered after increasing interpulse intervals (intervals from 5 to 800 ms). The fit is in red. Upper traces show the piezo command voltage. **(B)** Same as in A, but for *Hof*-98007595/98019615 heteromeric AMPARs co-expressed with *Hof*-TARP-98036255, interpulse intervals increasing in 5 ms increments, and averaged currents overlaid in gray. **(C)** Fractional recovery plot (mean ± SD, *n* = 3), fit with a Hodgkin–Huxley-type function, giving indicated time constants for recovery (τ_recov_).

Recovery from desensitization was also rapid in *Hof*-98007595/98019615 heterotetramers (+*Hof*-TARP-98036255), with a recovery time constant of 54.4 ± 6.6 ms (*n* = 3), although this was approximately twofold slower than that for WT rat GluA2 (flip, Q isoform) receptors without stargazin (25.4 ± 3.8 ms (*n* = 3); P = 0.047; Fig. 8, A–C). In the presence of stargazin, recovery is 10–20 ms for rat GluA2 (Carbone and Plested, 2016) and 90 ms for GluA1 homomers (Priel et al., 2005).

Interestingly, despite *Hof*-TARP-98036255 resembling type I TARPs through an increase in macroscopic current amplitude and a tenuous phylogenetic relationship, its faster entry into and slower recovery from desensitization more closely aligns with properties characteristic of type II TARPs—such as the approximately twofold slower recovery seen with γ−5 relative to WT, TARP-less GluA2 (Gangwar et al., 2023). However, given that we cannot be certain whether the final receptor in HEK cells assembles with *Hof*-TARP-98036255, we cannot unambiguously attribute the determined recovery time to TARP-ed *Hofstenia* channels. Either way, recovery of *Hof* channels from desensitization still qualifies as fast and more comparable with mammalian AMPA receptors than with kainate (recovery time for K2 homotetramers ∼ 2 s [Carbone and Plested, 2012]) or NMDA receptors (GluN1/2A ∼600 ms and GluN1/2B 1–2 s [Vicini et al., 1998]). Thus, despite numerous duplications occurring independently in diverse bilaterians, extant AMPARs have retained the activation kinetics that make them suitable for brief postsynaptic depolarizations in response to synaptic transmission.

## Discussion

### A blueprint for fast glutamate receptors for bilaterians

Our data show that throughout Bilateria, AMPARs are glutamate-selective receptors converting brief neurotransmitter signals into depolarizing currents. This contrasts with other branches of the iGluR tree, such as NMDA, ε, and Δ, in which closely related homologues respond to different ligands and/or show different kinetics (Alberstein et al., 2015; Bossi et al., 2023; Rosano et al., 2024; Singh et al., 2025). Whether or how it contrasts with other AKDF receptors from invertebrates awaits characterization of those receptors. On a molecular level, this palette of AMPAR sequences with similar function should help dissect the molecular determinants of function, such as ligand selectivity and entry into desensitization, which are either dispersed throughout the receptor or unidentified (Mosbacher et al., 1994; Carbone and Plested, 2012; Alsaloum et al., 2016; Aittoniemi et al., 2023; Vinnakota et al., 2025). On a biological level, the biophysical function of diverse AMPAR homologues may tell us something about the evolution of bilaterian nervous systems.

Bilateria and Cnidaria inherited from their last common ancestor a primitive nervous system. This included neurons with homologues of all the pre- and post-synaptic machinery we are familiar with, including an ancestral AKDF receptor among other iGluRs (Stroebel and Paoletti, 2021; Colgren and Burkhardt, 2025), and probably took the form of a dispersed nerve net with numerous bidirectional chemical synapses (Bosch et al., 2017). In an early bilaterian, several duplications of primitive AKDF gene(s) led to ancestors of closely related AMPARs and KRs, ancestral Δ iGluRs, and several other subfamilies not found in bilaterians (Moroz et al., 2021; Rosano et al., 2024). The nature of these non-AMPAR/KR AKDF iGluRs remains mysterious, as Δ iGluRs are often GABA-gated or relatively inactive (Piot et al., 2023; Rosano et al., 2024), an AKDF iGluR from a placozoan (a simple, film-like non-bilaterian) is constitutively active (Seljeset et al., 2024), and few others have been experimentally characterized. A duplication of the last common ancestor of AMPARs/KRs then gave rise to an ancestral AMPAR and an ancestral KR. The functional redundancy and thus relaxation of selection pressure on these new, very similar genes may have allowed rapid neofunctionalization in one of them (Conant and Wolfe, 2008). Our results show that early-branching xenacoelomorph AMPARs share fast kinetics with mammalian AMPARs and that Ca^2+^-resistant pore properties arose several times independently in AMPARs (below). Thus, the ancestral AMPAR probably conducted inwardly rectifying currents that were larger in the presence of extracellular Ca^2+^ and then quickly evolved the fast deactivation kinetics that set it apart. It is possible that AMPARs already became heteromeric in stem bilaterians, as our phylogenetic analysis suggests xenacoelomorphs retained two early-arising AMPAR genes (clades 1 and 2 in Fig. 1 C), one of which was lost by nephrozoans. However, we acknowledge the alternative possibility that early bilaterians had only one AMPAR gene, and an AMPAR gene duplication in stem Xenacoelomorpha gave rise to xenacoelomorph clade 1 and 2 genes, with clade 2 branching basally in our tree only due to its greater divergence.

Had a complete glutamatergic synapse containing AMPARs already evolved in the earliest bilaterian(s), before the independent centralization of neuropils into brains and nerve cords (Martín-Durán et al., 2018)? This will require future immunohistochemical studies localizing AMPARs subcellularly. But it could be that during these later, independent stages of CNS evolution, AMPAR genes began to duplicate and yield the unique complement of AMPAR genes in each lineage.

It is notable that AMPAR gene number is lower in ecdysozoan protostomes than in most other bilaterians. For instance, AMPARs are absent from some crustaceans, where NMDA receptors and kainate receptors have instead diversified (Northcutt et al., 2016). AMPARs are present in the *D. melanogaster* brain but are notably absent from the mushroom body, where associative learning is centered and where acetylcholine is employed instead (Daniels et al., 2008; Deng et al., 2019). Curiously, excitatory acid-sensing ion channels that depolarize postsynaptic neurons together with AMPARs in the mouse brain have also been lost in ecdysozoans (Du et al., 2014; Martí-Solans et al., 2023), and we therefore think that central synapses and mediators of synaptic plasticity have evolved differently in ecdysozoans compared with other bilaterians.

### Convergence on heteromeric architecture

When xenacoelomorphs, spiralian protostomes, and deuterostomes emerged, their AMPAR genes duplicated. Why, in each case, were the duplicate genes retained? The likely initial mix of 25% “Gene A” homotetramers, 25% “Gene B” homotetramers, and 50% “A/B” heterotetramers may have given way to mostly A/B heterotetramers after chance mutations in the N-terminal domain made intersubunit oligomerization kinetically favorable (Hansen et al., 2021; Cisneros et al., 2024). This may have been accompanied or followed by additional mutations elsewhere in the protein. Mutations that decreased interactions with different chaperones may have created further co-dependence for expression (Diss et al., 2017). Alternatively, the retention or enhancement of proteomic or genetic interactions with various chaperones, functional partners, or transcription factors may have added functional advantages and led to expression in different cell types (Veron et al., 2007; Marques et al., 2008), especially in the case of distinct heteromers as is the case in *H. miamia*. Consistent with this, in *H. miamia*, all AMPAR-expressing cells express the obligatory subunit *Hof*-98007595, whereas the optional partner subunit, *Hof*-98014323, or *Hof*-98019615 together with TARP *Hof*-98036255, are only expressed in different subsets of those cell types (Fig. 5 F; Hulett et al., 2023). Such single-cell RNA-sequencing data are not available for the four *L. anatina* AMPAR genes, but the two that formed receptors in our hands are expressed at largely similar developmental time points according to the prevalence of RNA transcripts in bulk sequencing data (Fig. S3, C and D).

Combined obligate heteromerization and sub-functionalization seem widespread. When one subunit becomes more important, e.g., glutamate-gated currents—GluA2 in rat, *Lin*-g8707 in *Lingula*, and *Hof*-98019615 or *Hof*-98014323 in *Hofstenia*—then the genes coevolve and presumably accumulate mutations at interfaces that make self-assembly less likely, as suggested by previous studies on other oligomers (Duguet et al., 2016; Cortez-Romero et al., 2025). Fine-tuning of AMPAR functional properties seems to have occurred via different mechanisms in AMPARs of different animals (below).

Regarding iGluR activation mechanisms, it will be interesting to establish in the future whether the subunit type that seems more dominant in gating occupies the B and D positions, as is the case for *Rat*-GluA2 (Herguedas et al., 2019; Zhao et al., 2019). Also deserving of testing is the 2:2 stoichiometry that is easy to assume for diverse heterotetramers. A 1:3 stoichiometry is also foreseeable, based on observations of di-heterotetrameric insect olfactory receptors (Zhao et al., 2024). The recurrent emergence of obligate heteromerization, and the previous finding that each of the four rat AMPAR subunit NTDs binds with 10- to 100-fold higher affinity to the other three subunit NTDs than to its own (Zhao et al., 2017), lead us to predict that somewhere in the mammalian lineage, there are, e.g., GluA1 and GluA2 subunits that only hetero-oligomerize and can no longer form homotetramers.

### Convergence on a set of receptor complexes with distinct biophysical properties

Mammalian AMPARs’ “choice” of different subunits leads to significant functional diversity. For example, approximately threefold faster kinetics of GluA4 subunits confer auditory synapses with the speed required for temporally precise sound localization (Borst and Soria van Hoeve, 2012; Hansen et al., 2021). Highly expressed GluA2 subunits confer on AMPARs in most principal neurons of the mammalian brain relatively low Ca^2+^ permeability and a linear I-V relationship (Sommer et al., 1991; Swanson et al., 1997). In contrast, GluA2-lacking AMPARs and GluA2-containing AMPARs with auxiliary proteins that modulate channel activity are prevalent in several cell types and even different subcellular compartments, and these AMPARs conduct larger currents at negative potentials and carry substantial Ca^2+^, implicated in both depolarization and metabolic changes (Liu and Zukin, 2007; Diering and Huganir, 2018; Cull-Candy and Farrant, 2021). This is via an RNA editing mechanism that arose in vertebrates, where adenosine deaminases convert adenosine to inosine in ∼99% of GluA2 pre-mRNAs, essentially converting a pore-lining, neutral glutamine residue at the Q1 position into a positively charged arginine that repels Ca^2+^ ions and prevents block by intracellular polyamines (Seeburg et al., 1998; Kung et al., 2001).

We show that acoels, since diverging from other xenacoelomorphs, have evolved a similar choice of AMPA receptors: those with voltage-dependent currents that are larger in the presence of extracellular Ca^2+^, or those that are voltage-independent and insensitive to extracellular Ca^2+^. Remarkably, this is also via an arginine residue in the channel pore, but in contrast to mammalian receptors, this is (1) encoded in the chromosomal DNA and (2) at the “Q2” position in the channel pore. Single-cell RNA sequencing data indicate that a prominent population of neurons expresses the Ca^2+^-sensitive isoform, together with a TARP that leads to larger currents, whereas three other populations of cells express the Ca^2+^-insensitive isoform (Fig. 5 F; Hulett et al., 2023). Interestingly, in both rat GluA1/GluA2 and acoel 98007595/98014323 receptors, it is the subunit that evolved Ca^2+^ resistance that also seems more important for channel activation by glutamate according to mutagenesis in the LBD. A chromosomally encoded arginine at the Q1 position has also been reported in certain early-branching vertebrates and fishes, raising the possibility that such a mechanism has evolved several times (Kung et al., 2001).

What underlies the inward rectification and larger currents in the presence of extracellular Ca^2+^ in “Q1Q2-containing” receptors from throughout the AMPAR branch? The greater amplitude of currents at negative potentials in the presence of extracellular Ca^2+^ was decreased in three ways: by removing extracellular Ca^2+^; by mutating to an R1Q2 motif, as occurs in mature rat GluA2 subunits (above); or by the natural occurrence of a Q1R2 motif in certain acoel AMPARs. By homology with rat AMPARs, we think these effects are likely due to intracellular polyamine block at positive potentials, higher single channel conductance, and higher Ca^2+^ permeability in Q1Q2 receptors (Cull-Candy and Farrant, 2021; Heiser et al., 2025), especially in oocytes, where Ca^2+^ influx is likely to activate Ca^2+^-activated Cl^-^ channels that carry inward current at negative potentials (Miledi and Parker, 1984; Li et al., 2016). However, our current data cannot distinguish between Ca^2+^ permeation, polyamine block, or potentially some other voltage-dependent modulation by Ca^2+^.

We also note that the number of functionally distinct AMPAR subunits in vertebrates is essentially doubled via an alternative mechanism to gene duplication. Mutually exclusive “flip” and “flop” exons, encoding homologous but slightly divergent versions of a short segment at the rear of the LBD, arose in the AMPAR gene of the very earliest vertebrates (Chen et al., 2006). This gave rise to alternatively spliced subunits that differ in both their speed of desensitization and their modulation by TARPs, and the two exons have been retained in GluA1–4 genes of most vertebrates (Chang et al., 1998; Chen et al., 2006; Bowie, 2026). Alternative splicing is far more prevalent in vertebrates than invertebrates (Kim et al., 2007; Huang et al., 2016), but alternative splicing in the species we have studied, and its effect on AMPARs, remains to be studied in detail.

Another means of AMPAR complexity is via co-expression with TARPs and other auxiliary subunits, which affect AMPAR cell surface expression, glutamate potency, and even ion permeation (Jackson and Nicoll, 2011; Schwenk et al., 2012; Zhao et al., 2019; Miguez-Cabello et al., 2025). We show that AMPARs from diverse bilaterians are modulated by cognate TARPs, with dramatic increases in current amplitude in most cases, although we have not dissected whether this is due to increased cell surface expression and/or changes in channel gating imparted by the TARPs. Our experiments with outside-out patches show that acoel AMPARs, which are built of AMPAR subunits and TARPs that co-express in *H. miamia* neurons, activate and deactivate with similar speed to rat AMPARs, suggesting that diverse bilaterian AMPAR complexes are suitable for brief depolarization in response to synaptic glutamate. However, deactivation and desensitization appear to occur at similar speeds in the *Hofstenia* AMPAR complex we tested, suggesting that at continuous, high-frequency firing, postsynaptic currents may diminish in amplitude.

### Limitations and outlook

Multiple studies now agree that AMPARs first emerged in an early bilaterian, and our and others’ data suggest that numerous independent duplications in specific lineages gave rise to the complement of two-to-six AMPAR genes in most extant bilaterians (Ramos-Vicente et al., 2018). However, the number of AMPAR genes in the ancestral bilaterian, when the first duplications occurred, and how many AMPAR genes were lost in some animal lineages, depends on different datasets, tree topologies, and interpretation. For instance, a nephrozoan-only analysis suggests a single AMPAR gene in an ancestral bilaterian (Ramos-Vicente et al., 2018), whereas our use of numerous xenacoelomorphs implies at least two AMPAR genes in the ancestral bilaterian, one of which was lost in an early nephrozoan after the split with xenacoelomorphs. Furthermore, branch support for the deuterostome AMPAR branch is relatively low in both studies, raising the possibility that future analyses with different deuterostomes might collapse this branch, which would point toward multiple deuterostome AMPAR clades and thus several more gene losses during evolution. Such dataset-dependent changes in certain branches of trees are common (Moroz et al., 2021; Steenwyk et al., 2023), and some AMPAR duplications and losses will therefore remain difficult to pinpoint. However, certain cases of gene duplication appear more robust, including the gene duplication in acoels that gave rise to *Hof*-98019615 and *Hof*-98014323; and several duplications in an early vertebrate, which gave rise to the four genes that mammals have inherited. TARP relationships are even harder to dissect, with more noticeably different topologies across datasets and with generally low branch support (Figs. S6 and S7) (Ramos-Vicente and Bayes, 2020).

Despite the caveat that our experiments involved invertebrate genes expressed in vertebrate cell lines, our results point toward obligate heterotetramerization among numerous diverse AMPARs. This is supported by our finding that functional channels require co-expression of different subunits in cells from both amphibians and mammals and by correlations between (1) the genes that form channels together in our experiments and (2) the transcripts that express in the same cell types, tissues, or life stages in acoels and brachiopods (Luo et al., 2018; Hulett et al., 2023), but admittedly, obligate heteromerization *in vivo* remains to be proven.

Studying such numerous, diverse AMPAR genes was made possible by expression of these genes in *Xenopus* oocytes, as expression in HEK cells was much less successful. This restricted many of our experiments to qualitative observations because of slow solution exchange and no control of intracellular ions in oocytes. But HEK cell expression of an AMPAR from the earliest branching bilaterian lineage of Xenacoelomorpha allowed us to show that fast kinetics are shared by deuterostome and xenacoelomorph AMPARs. Nonetheless, our combined phylogenetic and experimental treatment of diverse AMPARs shows that the hallmark of the subfamily is fast responses to glutamate with tunable biophysical properties via the incorporation of different novel subunits, involving substantial convergent evolution, for example, in sub-functionalization of subunits in channel gating and in the emergence of AMPARs with different pore properties.

By offering a more diverse palette of AMPARs and highlighting key functional differences, our study paves the way for studies dissecting structure-function relationships in iGluRs in more detail. For example, chimeric constructs swapping vertebrate and invertebrate AMPAR sequences might identify segments that determine responses to pharmacological modulators, and ancestral reconstruction combined with mutagenesis could identify residues involved in obligate heteromerization and sub-functionalization. Our results also suggest caution in physiological experiments dissecting invertebrate synaptic function, as many invertebrate AMPARs respond to classical pharmacological modulators with opposite responses to mammalian AMPARs.

## Supplementary Material

Table S1 shows synthetic AMPAR genes used for expression in *X. laevis* oocytes.

Table S2 shows synthetic TARP genes used for expression in *X. laevis* oocytes.

Table S3 shows synthetic AMPAR genes used for expression in HEK293 cells.

Table S4 shows synthetic TARP genes used for expression in HEK293 cells.

Table S5 shows summary of outside-out patches.

Data S1 shows manually trimmed iGluR amino acid sequence alignment (FASTA format).

Data S2 shows iGluR gene tree (NEXUS format) based on trimmed alignment.

Data S3 shows untrimmed iGluR amino acid sequence alignment (FASTA format).

Data S4 shows iGluR gene tree (NEXUS format) based on untrimmed alignment.

Data S5 shows manually trimmed TARP amino acid sequence alignment .

Data S6 shows TARP gene tree based on trimmed alignment.

Data S7 shows untrimmed TARP amino acid sequence alignment.

Data S8 shows TARP gene tree based on untrimmed alignment.

## Data Availability

The data that support the findings of this study are available from the corresponding author upon reasonable request.
