# Supplementary material for: Tunable AMPA receptor function via recurrent evolution of heterotetramers
Source: J Gen Physiol. 2026 Aug 27;158(6):e202614039. doi: 10.1085/jgp.202614039 (PMC13520880; doi:10.1085/jgp.202614039)
Supplement: Supplementary file 1 [file jgp_202614039_tables1.docx]

**Table S1. Synthetic AMPAR genes used for expression in *Xenopus laevis* oocytes**

Blue, restriction sites; pink, spacer and/or Kozak consensus-like sequence.

*Saccoglossus kowalevskii* GluA

GTCGACACCATGGGACCGTGCGTGCAATTGTTTATCATACTGGCCAACTGCTTAGTGTTGTTCGTTGCCTCCATACAGTCTGCAGCTAGTATACAAATCGGAGGGATGTATAACGCTCATCCGTCACAAGAACAGAGCGCATTTCGCCATGCTCTTTTCCAGAACAATATACACAGCAACACATCCATTCAGCCGCTTAACTTAATGGCGCACAACGAAAACCTTGATCATTCAGACAGTTATGTGCTGGCCGATAAATTCTGTACACTGATTCGGCGTCAAGTATATTTATTAGTAGGTGTTACAAGCATGTCCTCGTTACCATCCATCAAGTCATACAGCAATACATTTCACATTCCATATATTACACCAAGCCTTTCCGGAACAGAAATGGGTACAAAATCGTCAGACTATTTGATTTACATGCGCCCGTCGCCAATACCAGTGTTAGCTGGGCTTATTAAGTACCACAAGTGGGATAAATTCAACTATTTATATGATGACACAGAAGGGTTGTATCGACTACAATTGTTACTAGAAGTTGCAGCTGACAGATATTTAGACGTGAGGCCTTATCACTTACGCGGTGTTACCAGTGTTATTAACATATTAAACAAAATACGAGTCTCTGGTATAACAAAAACTATTTTAGATACGTCTCCGCCAAGTAGAACGTCGGCAATACTCACTCAAGCTCTGCAGCTTGGCATGGTCACAAGGAAGTATCATTTTGTAGTTCTTGACTTTGACGTGTACCATATCGATCTTGAAAATTTTAAATACGGAGGCGCGAACGTGACTGGTTTTCGTATGGTAGACGAAACGAATACGAGGCTAAAAGAATATCGAGATGTCTGGAACCAATTCACTGAAAAATATGCAGAATTTGAAAATATCACATACGTGAAATTCCAGGCGGCGTTGGTTTACGATGCCGTATCGGTGATCACCGAAGCGTTTCGACTGATGAAGAATGACCACGTGGACATAGACTCAGAACTAATAAATTGCGACAACAAACATCAAAAACCTTGGCGTGATGGCGAAAAAATGAATAACTACCTAAAAGAGGTCGATATCCAGGGACTGACTGGGCCAATCAGATTTGACAAATTGGGAAGGCGGACTAATTATACCATGGATATATTGAGCCTTGTTCCAGACGAGACGGGAAAAGCTAGGTTTGATAAGGTGGGTAAGTGGGATTCGTCCGGTGAAGGTCACATTGGTATTGGTAGTGATGGAGAGGTACTAAGTTTCCCAGCTGACAGCTTCAACAGAACCCTTCGTGTCACTACAATTGACGACGATCCATATTTTATGCTTAAGAAAAATGCAGATGAGTTCGAAGGAATGGACAAATACGAAGGATACTGTGTAGACTTGATGGCAAAGATTGCCGACGTCCTGAAAATTAAATATGAATGGCAAATTGTGGAAGACGGTAACTACGGTCGTCTCAACGAAGACACGAATAGATGGGATGGAATGATAGGAGAACTCATAGAAAACAAAGCGGATTTAGTTGTGGCACCTTTGACTATCACTTCTGACCGTGAACCTTATGTAGAGTTCACAACACCATTCATGGGTTTGGGTATCAGTATCATGATTAAGAAACCGCAAAATACCAAACCAGGCGTCTTCTCGTTTCTACACCCTTTATCTCATGAGATCTGGATGTGTATCGTGTTTGCCTATGTTGGTGTTAGCGTGGTGCTCTTCTTGGTTAGCCGTTTCAGTCCCTATGAGTGGCACGCAGTTGATGCTCCGCCGCCATTGTTTGACGACACTGGGGATAGGCAGGAGATGGCCAACGATTTTGGAATCTTTAACAGTCTTTGGTTTTCACTGGGTGCATTTATGCAGCAAGGATGTGATATTTCTCCAAGATCGCTATCAGGTCGTATAGTTGGAGGTGTGTGGTGGTTTTTTACTCTAATCATCATATCTTCCTACACTGCCAACCTGGCTGCTTTTCTTACCGTTGAGCGGATGGTGTCGCCAATAGAGTCAGCTGATGACTTAGCCACACAGACTGAGATATCCTATGGCACGTTAACGTCTGGGTCTACAACGTCTTTCTTTGAGAAATCTACTGTACCAACGTACACACGAATGTGGGCCTATATGAGCACTACACAGCCATCGGTCTTCACAAATTCCATAAAAGAAGGTATTGAAAGGGTTAGGCGCCAAAAAGGAAGATATGCTTTCCTATTGGAGTCAACGATGAATGAATACATCAACCAACGTAAACCATGTGATACAATAAAGGTTGGGAACAATCTGAACTCAAGAGGTTATGGTATAGCTACAAGGAAGGGATCTGCACTCAGAGAAAATTTAACCTTGGCAATATTAACGCTGAGGGAGAGAGGCGAACTACACAAAATGGAGAAGAAATGGTGGTATGAAAAAGGTGAATGCGGTGACGGGGATTCCAAGTCACAGGAAACAGCCAATGCACTGAGTCTCAGTAATGTAGCAGGTGTATTTTATATATTAGTGGGTGGTCTGGGATTGGCAATGGTGGTTGCGCTAATGGAATTTTGTTATAAGTCAAGGAAGGAAGCGAGACGAGCCAAGACTAGCCTATCAATCGCAATGAGGGCAAAGGCCAGAATGTCTATAAAAGGTGACAATGGGAGAGTTTCTACACCAGATTTCACAAAGTCTATATCTACTGTCCCAATTACAAGGTCGTCGAACGCAAATTCTGGAAACATGTTCAGTAGGAAGAAAAGTTTAACGCCATGCCCAAGTGAAGAAGATTTACTGGCTAAAGTCAAGAAAGAAGAAGTGTACCTGAGAGAAAATGTCAAAGTAAAATAAGGATCC

*Lingula anatina* GluA-g8706 (codon-optimized for *Xenopus laevis*)

GTCGACGCTGCCACCATGGTCTCTCGTTACTTTGCTTTGACACTGCTATTCACCGCGGATATATGCATGACCAGGATTCAGCGTGATATTCCAGTAGGACTTCTGCTCGAATCTAAAAATGTAGAGATTTACCAAGCATACCAATTTAAGTTCGATGAAGAGAATCGAAGAGCAAAGGAAAGCAAGGTCAATTCCGATGATGGACATAAGTTCACCCTTAACAGGATAGCTGAAGATTATATTGATATATCCAGCAACTATGACTACAATCATACATTCTGTAATCAGATGGCTGCTGGAGTCGCAGTTATATTTGGACTGTACTCTACATCCACGGCCGATTTCGTCACCTCCAGTACCTCCATGTATCACATGCCCTTCATCTCAACCTCTCCCCCAGAGCTGTTCCGTCCCCCGTCTGAGTTCATGATCCACCTCCAGCCTCGATACGAAGACGCCTTGGCTGATTATATGATCCGTAAGGAGTGGAAAGAAGCTATCTATATGTACAGAACAAACGAGGCTCTTGCTCGACTTTACCACATCATAGGGGCTATCAACCGTGGGCACGCTTACATCGTCGTCATCTTCAAGCATGTCACAGATGTGTTGGATGCTCTGGCTGCTCTGGCGCAATACGACAAAGACTATCCGCACTTGGATACTACCAAGAATATCATCATCGATCTGTCAGTTGACGAGACCAACACTCTCTTAACCCGAAGGTTTAAGGAAGTTCCTCAGTATCGTCTAAAGTACAATTACCTGATCGCGGGATTGCAAATAAACGAGCTTGACTTTTCGGAATTTCCAAATGGTGGCATTAATATCACCGGATTCCACCTCACAGAATCCCATTCACCTAAGATGGAGAAAGTTCTTAATGAATGGAAAGGACGATTCGTTTCCAGTCGTAAATCCCTGATTTCTCATCAGCTGGCTCTAGCAATGGACGCTGTGGACGTCTTCACCCAAGCTATGCACAACCTCACAGAACAGCAAGAGAAGATGTTTAATAATGGATCATTCCGTCTGTCATGCGCTCTATGGCCTTCTAAGCCAACACCGTGGCCTCTAGGGAAGATGATCACACAGGCTATCCGTCGTGTTGGACTGCATGGCCTGGAAGGACTGACCGGAAAGATTGTGTTTGATGACCGAGGTTTTCGAACAAACTTCACCCTGCAACTGACGTCTACGACTATAGATCATGGACTTGCGAAAGTAGGAACGTGGACCCCGAATCCCAGAGCTAACCGACCACGCTTGTCGTTCTTTGAAAAGCCAAAAGACAGCTCAGGGTTGCGAGATAATACCGTCCGCAATAAGACGTATCTGATCACGTCCATAGAAAGCGCCCCTTTCATGATGGTCCGTACCGACCCGAAACCGGGCGGCGGCAATTGGACCGGAAACGACCGCTACGTAGGATTTTGTAAGGACATTGCTGATAAACTAGCTCAGAGCCTGGGAATCAACTACACCATCAACCTGGTCGGAGACAAGAATTACGGCGGTTACGACCCGGAGAATCACACCCATCCCTGGGATGGAATGGTCGCTGAACTGATCAATGGGGTAGCTGATCTGGCCATCGCTGATCTCTCCATTACTCTGGATCGAGAGAAGTTCATCGACTTCTCAAAGCCCTTCATGAACCTGGGAATCTCCATCATGATCAAAAAACCTGAGGAAATCGTTCCGAATATTTTCTCTTTTATGGACCCTCTATCTGATAAGATCTGGATGTGCATCACCTTTGCCTATCTGGCGGTATCCGTTGTCCTCTTTCTCGTGACCCGGTTCTCCGCTAGTGAGTGGGTAGTGGAGGAAAATGAGGAGGACAACGGAGACCCCGAAGTCCACAATGATTTCAATTTCATCAACAGTCTGTGGTTCTCACTGGCAGCCTTTATGCAGCAGGGCTGCGAACTGTGCCCAAAGTCCATTTCCGGTCGCATCGTCGGCTCTGTCTGGTGGTTCTTTACCCTGATCATCATCTCCTCCTACACCGCCAACTTAGCAGCTTTCCTGACCGTCTCCAGGATGAATAGCCCTATCCAGTCAGCAGACGACCTCGCCAAACAGACCGATATTGCATACGGATGCATTGTCTCCGGAAGCACAAAGAAGTTCTTCCAGGATTCTAAAATTTCAACCTTCGAACGTATGTGGGCTTACATGTCCTCTGCGGACAACAACGTCTTCGTGAAGTCCAACAAGGAAGGTGTGGAGAAAGTTAGGAAGTCCAATGGAAAGTACGCATACCTACTAGAGTCCACTGTCAACGAGTACCTTAACAACAGATACCCTTGTGATACGATGAAAGTCGGTAGTAACCTGGACTCCAAAGGTTATGGTGTCGGAATGCCTAAGGGATCTCCACTGCGTGATTCCGTGACTCTTGCAGTTCTGGAGCTTCTTGAGAATGGTGAGATTGAGAAGAGGCGACGTTTTTGGTGGGAGGAGTCGGAGGGAGGAGGGGAAAAGCTGTGTCCCAAGGAAAACTCTGATAAAAATGATCCAGCATCGAGTGCTGCGCTGTCCATCGTCAAGATTGCTGGAATATTTTTAATCCTCCTTGGTGGAATGGGTCTGGCCTTAGTTCTGAGCATCATGGAGCTTTTCTACCGTACCAAACTTGATGCTATGTCTAATAATATTACATTTTCCGATGAGATGAAGACTAAAGCACAACTTTTAGTCCGAGGAAGACTTGAAAAGGAACCAGCCACGGATGCATCCCCAGTTCATAAGTCTGTCTCCACATACACATATTCCACCCCATCGGCACAACTGATCACTCTGGATGGTTACTCAGATGCTGGAAATACTCACACCCAAGTTTGAGGATCC

*Lingula anatina* GluA-g8707 (codon-optimized for *Xenopus laevis*)

GTCGACGCTGCCACCATGAATCCTGCTGCTTGGATCTTTGTGGTATCCGTATACAGCGGCGGAGTCGTCTTTGGGAAGATACTGGAGCATTCTGTCGGACTCATTCTATCTAACACTGCTCCTAGACACGGAGATATAGAACTGGCGTATCGTTACGCATATCTGGGAACCCTCTGTCGCCACAATGCTCAGGGATTCATTGAAGGACGTAATTATACAAACTATAGGTTCAAATTCTCTTATACCCATTCCCTAAATTTCATAAATATTGGTAACAACTTCGATTATAATTACACCATCTGTAACACACTGCAGTGGAAGGTTTTCGCCATATTTGGCATCTACGACACTTTCACCGCCGATTTTGTGGCTTCAACGACCGGTCTGTATCACATCCCCTATATCACGCCCACCTCTCCCGAGATCTCCTACAGACCACATGGTGATTACATGATTCATATGCAGCCTCGTTACGCGCAGGCGCTGGTGGACTACATTCTTCTGAGACGCTGGAAATCGGTCACCTACATCTACGATTCAAATAAAGAAGGACTGGCTCGTATGTACCACGTGGTCGGCGCAATAAATAATGCCGGAGCCTATGTAGTCATCCATTTCAAGTACGTTACTAACGTCACCGACGCTTTGGCCAAGCTGGCTGCCATGGATAAGACAGGATCTGAAAAGAAACATAATGTCATAATTGATATGTCCCTAAACGACACCAACACCGTACTGAAACGAAGATTTACAGAAACCCCTCAGACCAAACTGCTGTATAATTACTTGATAGCAAGATTGTTTACAAACGAGATCGATTTCAAGGCTTTCCCTGCTGGAGGAATGAACATCACTGGTTTCCACATGGTGGATTACGATACGGATGTCACCGTAAAAAAATATAATCAGGTCTACAGGAGATGCGACCTGAAGTGCGGATCTTTTGACAACACCTGTCCTACGTCAAACGGTGCTGCTTTTGCGGCCGACTCTCTGACAGTTTTCATCAAAGCCATGGACTTGCTGCATAAAAACGGTTCTTCCAACTTCCCTACCAACAACCCGTATCCCAAGAACTCTGGCGGCCTAGCTTGTGCAGAGTGGCCCAATGCCCCAAAACCTTGGATATACGGGGAGAAATTGATTGGCGCTTTGAAAAAGGTCGAACTGGACGGATTGACAGGAAAGATATTGTTTGATGAAGATGGATACCGAATAAACTTCACCCTTTATCTTTTCGGTACAACGTCTCAGGAGGGTCCTGCAAAGTTGGGAACTTGGGAGTCCCATCCGCGTCGCCTGAATCGACCACGTCTTAATCTTATACACGATACCAAATCAGTCGGGCTGCTAAATGTAGATCCTGATAACAAAACTTACATTGTTACAACTATATTGAGAGACCCTTACATGATGGAGAAGATTCACCCTCGTCCAGATGGAGGTAATTGGACGGGTAACGATCGTTACGAAGGATTCTGCAAAGACATCATCGACCAGCTGATGCGTGACTTAAATATGAATTATGAGTTACGTGTGGTGGCCGACGGATCACACGGGAGCAAACAAAAGGGAAACGTGACACACCCATGGAACGGGATGGTGTGGGAGCTGATGACAGAGCGAGCTGATATGGCTATCGGAGATCTGACCATCTCCGCTGAACGCGAAAAGGTGATCGATTTCACCAAGCCTTTCATGAATCTTGGCATAAGCATTATGATCCGAAAAACACCCGGAAGTCAGTTCGATATTACCGATATGTTCATATTCATGGTGCCTCTATCCAAGGAAATATGGATGTGCATTGCTTTCGCCTACCTGGGAGTCTCCGTTGTCTTGTTCCTGGTGAGCCGGTTCTCCCCCGCAGAATGGTTGATCAACGATCCAATGGAAGATGACGGTAAGAAGAAAACGTGCGTCAGGAATGACTTCGGCATTTACAACTCCTTGTGGTTCGCATTGGCTGCTTTCATGCAGCAAGGATGCGAAATTTGTCCGAGGTCCGTCTCAGGACGCATTGTGGGATCTGTTTGGTGGTTCTTCACCCTGATCGTCATCTCCTCCTACACGGCTAATCTAGCTGCTTTTCTTACCACGTCCAGAATGATCTCACCCATCGGTTCAGCGGAAGATCTTGCAGCACAGACAGAGATCTCTTATGCGACAAGTCGGGAGGGAACATCCAGAGATTTCTTCAGGGAATCTAAAATCTCTACATACGAGCGTATGTGGGCGTACATGTCTTCCCGGCCACACAACTTCCCCAAGAATAACGCGGAAGGGGTCCGTAGGGTCCGTGACGAAAACGACAAATTTGCTTTTCTCATGGAATCTACCTCCATTGACTATCACAGTAACCGTGAGGACTGTACCACCACCAGAGTTGGAGATCTGCTGAACTCCAGATCCTACGGTATCGGAATGCCCAAAGGATTTAGTAAAAAGAACGTCGTCACCCTGCAGCTGCTAACCCTAGTCGAATCCGCATTCGTTGAGGAGCGTCGAAAATACTGGTGGGAAGAGGACTCCAGTAGAAGAGGACTTTGTAAGGATCGTCAGGATACCAATAAAAAGGGTGAGGATACCAGTTCGTCTCTTTCTATTTCTAAAATCGCAGGTATATTCATCATTCTGCTCTGTGGGATGATGATGGGAATAATTATTTCTATCCTGGAGTTATTCTATAAGACCAAGATTGACGCTTTCAGAAAGCACATAACGTTTTCGGAGGAATTTCAGTCCAACGCACAGCTGCTTATTAAGGGAAAGATGCCTCTTAAGGACGGCGAGGACGCTTCCGAGGATGTGCTTCCAGGTGATACGGCGGTGAAGTGCACCACCACGACCCCCTTTGCCCAGCTGATCACCCTGGATGGATACTCAGATAACACACAAACTGAGGTATAAGGATCC

*Lingula anatina* GluA-g16612 (codon-optimized for *Xenopus laevis*)

GTCGACGCTGCCACCATGGATATTTCTACCGTCAAGCGTTCCGGTATCAGGAGCATGGTTTTCCTGGCTTTTGCTGCCTTGCTTTGGACTGAAGTGGGAGCACAATTCTTTCTTCCAGGAGAGATCCCTATAGGAGCTATACTGGACCAAGTTTCTCTCAAACAGGAGGCAGCACTGCGTTACAAAGAGTCTCAGTTTAACAGAAACAAGGGACGTTACAAGGAATTTAACCTAACAGTTCAAATGCCTCCAAGTGACTCTATCAATCTGTCTTCCCCTAACCATCTGGTTGGTGCTATGTGCAAGCTGATGTCAAGAGGAATATTTGCAATAATTGGACTGGACCAGGCTTCGACCGCAGCTGCCGCTATAGATTTTGCCAATAAACTCCACATGCCTTATCTGACCCCCTCTAAAGGGGGAGTCACCCACCAGTTTGACAACGAATACGTCATCAACATGCTCCCTGATCATCTTGATGCTATAATAGAGACCATAATTTACCTGCGATGGAACTCTTACATCTATTACGTCTTCAATTCCGAGGGTGGGTTCTCTCGTTTTTCATACCTGAACCGTAGACTTGAAGAACTTAAAGCTCCGAAGCCAGTCGTGGGTTACCGTCTGCGTAACGCCTCTGATGCTTACATGGATCTGCGTGAGTTGGACAAACTGCCTCAACGTTTTGCTACCAAGTATATTATTCTGGATATGGCGGATCAGACTGCCCTGGAGGAAGTTCTTAGACAGATTCCAGTCGTCGGTATGAACAAAATCGGGTACCATTACCTCTTGGGGACATTGGCTATGGATGAACTGGACTTAACAAAGTACGAGTATGGAGGAGCTAATGTGACCGGATTTAATCTCCTCAATTACTCCGACTCCACCCTTAGACGTTTCCTGCGTGGATGGAGAAAAGAGAGCCCAGCTAAGTATTATGGCGCGGGCACAAACCGTATCCAGCTGCAATCAGCTTTACTAGTGGACTCCGTGTCTGTCCTGGCGAATGCGTTCAACTACATGATGGAGCGTAACCCGAGCATCTTTCGAGGCATCTTCCGTGACGGAGTCGTCTATAACAATGATGCTCCTGGTATTCTGTGTCGGTCACCTTCCCCCTTTCCATGGCATCTGGGAAATGAGATAATAAGTGCTATAAAGCAGTCCCATGTTTCCGGGCTGTCTGGAAAAATAGTCTTCGATAGAACTGGCAAGCGTAAGAACTTTACCCTTTCTGTCACAGATGTGTCGCTTAAAGGTCGATCCAAGGTCGGAGAGTGGTCGAGAGAGCGTGGATTCAAGGGCCCGAACTGGTTTGATACACCTAAGAAAAACGTCACCCACCGAGGGAATGGCTCCTATATCATTACAATGAACGAGGAAGAACCGTACGTGATGTGGAAGAAACAGGACAACGGAGAACCGCTGGTGGGCAATGATAGGTTCGAGGGTTACTGTGTGGACCTGACCAGGGAACTGGCCGCTATCGCAAACTTCAGATACACCATCAAATTGGTGGATGACAAAATGTACGGTGAATATAATGGCACACATTGGGACGGCATGATCGGCGAGCTGACTCGAGGTGAGGCGGACATGGCCATCTCCTCGCTGACAATCACCGCCCAGCGTCAGCGGGTTGTCGATTTCTCTAAACCTTTCATGCGTACAGGGGTCTCCATAATGATCAAGAAACCAGAAAAAGAGAAACCCGGGGTGTTTTCTTTCTTGGACCCTCTGGATGAGAAAATCTGGATGTCCATAGCTTTTGCCTTTGTCGGAGTCAGCGTCGTCATGTTCCTCGTGTGCCGTATATCTCCTCTGGAGTGGAACAATGAAGCTCAGAATGGAGAGGTCATCGAACTCGAGACCGTATTTTCACTGAAGAACAGCCTGTGGTTCTCCTTTGGGGCTGTCATGCAGCAGGGAGCTGATGTTTTCCCCAAGTCACTATCTGGTCGTCTGGTAGGAACCGTATGGTGGTTCTTCTCACTCATTATCATCTCATCTTACACCGCTAACCTGGCCGCATTCCTCACTATAGAACGTACAAATCCTAGAATCCGTTCTGCCGAAGATCTGGCTCAACAAACCGAAATTGAATACGGATGCATGGAGGGGGGATCAACAGCTAAATTTTTTAAGAACTCTCAATTCAAAACCTACCAAAATATGTGGGCTTTCATGACTTCCCGTCCTGGAGTTTTTGTTAAATCAACCGATGAGGGATTAAAGAAACTTCGTGAATCGAAAGGAAAATATGCTTTCCTGTTAGAATCTACAACAAACGATTACAAAAACCAAAAAAAACCTTGTGATACCATGATGGTTGGACCTAATATTCAAAACAAAGGCTATGGCGTGGCTACGGCCGTCGGACTTGAACCAGAAAACCTCAGGGAAAAAATTAATCTGGCTATTCTTCAATTAGATGAACGTGGAATCCTGACCAAGCTGAAGAAAAAATGGTGGTATGATAAGGGTGAATGTGCCCAAAAACCTGATTCCAAAACAAATCCGCTAAAACTGAATAATGTGGCTGGCGTATTTTACATTCTGATTGGAGGACTTATGCTGGCTATGCTCGTCTCGCTGTTTGAGTTCCTTTACAATGCTAGAAAAGAAGCTAAAAGATCTAAGGGTTCATTTAAAAACGCTGTTCGCTCTAAGGCCCGGCTATCTATAGCTGGCCCAGGAGAACATGAAGGGGACTATGCTGAAGAATGTGTCGCTCCTCCTCCTTCCTATGGAGCTGCGGTAATGCTGGATTTTTGAGGATCC

*Lingula anatina* GluA-g16613 (codon-optimized for *Xenopus laevis*)

GTCGACGCTGCCACCATGGAAACACCTGAAATCACGCTTTGGTTGATACTGTGTGCTTTTACGCGTCTGTCGATCAACGGTCAAAGAGCCGAGGCCTATGTCCCTGAAAATATTCCCATAGGAGCTATACTGGGCAAGGAATCGCGGCAGGTAGAAACCGCCGTCCGTTACAAGATAAAGCAATTCAATGGTAAAAAGGGATCAGACTACCTCGGTTTTAAAGTGGAAGCCTCTCTACACTCCGATGTCAATGTCGATAATCCTAATGAATTTATTGGATCAATGTGCGACCAAATGAATAAGGGTATCTTCGCCTTCATAGGAAGCAGCCATCTTCCTGCTGCAGCTTCGGCATTCGATTTCGCAGAAAAGTTCCACATGCCTTATTTTACTACCTCCCGAGGAGGAGTGACCTCCTTACGTAAACATTTCTATTCCCTACACATGCTGCCTGACCACCTGGACGCTATTATCGATGTTATGCTGCATTATAAGTGGACCGACTACATCTACTATATCTTCGATGACGAGGATGGATTCGGTAGGTTGTCCTACATATATAAAAGACTGGAAGAGCTGGGGCAACAGATACCAATTGCAGGAGCTAAGCTGAATGACACTAATAATGCGTACGCTGAGCTTAGAATGCTGGATAATCTTCCTCAACGTTTTGCCAATAAGAAGATCATCCTGGATATGTCATCTCACTCCGTTTTGAAGAAAGTCTTAGAACAGATTTCTATCGTTGGAATGAACAAGCATGGCTACCATTACATTCTGGGAACCCTCGCCATAGAGGAACTAAACCTGACTCAGTTTCGGCACGGCGGAGTGAACATCACTGGGTTCAGTTTACTGAACTATACTTATTACGAGCTGGATAAGTTTATCGAAGACTGGGCTAAGCCTCCCCCGGAAGGTACTGGAATGAAAACAATAGAGCTTACATCCGCCTTGTTCGTGGACTCCATCTCTGTGATAACCAAGTCCTTGAACACCATGATGATCAACAAGAAAGATGTCTTCTCCAACATTTTTAGAGATGGAGCGCTCTATAACAACGGGACACGTGGTATCAAGTGCTTTTCGAGGTATCAGCCAACCCCTTGGATTCACGGACCAGGAATAATTGAGGCAATAAAAACGCCGCGGGTTGCGGGACTCTCGGGTGAAATAGTATTTGACGCTTATGGAAAGAGGATAAATTATTCCATGGACGTCCTAGAAGTGTCCTTAGATTCCGGACCAATCAAGATTGGGGATTGGTCAAAGGAGAATGGCTTTCTGACCAATAAACTTGGAGAAGGGCTGGAGGACAAAAAACCAAATGTCACGGCTACCCGTACAATACAAATTATCACCACCGTACTTGCAGCGCCTTACGTCATGATGCGGAAGCCTGAACCTGGACAGATTCTGACCGGAGTAGATAGATACGAGGGCTACTGTGTGGAGCTGGCCAAGCTGCTGTCCGAGGTTGTCCCGTTTAATTACACCATTAAGATCGTGGAAGATGGTCAATATGGAACTCTGGATTCCAATGGTCGTTGGGACGGAATGGTGGGGGAGTTGACACGACAAACAGCCGATATGGCCATCGCTCCACTGACTATCAAATCCATGCGTGAACGAGTGGTAGACTTCTCCAAACCTTTTATGAGGACCGGAATTTCCGTCATGATTAAAAAACCAGAAAAGCAGAAACCAGGGGTATTTTCCTTCATGGACCCTTTAGACAGAGAGATTTGGATGTCCATCACTTTCGCTTTCGTTGGTGTTTCCATTGTTATGTTTCTTGTCTCCCGGATAAGCCCATACGAGTGGAATATCGTGAAGAACAATGGGGACGGTATGAAACTGAAAAACGCTTTCACTCTGAAAAACAGCATGTGGTTCTCCTTTGGAGCATTGATGCAGCAGGGAACCGACGATTGTCCAAGTTCAGTCTCTGGTCGGCTCGTTGGAAGCGCTTGGTGGTTTTTCACCCTCATCATCATTTCCTCATATACTGCTAATTTGGCGGCTTTTCTTACCATAGAGTCTATGGTGCCGCCGGTGAACTCTGCAGAAGAACTGGCAAATCAAGTCGATATTAAGTATGGCACCATGGAGAAGGGATCGACATATGACTTTTTTAAATCCTCGAAAATGGAAACTTATAAGAAGATGTTCAAGTTTATGTCGTCACAAGATCCAAAGGACGTCTTTGTCAGAGATAACGAAGAAGGCGTGAAAAAGGTCCAAAATTCAAAAGGAAAGTACGCGTATTTAGTTGAATCTACAACCAACGAATATCATAATCAGAGGAAGCCCTGCGATACCATGAAAGTTGGAGCTAACCTGGATTCCAAAGGATACGGAGTTGCTACGTACCAAGGGCATCCCCTCAGGGATGCTGTCAATCTTGCTATTTTGAAAATGCACGAAGATGGCACACTTACCAAGCTGAAACAGAAATGGTGGTACGACAAAGGAGAATGCGGATCTTCCTCGGATTCGAAAACAAACGAGCTTACCCTGAGTAATGTAGCAGGAATCTTCTACATCCTGATCGGCGGACTTGCCCTTGCCATGCTTGTCTCACTGTGTGAGTTCCTGTACAACTCCCGTCAAGATTCCATTATGTACAAGAAGTCCTTTAAGGACACTGCTAGAACTCAGGCTCGGCTGTCTATATCCGGGCGATGTGGAATGGGTGGGAAGGATCGTTCCAGCTTCGACAACTCGTCCGCATATGGAGTGCCGTACGCGTATACTGCTCCAAATGGAATGATCACCTTTAGAGGCTACGATGAGAAAACAGATACCGAAGTTTGAGGATCC

*Hofstenia miamia* GluA-98007595 (codon-optimized for *Xenopus laevis*)

GTCGACACCATGGTGGGCCTGCTGTCCCTGATTTTCCGCATTTTCCTGGCGTTTTGGCTGTTCACCAACGTGAATAGCCTTGGCTCTTTTAACTACACCGCGATTTTTAGCATTAGCGATAACGATTATGATCCGGATCAGACCGCGTTCGGCATGCGCCAGAACCTGACCTCCCAGGAAGAAACCAGCAGCAACACCGGTGTTAGCTTTACATTTCTGGCTTCTACTCCGACCGTCGATTTCGAAAATAGCTTCGCGCTGTCCAAACTGCTGTGCAAGCACCTGAAGGAAAAAGAACTGGCGGCTCTGCTGAGCACCGCGGCGTCTCAGGAAGCGAACAGCATGATTGCGTCCTACGCTAACGCGTTTAAAGTCCCTTACGTGAGCATCTCTCCTTACGAGATTGACGTGGATCATGATTACTACGTCAACCTGCGTCCAAACCTGATTAAACCAATTCTGGCTCTGCGCCGAAAACTGGGCTGGGAACGTCTGACCTACATCGTGGATTCTCAGGAAGGCATGAATCGCGTCCGAGATATTCTGGATAATCTGGAAGATAGCAGCATTGATTTTCTGGTGCGCAGCCTGAACACCAGCGTCAACGTGGAAAGCGTGCTGGCGGAGATTCGCGATGAATCAGAAACCAAAATTATGATTGATTGCAGCATGAACATTACCATTGAAATTCTGGAAGCTATGTATCGTATGCAGATGCTGCTGAAACAGTATCATATTGTGATCGTGAACGTGGCTGCGCCTAGCAACATTGATCCAAAATATGGCAAAGGCGGCGTGAACATTACCGGTTTCTCCATGTCTCGTGATGTGTTTACCAGCCTGGTCGAATTTCAGCTTCATTGGAAAACCACCATCGAAACCCAGCCGAAAATTACACCATTTTATGAGTTTAGCTCTATGCTGGCGTATGATGGTTATGATCTGCTGTATAAGGCTTTTAAACAGTCTCAGAAAGTTGCGGATAACCTATACCGCCTGTCCCTGGAGCGCAACCTGTCCTGCGAAACCGCGGGAAAGGTGAACAACGAAATTCGCCAGAAGGCGGATATGATTGTGAAAGTGATTAAAAACATGCGCATGAATGGCATTACCGGCAACATTCAGTTTGATAAAAACGGCCGCCGTACCAACTACAGCCTGAATCTTGAAATTCTGCCGGAAAAACGCGGCACCGATCCGGTGTATGTGGGCACCTGGGATCATGATCTGGAAGATTTTGTGCTGGACGGGAATTTCCGCTTTAACCAGCGAAACCCGAATGAAATGAAGCGCCACCTGATCATTACCTCCCTGATTGAACCTCCGTTCCTGATGAATGTGACCGCTCAGGATGGCACCATCCCGACAGGCAACGAACGTTTCGAAGGCTACTGTAAAGATCTGGCTGATAAACTGTCCAAGGACCTGGGTTTTACCTATGAAATTGTACTGGTGGAAGACGGCGAGTATGGCGGAAAAGTGAACGGATCATGGAACGGACTGATCGGAGAGGTGATTAGCGGCAAAGCGGATCTGGCTCTGGCTGGCCTGACAATCAACGCTGAACGCCAGCGTGTGGTCAGCTTTACCAAACCTTTTATGAACATTGGCATTTCCATCATGATTCTGCGTCCCAAAAAGAAACTGCCGGGCCCTCTGAGCTTTCTGGACCCGTTTTCCAATCATATTTGGATTGGCATTATTATTGCGTACGTGTCTGTTAGCATTGGCCTGTTTATTGTTGCCCGTTTTAGCCCAACCGAATGGCGCATTAAAGATACGGGCCAGCCTCCGTCCACCAGCGGTACCCATCAGATGTGTCACAACGATTTTTCCCTGTACAATTCAATGTGGTTCTCCCTGGGCGCGCTGATGCAGCAGGGAACCGATATTGCACCGCGCGCTACCGGTTCCCGCATCGTGGGCGGAGTGTGGTGGTTTTTTACCCTGATTATTATTTCCAGCTATACCGCGAACCTGGCTGCTTTCCTGACCGTGGAAACCATGGATACCCCGATCAAGAACGCAGACGATCTGCAGTCTCAGACCGATATCGCTTACGGCACCCGCCGCACCGGTTCAACCCGTACCTTTTTTGAAAATTCTGAAATTGACGTGTATAAAAAAATGTGGATGTATATGAAAGATAAAGAAGATGTCTTCGTATCAACCAATGATGAAGGCATTAAAAAAGTCCGTAACAGCAATGGCAAATATGCGTATCTGATCGAGAGCGCTGCGAACGAATATGTCCAGAGCCAGAAACCTTGCAATACCCTGATGGTGGGCCCGAACCTGGATTCCAAAGGCTACGGCATTGCGGCGAACCTGAACTCCACCTGGATTAAAAATATTACCCTGAAGATTCTTAAGTATCAGGAAGATGGCGAGCTGGAACGACTGAAGACCACATGGTGGGTGGATAAAGGCGAATGCGGCGGCGCGTTTAGCAGCACCAAAACCGATAGCACGAATAGCCTGGGCCTGAGCTCTGTCGCGGGCATCTTTTACATTCTGATCAGCGGCCTGGCGCTGGCAATTATTTCTGCGATTCTGGAATATCTGCTGAAAAGCTGGCAGCATTCCATTAAAACCAAATCCGGTGTGCGTAACAGCCTGCGCTATAATGCGGAACTTTCCGTGATTGGCCGCGAACAGCAGGCGCCGCCTAGCTATACCTCCTATGTCAATAGCAGCCAAAGCACCGATAGCATGTTCAGCCATCCGATTCAGGGCTTCAGCTCCCCGGCGGCAAGCCTGCATAAACGTACCAACATTTATGAGCATAATTCCCCGAACACCATCCATTCCACAGTGTACGATTCCCTCCCTAGCCCGTACGCAACCAAACTGGTGCACAACAACAGCAAATCCAGCCATACCGATGTGTGAGGATCC

*Hofstenia miamia* GluA-98014323 (codon-optimized for *Xenopus laevis*)

GTCGACACCATGATGCTGGCGCTGATTTATCCGCTGTTTATGTTTCTGCAGTCTCCCACAGTTTCACTGGAAAACTTTAAGATTTTTGGACTTCTGGATCGTCATCTTCTGGAAGAAGAAATTGTGTTCAACTATCTGTCAACCCCGACCTATAATGAACCGAAGCACTATATTGCTGTGGAATTTCAGACCAACAAAGTGTTTGATCTGACGAAAATGCTGTGTGATCGCTTTCATAACCAGGGCGTGGCTATTTTTACGCCTGAACTGCCGATTGTGGAAACCCGCGTGATTAACAGCTTCGCGCGCGCGTTTCGAGTGGGCCATGTAACCCTGAGCCGCGCGCCGCTGCCGAACACCTTTCGTATTCGCCCGAGCCTGGCACGCCTGGTCGCGGAGCTGACCATGAAATATTACATGTGGGATACCTTCGTCATCTTCTTTCAGTATGAGGAAGATGTCATTGAACTGGAAGAAGAAATTAACATTCGCATTCCTTCTGGCATTTACGCGAACTATCGCCGTTTTAACGTGAATAGCAATAGCGAAACCGTCGATAACCTCCTGATTCAGTATCAGAAAATCGGTAAAGGTCGTTTTATTGTGGCGTGCGACCTTGAAACCACAGTCTCTATTATGAGCGCTGCGCAGAATCTGCAGATGCTGAGCCGCGATTTTCATTACATTCTGGCTGATATCGAATCTCCTTGCTCCCAGCTGAATCTGACCAGCTCCGGCCGCATGAACATTACCTGTTTTCAGCTTCTGTCCAAACGCAAAATTAACGATCTTTATACCGATGTGTCCAAATCTCGCCTGGATATTTCTACCAAAGCTTTTCTTAACAGCAACGAGTTCAGCAAACTGAAAATGGCACTGGCTCATGATGCGTATAAGGTGATTAAGTCTGCGTATGATCGATTTCTGCATGCTGCTCCTAACAACTATAAAGCGCCTTCCGATCCACCATGCCTGGGCAAAGAAATTAGCAAAATTCCGCGTTATCTGCTGTATAAAGAAATTGAAAATATTCGTCTGGAAGGTCTGAGCGGCAATATTCAGTTTAACAAATTCGGACAGCGTGTGGATTTTAACGTCACCATTAGCGAACGTCCGTTTAGCAACGATAAGGACGTCAAAATTGGCACATGGACCGATGATAAAGGCCTGCTGCTGACCAAAGCGGGCCGCAGCCTGCCCAGCATGAGCTCCGCGACCGTGAGAGTGGTGACCATTGAAAGCAAACCGTTTACCTCTATTAAAAAAGGCAGACGCCCTGAAGAAGGCAATGATGCGTATGAAGGCTTTTGCATTGATCTGTTTTCCGAAATTGCTAAAAACCTGAATCTGAGCTACGAACTGCATCTGGTTGCTGATGGTAAATTTGGAGAAGAACTGTCAAATGGAACCTGGAACGGCATGATCGGCGAGCTGATGAGCGGCAAGGCTGATGTCGCGCTGGCGAGCCTGACCATTACCTCTGAACGCCAGAAAGTGGTGGACTTTACCAAGCCGTATATGGATGTCTCTGTGAGCCTGATGGCGAAAATTCCAGAACATGAAAAATCCAGCTTCGCGTTCCTTATCCCTCTGCATTACGAGATTTGGCTGTGCATTCTGTTTTGCTATATTAGCGTTTCCATCGTCTATTTTATTACCGCGCGCATTTCCATGATGGCTTGGCGAGATGGCGATGAACGCTATTATGATCAGAACGGCTTCAAATACACCATTTCCAACTGCTTTTTTCTTACCCTGGGCGCGGTGCTGCAGCGTGGCGAGGATATGTGTACCAAAGAAAGCCCTTTCAGCTCTCGTCTGGTGGGCGGAGGCTGGTGGTTTTTTACCCTGATTATCATCTCCAGCTACACCGCTAATCTGGCTGCATTTCAGACCGTGGATCGTATGAAGACCGAAATTAATTCAGCGGAAGATCTGGCCGCTCAGACCGAAGTGCTGTACGGTTCCATTGATGGCGGCAGCACCAAAAGCTTTTTTAAAAACTCCCAGAGCGGCACCTTCCAGCGCCTGTACATGTTTATGAAAGATCGCAAGACCTTTACCAAAAGCACACTGGATGGCGTGCAGAAGGTGCGCGATAGCGATGGCAAATTTATTTTCCTGCTGGAAAGCACCATGAATGAATATTATAATGCTCGCCCTCCGTGCAACACCATGCGCATTGGCGGCAATCTGAACCTGAAGAACTACGGCATTGCGACCCCGCAGGGAAGCCAGCTGCTGGAGAACTTTACCGTGCAGATTCTGGAACTGGGCGAAAGCGGCTTTATCGCTAACACCAAAGAATCTTGGTGGTACGAAGGCAGCGAATGCGTCGAAAAGAACAGCAAAAAGGAGGTCGGCAAGGATACAAATGCACTGGAGCTGGATTCTGTGTTCGGCGTTTTTTATATTCTGTTCGCGGGCATGGCACTCGGATTTAGCGTGTGCATTTTTGAGTTTCTCGTGTTCTGCTACCGCCAGTCCCGCCAGTTCAAGGTCTCTTTCTTTACCGTCGTGCATGCGAACTTCGTCACATGCTTCAAATCAATGTTTGAAATGAACAGCATTGGCAGCCTGTCCCGTGTGTACAGCCCGATAAAACAGACCCAGCTGCCGATTGCTAATAACAAAATTCATCGCCTGCTGTCCTCCCAGCAATCCAGCTTTGAATCCAACATCAGCGATTGCCCGGTGCTTCCTTCTAAACCGACCACCAACAACTTCCTGCCGGTGCAGCATCGTCCAAACAGCTTCCAGCTGGCGGCTGTTCGCGTGAGCACCAAGTCCAGCCCTAGCAATCATACCACCGTGTAAGGATCC

*Hofstenia miamia* GluA-98019615 (codon-optimized for *Xenopus laevis*)

GTCGACACCATGACCCTCACCTCCCATTGGCGTTATCTGTTCTTCTTCACCCTGTTTACCTTTCCTCAGCAGGAACTGGCGTTTAACAAGTATAAGATTGCGGCGTTTTTTCCTGAAGATGGCAGCCTGGAACGCAAAATTTTTCAATTTGCTGCGGAAGAATACAAAGATATTGAAATCGTGTCCCATGAATTTAGCACCTCCAATCTGTTCAATCTGAGCAGCACCCTCTGTAACCTGGGAAAGAGCGGCATTGTGTCCGGCTTCTCCGGAAGCAATCAGTTTGCGACCACCTCCGTGATTCAGTCTTTTTTTGATGCATTTGATCTGGCACACGTGACCTATCAGACGGAACATTCAAAAATTAATCAGGCTTTCGAACTGACACCTCGTTTTGGCCCGCTGATCTATTCCCTGCTGACCAAGATGGAAGTCAAAAAGGTGTACGTGATTTTCGACAAACCTCACGCAATGAACCGAATCCCTGAACTGGTGAACATGGGCCCATATGTTATTAAAAATGAAGTCCTGCGCATTAACGATAATACCACCCTTAAACGCCGTATGCAGGAAATTCAAAACGGTAGCATCCGTAACATCATTCTGATGCTGAGCTATCCGCTGACCGAACTGTTTTTTCAGAACGCTCTGAAAATGCAGATGCTGAGCCCGGATTACCGCTATATTATTGCTGATTTTAGAACCCCGTGCCGTAAACTCAAATTCAAAATTACCGGCAAGATTTCCGTGACGTGCCTGCAGCTGGTCAATGAACAGGCTCCACTGGTGCAGGCGTTTATGAAACGTTGGCTGCAGTCCGTGCAGTACAAGGAATACACCAAACTGCATCCGAAAACCCAGTTTTACCGTCTGCCACCTAATGAAATTAAACTGGAAATGGCTCTGGCATATGATTCATTTCAGGTGACCAAAGCTGCGATTAAAAACGTCTCCCCGTATAGCAAAATCGGCTGCGAAAACCTGGATCGCATTTGCATGTCACGCTCCATCAAGATTAACTGCTTCGGAATTAAACTGAAGCAGCAGCTGAGCCGTATTAAAATCAACGGCCTGACCGGCAATATTGAATTTGATGGAAACGGACTGCGCAAAAATTTCAAAATTGAAGTGTGGGATGTTGGATTTATGGAATATCACTCCAAAGTGGGCGTTTGGACCGATGGGGAAGGCTTCACCGAAATTACCAGCTCCAAATTTCCGGCGAGCTTTAGCGATATCAGCGATCGTGTCATGCGCGTCACAACCGTGCAGAACTCCCCTTTCACAATGATTAAAGAGGAATACAAAAACTGGAACAGCAGCGAGAAAGGCGATCTGCCGAACGATGCGTTCGAAGGCTACTGCGTCGATCTGATTAAAGAGGTCGCGAAAGAACTCAACGTGAGCTATAAACTTACCCTGGTCAAAGATGGTAAATTTGGTGCGGAGACAATTACCGGTAAATGGAATGGAATGATTGGCGAACTGCTGGAAGGCGAAGCTGATGTGGCGCTGGCTCCACTGACCATCAACCTGAGCCGCCAGCAGGTTGTGGATTTTACCAAACCTTTTATGACCCTGGGCATTTCCATCATGATGCGCAAACCGATTAACAAATCTGATGTCTTTAGCTTTCTGTACCCGCTGCACCGCGACATTTGGATGTGCTGCCTTTTTGCTTATATGTCCGTGAGCGTGGTCCTGTTCCTGGTCTCTCGTTTTAGCAGCACCGAATGGCATAGCATTAGCGTCGGAAACGAAAATGAACTCTATTTTGAAAAAGAGAATGAATTTGGCATTATTAACTGCCTGTGGTTCAGCCTGGGCAGCTTTATGCAGCAGGGAGGCGATGTGTGCCCGACCTCCATGCCGGGCCGCATTGTTGCGGCTAGCTGGTGGTTTTTTACCATGATCATCATTAGCTCCTACACCGCGAATCTGGCTGCGTTTCTGACCGTTGATCGCATGAAAACCCCGATCACCGGCGCGGATGATCTGTCCTCCCAGACCGCAATTGAATACGGAGTGCTGGATCAGTCCTCTAGCCAGGCTTTCTTCGAAAAGAGCACCATGCGCACCTACCGCCGCATGTGGTCCTTCATGAAAGAAAAAGGTAATTTTGAAAAGACCACCGCTGATGGCGTCAAACGTGTCATTGAATCTGATAACTACGCGTTTCTGATTGAATCATCCATGCTGGAATATTACAACCAGCGCAAACCATGCACCACCGTTCAGGCGGGATATAACCTGAACGTCATTAACTATGGACTAGCGACCCCGAAAAACTCTCCTTATCTTGAAAATCTGACCATTGTGGTGCTGCAGCTGAAATCCAACTTTCTGGCGGTCAAGAAGCATGAATGGTGGGTGGAAAAAGGCGATTGCAACGAAGCGCCGATCAAAAAAGAAGATACCGGCGCGAATGCGCTGAAAATTGATTCCGTCGAAGGCATCTTTTATCTTATTTCCGGCGCGATTTTCTCCGCGGTGTTTATTACCGCGTTTGAATGGGTGTTTGTGTCCGTGCAGGACTCTCTTCGTTTTAAAGTTCCGGTCAAAAAAGCTCTGAAATCCAATTTTAACATGGCTGTGACCAGCGGCTACGATCCTCCGAACCTGTCCCCTCGTCAGAACGAAGAAAAGAAATACTTTGGCAGCCAGCGAAAAAGCTATCATAACGAAGCTAAGAATCCGACCGATGTCTCATACTTTTCTCCGTCCCATACCGATGTTTGAGGATCC
