# Supplementary material for: Tunable AMPA receptor function via recurrent evolution of heterotetramers
Source: J Gen Physiol. 2026 Aug 27;158(6):e202614039. doi: 10.1085/jgp.202614039 (PMC13520880; doi:10.1085/jgp.202614039)
Supplement: Supplementary file 2 [file jgp_202614039_tables2.docx]

**Table S2. Synthetic TARP genes used for expression in *Xenopus laevis* oocytes**

*Saccoglossus kowalevskii* TARP-XP_006816113.1

GTCGACACCATGCCTAGTTGTAGTGAACGTATGTTAGTAATCTCGACCACTGCTGGCTATGTAGCATGTTTGTGTTTATTGAGCACTGGTGTGGCAACGAGTCATTGGCTTTATTCCGTGGAACGCTCTGAATATAGTACCTTCAATGGTACGACGATGGTCGTGGAAGTCACTATGCATTCTGGTCTATGGAGGCTGTGTCCGTTGTCAGGTGAGAATGTTGGTGACTGTAGAATCATTGAATATTTTACACCAAATCCTGAGATTACATCCGATACAACAACAGCCGTTGCACGTTTAATTCGTTCATCTGCTGCATTCCCCTGCGTTAGTCTGCTGTTAATGATAGTGGGTACTGCATTGGGCGTTGCATCCCAAAAGAGACCGGAACAGAAACACCTGGTTTTCGTGGCCGGTATTGTGTTCGTCACTTCCGGTCTCTCTTTGTTCGTTGGAATTATCATATACATCAGTAGTATAAACGACGCCCTCAGCGACCAACCAACTTCTAACGGTCACCGGTACACCTATTATTACGGGTATTCGTTTGTACTCGTCACGGCGTCATTCGGCCTTAGTGAAGTATGTGGCGTTATGTGTATGTATCTATACATAAGACGTTATATGAACCAAGAAGAGAACGAATCAAGACGAAAATCTCGTCTACGCAGATATAATGTTGGGAATATTGAGGAAAATGAAGTCGCAAATAGATTGGTTTATCGATGTCCAGAAGTGATGATACCAGCGTGAGGATCC

*Saccoglossus kowalevskii* TARP-XP_006822518.1

GTCGACACCATGGCTCGTTCGTGTAACAGAAGGCGGCTGTTGATGCTCTTGACGTGTTTGTCTAAATCATCTTCGGCTATCATGCTGGCTATAGCGTTGTCCACCGATTTTTGGATTAATACCAAGGACGTGAGCTACAAAGAAAAACAGCTGGCAGGGGAGGAGGGATCTGAAAACGAGACAGCCATCCACGAACACATCGGACTGTGGCGAATGTGCACCAACTTAGGAATGGGATACTTCGTCTGTACTAGAATCGATTACTTCCCAGAGGGGCTGGAATCTGACAGCTCCACAGACTCGGAGGGAAAAATACTACGTGTTGTTCGAGCTTCAAGTGCCTTCCCTATACTTGCTCTCATCGTACAAACTATCGCTACTATAGTGTGCTTTGTCGGCCATTATAGGAAGCACAAAGTACATTTGATACTCATTGCAGGAGTTCTATCCGTCCTAGCAGGTTTGTGTAGCATCGTGGGATTATTTATTTATATATCCAGTGTCAGTGAGGAATATGCGAGCAAAGCACGGCCACGGAGTGCCATCAGTGATCCTACTTTCTACTACCATTACGGGTGGTCATTTTGGCTTGCTGTTTCTTCGTTTACACTAACAGAAGCAGCTGGTGTATTATCTGTCTACCTTTACGTCTCACGCCATAGGCACGTCATTAAAAAACACGTTGACTCGAGAAAGCACGTAGATGCATTTCCCAGATTACGGTCAGATTCAGGGTCGCAGGATATGTACCGTCTCACCCGCAACCAACACGACTATTATAGCGCCCCACATCTGCAGGCATCTGACTACAGTACTGATAGTTCCAGGGATATCGGCATGAGTTTACTCAATTCAACTGAACGACACCCTTCAAGGAAACTGTATATGCCCAGAAGAGAACGTCGGTTGCCACGACAACATTCCTTTGAAACGGTCCGTAAAACGACACCGGTGTGAGGATCC

*Lingula anatina* TARP-g12410 (codon-optimized for *Xenopus laevis*)

GTCGACGCCACCATGAAGTGCAACGAGCGAGCTATGACGCTTCTGGCTCTGGTCTTTGGGACCGTAGCCACCACTGGTCTTTTCCTCGCTCTTACCACCACATACTGGCTGTACACGACAGAGAGACCGGAACAGCTTTCCTCGTCGGCGGCTAAGGAGAAGAAGAATACCACAAGGGAAGAAGGGAATGTCACGTATGCCCATCTTGGTCTGTGGCGTGTGTGTTTATATTCCAAGCAGGACCCTGATCGTCCCTCCGAATGTGCCTCCATCGATTACCTTTCCAAGGACCCACCACAGCTGCTGGGACCCGAGAGAAAAAAACTGCCGTCCGAAATAACCCTCGCTCTTGCCAAGGGCACAGGCATCGCCGCACCTCTATGCACTAGTGGACTTGTCCTGCTTTTGATTGGGATAGTGATTTCTGCTATTGGCATAGTGAAACGTGATTTCAAAATTCTGATCGGATCAGTCTTTCACATACTGGCTGGTTTGTGTCTCGCTAGTGCCGTTATAATCTATATCTCCGCTATCAACGACGAAGTAGCCCACAGACCTCAGCCTCAACCTGATGAAAACCCCGAGAACTTCCAATTCATCTACACGTACGGATGGTCGTTTTACTTCGCTGGGTTTTCTTTCATATTTACAGAGCTGGATGCTATAATCTCCATTTTATTATACCTGAAACGTTACAAACACCTGGATGATAAGATCCAGATGGTGCCTGGGTTGTATAGAAAGATAGAAAAGCATAATAAGGGGGGTCACACCAACCAATCTTGTGATACGTCTGTTGAAGCTAATACATCTACCCGTGGATCTCCGACTCTTATCCTGTAGGGATCC

*Lingula anatina* TARP-g21034 (codon-optimized for *Xenopus laevis*)

GTCGACGCCACCATGGCGTGGTGTGGTCGTAAACGTTTTCTGATGGTCTTAACCAGTCTCTGTGCCTCTACCGCGTTCGCTTTCCTCTCTATGGGTGTTGCCACCGACTACTGGATATACATGACAGAGATAGTGACGGGGTGCAATCAGACCGAGGTGACTAAGTCCACACATGCAGGATTGTGGAAAAAATGCTATTCACAAGAATGCATGAAGGTTAAGCTGAACTGCAGTTACATCAAGTACTTCAGTCTTGAAGAAAAGGATGATTCTCAGCGAACCGAGGCTATACTTTATAACATCAAGACTTCGACCATGATGCCATTAATCAGTCTGCTTCTGCAAATGACGGGAGGTATAGTCTGCTTCGCCTCCCATTGCTATGACAAGAAGTTATACCTGTCCTTTGTTGCTGGTGTCCTGTTTGTCCTGGCAGGTCTCTCTACACTGATCGGACTGATATTCTACATTGGGGCAATTGCATCCGAAGCTGCTCATGTCCGTCCACCCGGAATAGGAGAAAAGCCTGTCTTTACCTACAGATACGGTTCATCGTTCACTATGGTCGCTACTTCGTTTGCCATTATGGAACTTTCTGGCGTGCTATCCGTTTACTTATTCATATTCCGTTATAAATCCGAAAACAGAAAGAAGCGTGAAAGGTTACGTGCTATCAATGATTCCGTCCAGCCTACTGAGAATCATGTTGGAGGCGGTGCCAGCCACGTGATTAGAAATTCACGTCGTCGTCGATCACGCTCAAGGTCCCATGACCGCGAGCACTCTCGAGAAAATTCTCCTTCGCATTCTGAACGTACCTACTTCACCTATGCAGCCGGAATGTCTCGTGAAACTTCAAATTACACACTTTCTCGTGACTTTCCAGGACAAAACCATGCTGATATGTTCCTTCTTAAGCCTCTGAATCCTAATAACCATCATTCGGATTCTTCCCACCGTATGACCCCCGTATGAGGATCC

*Hofstenia miamia* TARP-98036255 (codon-optimized for *Xenopus laevis*)

GTCGACACCATGGGTGAAGATGAAAAAGTGCGCCAACTGAAAATTCGTCTGTTCCTGATTATGACCAGCTTTTGCAGCATTCTGGCCCTGCTGACCATTCTGCTGTCTTTTTTTTCTTCTTATTGGCTGCTGACCGCGGAAACCTGGAAAGGCCCTTCAGAACGTAACGAATCAACCGTGAAAAAAGATGTTCATTCCGGCCTGTGGGAATTTTGTTATTATGATAAGAAGGATCTGATGAACAACGAATGGAAAGATACCATGAACTCAGATGGCTGCGTTCCCATTTCCGATAGCAAGAGCGAAAGCGATGATGAACGTCCTACCAGCACCCGCGGCGCGCTCAAAAAAATCACACATTATTCCGGCATCCTGATGCGAATCTGTCTGGCTGTGCTGTCCACCGCGTTCATTATTAATGTCGCTGGCCACGTTCTGAAAACCAAAATTCATATTTGCTTTTTTGTTTCAGGAGTGCTGTTTGTGTTTAGCGGCCTGCTGCTGATTCTGACCCTGGTGATTTATGCTTCCAAAACCAATGAAATTGCAGAAAGCTGGCCGACCGTCGTCGAATCCGAAGATAAGACCCCTGGCAAGATGAAGTATCGCTATGGCTGGTCCTTTTTCTGCTGCGGACTTTGCCTTATTGCAAGCGAATGGGCGGGTGTGTTTGCGATGTATTTTGTGCTGGCGCGAGCGAACGATATTTTTATTAGCCAGCTGAATAAAGATTCCAAAGAAGAAAAAAACAACGATGGCCTGCTTTATAATTGCGATTACAGCTTTAGCGATGCATTTTCTCGCCAGATGATTACCAACATGCGTAGCATGACAAACGGCAATAACGCTCGCCTGAACGCTTCCTCCCAGATGACCACCCTGTGAGGATCC
