# Supplementary material for: Tunable AMPA receptor function via recurrent evolution of heterotetramers
Source: J Gen Physiol. 2026 Aug 27;158(6):e202614039. doi: 10.1085/jgp.202614039 (PMC13520880; doi:10.1085/jgp.202614039)
Supplement: Supplementary file 3 [file jgp_202614039_tables3.docx]

**Table S3. Synthetic AMPAR genes used for expression in HEK293 cells**

*Saccoglossus kowalevskii* GluA (codon-optimized for human)

GTCGACATGGGCCCTTGCGTGCAGCTGTTCATCATCCTGGCTAACTGCCTGGTGCTGTTCGTTGCATCTATCCAAAGCGCCGCTTCTATCCAAATCGGCGGCATGTACAACGCCCATCCTTCGCAGGAGCAGAGTGCCTTTAGACACGCCCTGTTCCAGAACAACATCCACAGCAACACAAGCATTCAACCTCTGAACCTGATGGCCCACAACGAGAACCTGGATCACTCTGATAGCTACGTGCTGGCCGATAAGTTCTGTACCCTGATCAGAAGACAGGTGTACCTGCTGGTGGGCGTTACCAGCATGAGCAGCCTGCCTAGCATCAAATCTTACAGCAATACCTTCCACATCCCATACATCACCCCTAGCCTGAGCGGTACAGAAATGGGCACAAAATCTAGCGATTACCTGATCTACATGAGACCTTCCCCCATTCCTGTGCTGGCCGGCCTGATCAAGTACCACAAATGGGACAAGTTCAACTACCTGTACGACGACACCGAAGGCCTATACCGCCTCCAGCTGCTGCTGGAGGTGGCCGCCGACAGATACCTGGACGTGCGGCCCTACCACCTGCGCGGCGTGACAAGCGTGATTAACATCCTGAACAAGATCAGAGTGTCCGGCATCACGAAGACCATACTTGACACCAGCCCTCCAAGCAGAACATCCGCCATCTTAACCCAGGCCCTGCAGCTGGGCATGGTGACAAGAAAGTATCACTTCGTGGTCCTGGACTTCGACGTCTACCACATCGACCTTGAGAACTTCAAGTACGGCGGGGCCAACGTGACCGGCTTCCGAATGGTGGATGAAACCAACACCAGGCTGAAAGAATACAGAGATGTGTGGAATCAGTTCACCGAGAAGTATGCCGAGTTCGAGAATATCACCTACGTGAAATTCCAAGCCGCCCTGGTTTATGATGCCGTGTCCGTGATCACCGAGGCCTTTCGGCTTATGAAGAACGACCACGTGGACATCGACAGCGAGCTGATTAACTGCGACAACAAGCACCAGAAACCTTGGCGGGACGGCGAGAAAATGAACAACTACCTCAAAGAAGTGGACATCCAGGGCCTGACAGGACCTATACGGTTCGACAAGCTGGGCAGAAGAACCAACTACACCATGGACATCCTGAGCCTGGTCCCCGACGAGACAGGCAAGGCCAGATTTGACAAGGTGGGCAAGTGGGACAGCAGCGGAGAGGGCCACATCGGAATCGGCAGCGACGGCGAGGTGCTTTCTTTCCCCGCTGATTCCTTCAACCGCACCCTGCGGGTGACCACCATCGACGACGACCCTTACTTCATGCTGAAGAAAAACGCGGACGAGTTCGAGGGAATGGACAAGTATGAGGGCTACTGCGTGGACCTGATGGCCAAGATCGCCGACGTGCTGAAGATCAAATATGAGTGGCAGATCGTGGAGGATGGAAACTACGGCAGACTGAATGAGGATACAAACAGATGGGATGGCATGATCGGCGAGCTGATTGAGAACAAAGCTGACCTGGTGGTGGCCCCTCTGACGATCACCTCTGATAGAGAGCCTTATGTCGAGTTCACCACACCCTTCATGGGACTGGGCATCTCCATCATGATCAAGAAGCCCCAGAACACCAAGCCCGGCGTGTTCAGCTTCCTGCACCCGCTGTCCCACGAGATCTGGATGTGCATCGTGTTCGCCTACGTGGGCGTGAGCGTGGTGCTGTTTCTGGTGTCTAGATTCTCTCCTTACGAATGGCATGCCGTGGACGCCCCTCCTCCTCTGTTCGACGATACAGGCGACCGGCAGGAGATGGCCAACGACTTCGGCATCTTCAATTCTCTGTGGTTCAGCCTGGGAGCCTTCATGCAGCAGGGCTGTGACATCAGCCCTAGAAGCCTGAGCGGCCGGATCGTGGGCGGCGTGTGGTGGTTCTTCACCCTGATCATCATCTCTAGCTACACCGCCAACCTGGCTGCTTTTCTGACCGTGGAAAGGATGGTGTCTCCAATCGAGAGCGCCGACGACCTGGCCACACAGACAGAGATCAGCTACGGCACCCTGACCAGCGGCTCTACCACCTCCTTTTTTGAGAAGAGCACTGTGCCAACATACACCAGAATGTGGGCCTACATGTCCACAACCCAGCCCAGCGTGTTTACAAACAGCATCAAGGAAGGCATCGAGAGAGTCAGACGGCAGAAGGGAAGATACGCCTTTCTCCTGGAAAGCACAATGAACGAGTACATCAACCAGAGAAAGCCCTGTGACACCATCAAGGTGGGCAACAATCTCAACAGCAGAGGATACGGAATCGCCACCAGAAAGGGCAGCGCTCTCCGGGAGAACCTGACCCTGGCTATCTTGACACTGAGAGAACGGGGCGAACTGCACAAGATGGAAAAGAAGTGGTGGTACGAAAAGGGCGAGTGCGGCGACGGCGACTCCAAGTCCCAGGAAACAGCCAATGCCCTGAGTCTGTCTAATGTGGCCGGAGTATTTTACATCCTGGTGGGAGGCCTGGGCCTCGCCATGGTCGTTGCACTGATGGAATTTTGCTACAAGAGCCGGAAGGAGGCCCGACGGGCCAAGACCTCTCTGAGCATCGCTATGCGGGCTAAGGCTAGAATGAGCATCAAGGGAGATAACGGCAGAGTGAGCACCCCTGATTTCACAAAAAGCATCAGCACCGTTCCAATCACCAGAAGCAGCAACGCTAATAGCGGCAATATGTTCTCACGGAAGAAAAGCCTGACCCCCTGCCCTTCGGAGGAAGATCTGCTGGCCAAGGTCAAGAAGGAGGAGGTGTACCTGAGAGAAAACGTGAAGGTGAAGTGAGAATTC

*Lingula anatina* GluA-g8707 (codon-optimized for human)

GTCGACATGAATCCTGCTGCCTGGATCTTCGTGGTGTCCGTGTACTCAGGAGGCGTGGTGTTCGGCAAAATCCTCGAGCACAGCGTGGGGCTGATCTTATCCAACACCGCCCCTAGACACGGCGATATCGAGCTGGCTTACAGATACGCCTACCTGGGCACGCTGTGTAGACATAATGCCCAAGGATTCATCGAGGGGAGAAACTACACCAATTACAGATTCAAGTTCTCTTACACACACAGCCTGAATTTCATCAATATCGGCAACAATTTTGATTACAACTACACCATTTGCAACACACTTCAATGGAAGGTGTTTGCCATCTTCGGCATCTACGACACCTTCACGGCCGACTTCGTGGCCAGCACAACAGGCCTGTACCACATCCCCTACATTACCCCTACCAGCCCAGAGATCAGCTACAGACCTCACGGCGATTACATGATCCATATGCAGCCTAGATACGCCCAGGCCCTCGTGGATTATATCCTGCTGAGGAGATGGAAGAGCGTGACCTACATCTATGACAGCAACAAGGAAGGACTGGCTAGAATGTACCACGTGGTAGGCGCCATAAACAACGCTGGCGCCTATGTGGTTATCCACTTCAAGTACGTGACCAATGTGACCGACGCCCTGGCCAAGCTGGCCGCTATGGACAAGACAGGCAGCGAGAAAAAGCACAACGTGATCATCGATATGAGCCTGAACGACACCAACACCGTGCTGAAGCGGAGGTTCACCGAGACACCTCAGACAAAGCTGCTGTACAACTACCTGATCGCCAGACTGTTCACCAACGAGATCGATTTCAAGGCCTTCCCTGCCGGAGGCATGAACATCACAGGCTTCCACATGGTGGACTACGACACAGACGTGACAGTGAAGAAGTACAACCAGGTGTACCGGCGGTGCGACCTGAAGTGCGGCTCCTTCGACAATACTTGCCCCACCAGCAACGGCGCCGCCTTCGCCGCCGACAGCCTGACCGTGTTTATCAAGGCCATGGACCTGCTGCACAAGAATGGAAGCTCCAACTTCCCCACTAACAACCCTTATCCAAAGAACAGCGGCGGCCTGGCATGCGCCGAGTGGCCTAACGCCCCCAAGCCTTGGATCTACGGCGAAAAACTGATCGGAGCTCTGAAGAAAGTGGAACTAGATGGATTGACAGGCAAGATTCTCTTTGACGAGGATGGCTACCGGATCAACTTCACCCTGTATCTGTTTGGCACCACCAGCCAGGAGGGACCTGCTAAGCTGGGCACCTGGGAGAGCCACCCTAGGCGGCTGAATAGACCAAGACTGAATCTGATCCACGACACAAAAAGCGTGGGCCTGCTGAACGTGGACCCCGACAACAAGACCTACATCGTGACCACCATCCTACGGGACCCCTACATGATGGAAAAGATCCATCCTAGACCTGACGGCGGCAATTGGACAGGCAACGACCGGTACGAGGGCTTCTGCAAGGATATCATCGACCAGCTGATGAGAGATCTGAACATGAACTACGAACTGAGAGTGGTCGCCGACGGTTCTCACGGATCTAAACAGAAGGGCAACGTGACCCACCCTTGGAACGGCATGGTCTGGGAGCTGATGACCGAGAGGGCCGATATGGCCATTGGAGATCTGACAATTTCTGCCGAGAGAGAAAAGGTGATCGACTTTACCAAGCCCTTCATGAACCTGGGAATCTCTATCATGATCAGAAAGACCCCTGGATCTCAGTTCGACATCACAGATATGTTCATCTTTATGGTGCCTCTGAGCAAGGAGATCTGGATGTGCATCGCCTTCGCTTACCTGGGCGTCTCTGTGGTGCTGTTCCTGGTTAGCAGATTCAGCCCCGCAGAGTGGCTGATCAACGACCCTATGGAAGATGATGGGAAGAAAAAGACCTGTGTGCGGAACGACTTCGGAATCTACAATAGCCTGTGGTTCGCCCTTGCCGCCTTCATGCAGCAGGGCTGTGAAATTTGTCCAAGAAGCGTGTCCGGCAGAATCGTTGGTTCCGTGTGGTGGTTCTTCACCCTGATCGTGATCAGCAGCTACACCGCCAACCTGGCCGCTTTTCTGACAACTAGCCGTATGATCTCTCCTATCGGCTCTGCCGAAGATCTGGCCGCTCAGACCGAAATCTCGTACGCCACAAGCAGAGAAGGCACCTCCCGAGATTTCTTCCGCGAGTCCAAAATAAGCACCTACGAGCGGATGTGGGCCTACATGAGCAGCAGACCCCACAACTTTCCAAAGAACAACGCCGAAGGTGTGAGACGGGTGCGGGACGAGAACGATAAGTTCGCCTTTCTGATGGAATCTACATCTATCGACTACCACAGCAACAGAGAGGATTGCACCACCACCAGAGTGGGCGACCTGCTGAACTCCCGGAGCTACGGCATCGGAATGCCCAAGGGCTTCTCTAAAAAGAACGTGGTGACACTGCAGCTACTGACCCTGGTTGAGAGCGCCTTTGTGGAAGAGCGGCGGAAATACTGGTGGGAAGAGGACAGCTCAAGAAGAGGCCTGTGCAAGGACAGACAGGACACCAACAAGAAGGGCGAGGACACAAGCAGCTCCCTGAGCATCTCTAAGATCGCCGGCATCTTTATCATCCTGCTGTGCGGCATGATGATGGGCATCATCATCAGCATCCTGGAACTGTTCTACAAGACAAAGATCGACGCCTTCAGAAAGCACATCACCTTCAGCGAAGAGTTCCAGAGCAACGCCCAACTGCTGATTAAGGGCAAGATGCCTCTGAAAGACGGCGAGGACGCCTCTGAAGATGTGCTCCCTGGCGACACCGCCGTGAAATGTACCACAACCACCCCATTCGCGCAGCTGATCACCCTGGACGGCTACAGCGACAACACACAGACCGAGGTGTGAGAATTC

*Lingula anatina* GluA-g16613 (codon-optimized for human)

GTCGACATGGAAACCCCTGAGATCACCCTGTGGCTGATCCTGTGCGCCTTCACCCGGCTGTCTATCAACGGCCAGCGGGCCGAGGCCTACGTGCCTGAGAACATACCTATCGGCGCTATCCTGGGAAAAGAGTCCAGACAGGTTGAAACAGCCGTGCGGTACAAGATCAAACAATTCAACGGAAAGAAGGGCAGCGATTACCTGGGCTTTAAGGTGGAAGCCAGCCTGCACTCTGATGTGAACGTGGACAATCCTAATGAGTTCATCGGAAGCATGTGCGACCAGATGAACAAGGGCATCTTCGCCTTCATCGGCAGCTCTCACCTGCCTGCCGCCGCCAGCGCCTTTGACTTCGCCGAGAAGTTCCACATGCCTTACTTCACGACTTCTAGAGGAGGCGTGACAAGCCTGAGAAAGCATTTTTACAGCCTGCATATGCTGCCCGACCACCTGGATGCCATCATCGACGTGATGCTCCACTACAAGTGGACCGACTACATCTACTACATCTTCGACGACGAGGATGGCTTCGGCAGACTGTCTTATATCTACAAGAGACTGGAGGAGCTGGGCCAGCAGATCCCCATTGCCGGCGCCAAACTGAACGACACCAACAACGCCTATGCCGAACTGAGAATGCTGGATAACCTCCCTCAGAGATTCGCCAACAAAAAGATCATCCTGGACATGAGCAGCCACAGTGTGCTGAAGAAAGTGCTGGAACAGATCAGCATCGTGGGCATGAACAAACACGGCTATCACTACATCCTGGGAACCCTTGCTATCGAAGAGCTGAATCTGACCCAGTTCCGGCACGGCGGCGTGAACATCACCGGCTTCAGCCTCCTGAACTACACCTACTACGAGCTGGACAAGTTCATCGAGGATTGGGCCAAGCCCCCCCCTGAAGGCACCGGTATGAAGACAATTGAGCTGACCAGCGCCCTGTTCGTGGACAGCATCTCTGTGATCACCAAGAGCCTGAACACCATGATGATTAACAAGAAAGACGTGTTTAGCAATATCTTCCGGGATGGCGCCCTGTACAACAACGGCACAAGAGGAATCAAGTGCTTCAGCAGATACCAGCCTACACCTTGGATTCACGGACCTGGCATCATTGAGGCTATTAAGACCCCTAGAGTGGCCGGCCTGAGCGGCGAGATCGTGTTCGATGCCTACGGCAAGCGGATCAACTACTCAATGGACGTGCTGGAAGTGTCTCTGGATTCAGGTCCTATCAAAATCGGAGATTGGTCCAAGGAAAACGGCTTCCTGACTAACAAGCTGGGCGAAGGCCTGGAGGACAAAAAGCCTAACGTGACAGCTACCAGAACAATCCAGATCATCACAACCGTGCTCGCCGCTCCTTATGTGATGATGAGAAAGCCTGAGCCTGGCCAGATCTTAACAGGCGTGGACAGATACGAGGGCTACTGCGTGGAGCTGGCCAAGCTGCTGAGCGAGGTGGTCCCATTCAACTACACCATCAAGATCGTGGAAGACGGACAGTACGGCACACTGGATTCTAATGGAAGATGGGACGGCATGGTGGGAGAACTGACCCGACAGACCGCCGACATGGCCATCGCCCCCCTGACCATCAAGAGCATGCGGGAAAGAGTGGTGGACTTCTCCAAGCCCTTCATGAGAACCGGAATCAGCGTGATGATCAAGAAGCCCGAGAAGCAGAAACCAGGCGTGTTCAGCTTCATGGACCCTTTGGATAGAGAGATCTGGATGTCTATTACATTTGCCTTCGTGGGCGTTTCGATCGTCATGTTTCTGGTCAGCAGAATTAGCCCCTACGAGTGGAACATCGTGAAGAACAATGGCGACGGCATGAAGCTGAAGAATGCCTTTACACTGAAAAACTCCATGTGGTTCTCTTTTGGCGCTCTCATGCAGCAAGGCACAGATGACTGTCCTAGCTCGGTGAGCGGCAGGCTGGTGGGCAGTGCCTGGTGGTTCTTCACACTGATCATCATCTCCAGCTACACCGCAAATCTGGCCGCCTTCCTGACGATCGAGTCCATGGTTCCACCCGTGAACAGCGCCGAAGAACTGGCCAATCAGGTGGACATCAAGTACGGTACAATGGAAAAAGGCAGCACCTACGATTTTTTCAAAAGCAGCAAGATGGAAACCTACAAGAAGATGTTCAAGTTCATGAGCAGCCAAGACCCTAAGGACGTGTTCGTGCGGGATAATGAAGAGGGAGTCAAGAAGGTGCAGAACAGCAAGGGCAAGTACGCCTACCTGGTGGAATCTACCACCAACGAGTACCACAACCAGCGCAAACCATGTGACACCATGAAGGTGGGCGCCAACCTGGACAGCAAGGGCTACGGAGTGGCCACCTATCAGGGTCACCCCCTGAGAGACGCCGTGAACCTGGCCATCCTGAAGATGCACGAGGACGGCACACTGACCAAGCTGAAGCAGAAGTGGTGGTACGACAAGGGAGAGTGCGGCAGCAGCAGTGACAGCAAAACCAACGAGCTGACCTTGAGCAACGTGGCTGGCATCTTCTACATCCTGATCGGCGGCCTCGCCCTGGCCATGCTGGTGAGCCTGTGTGAGTTCCTGTACAACTCCCGGCAAGATAGCATCATGTACAAGAAGTCCTTCAAGGACACCGCCCGGACACAGGCCAGACTCAGCATCAGCGGCCGGTGCGGCATGGGCGGAAAGGACAGAAGCAGCTTTGATAACAGCTCCGCTTACGGCGTGCCTTACGCCTACACCGCCCCTAACGGAATGATCACATTCAGAGGCTATGACGAAAAAACCGATACCGAGGTCTGAGAATTC

*Hofstenia miamia* GluA-98007595 (codon-optimized for human)

GTCGACATGGTGGGCCTGCTGAGCCTTATCTTTAGAATCTTCTTAGCATTCTGGCTGTTCACCAACGTGAACAGCTTGGGCAGCTTCAACTACACAGCCATCTTTTCTATCAGCGATAACGATTACGACCCAGACCAGACAGCCTTTGGCATGCGGCAGAACCTGACATCCCAAGAGGAAACCAGCAGCAATACCGGAGTGAGCTTTACCTTTCTCGCTAGTACACCTACAGTGGACTTCGAAAACAGCTTTGCCCTGTCTAAGCTGCTGTGCAAGCACCTGAAGGAAAAAGAGCTGGCCGCCCTGCTCAGCACCGCTGCTTCTCAGGAAGCCAACAGTATGATCGCCTCATATGCCAACGCCTTCAAGGTGCCTTATGTGTCCATCAGCCCATATGAGATCGACGTCGATCACGACTACTACGTGAACCTGAGGCCTAACCTGATCAAGCCTATCCTGGCTCTGCGAAGAAAGCTGGGATGGGAGAGACTGACGTACATCGTGGACAGCCAAGAGGGAATGAACCGGGTGCGGGATATCCTGGACAACCTTGAGGATAGTTCTATCGACTTCCTGGTCAGAAGCCTGAACACAAGCGTGAATGTGGAGTCTGTGCTGGCCGAAATCAGAGACGAGAGCGAGACCAAGATTATGATCGATTGCAGCATGAACATCACCATCGAAATCCTGGAAGCCATGTACCGCATGCAGATGCTGCTGAAGCAGTACCACATCGTCATCGTGAACGTGGCTGCCCCTAGCAACATCGATCCCAAGTACGGCAAGGGAGGCGTTAACATCACAGGCTTCAGCATGTCCCGGGACGTGTTCACCAGCCTGGTGGAGTTCCAGCTGCACTGGAAGACAACCATCGAGACACAGCCCAAGATAACACCATTCTACGAGTTCAGCTCCATGCTGGCCTATGACGGGTACGACCTGCTGTACAAGGCCTTCAAGCAGAGCCAAAAGGTGGCCGACAACCTGTACAGGCTGTCCCTGGAGCGGAACCTGAGCTGTGAAACCGCCGGCAAAGTGAACAACGAGATCAGACAGAAGGCCGACATGATCGTGAAGGTGATCAAGAACATGCGGATGAACGGCATCACCGGAAATATCCAGTTCGATAAGAACGGCAGAAGAACCAACTACAGCCTGAATCTGGAAATCCTGCCTGAGAAGCGGGGCACCGACCCTGTGTACGTGGGCACCTGGGATCACGATCTCGAAGATTTCGTGCTGGACGGCAACTTCCGCTTCAATCAGCGGAATCCCAACGAGATGAAAAGACATCTGATCATCACATCCCTGATCGAGCCTCCTTTCCTGATGAACGTGACCGCCCAGGACGGCACCATCCCTACCGGCAACGAGAGATTCGAGGGCTACTGTAAAGACCTCGCCGACAAGCTGTCAAAGGACCTGGGCTTCACCTACGAGATCGTTCTGGTGGAAGATGGCGAATACGGCGGCAAGGTGAACGGTTCTTGGAACGGCCTGATTGGCGAGGTGATCAGCGGAAAAGCTGATCTGGCCCTGGCCGGTCTGACCATTAACGCTGAGAGACAGAGAGTGGTCAGCTTCACAAAACCTTTCATGAATATTGGCATCTCTATCATGATTCTGAGACCAAAGAAGAAACTGCCCGGCCCCCTGAGCTTCCTGGACCCTTTCAGCAACCACATCTGGATCGGCATCATCATCGCTTATGTGTCCGTGAGCATCGGCCTGTTCATCGTGGCCAGATTCAGCCCTACCGAGTGGAGAATCAAGGACACAGGTCAGCCTCCATCCACATCTGGCACACACCAGATGTGCCACAACGACTTCTCTCTGTACAACAGTATGTGGTTCTCTCTGGGCGCCCTGATGCAACAAGGCACCGACATCGCCCCACGGGCCACAGGCTCCAGAATCGTGGGAGGCGTGTGGTGGTTTTTCACACTGATCATCATCAGCAGCTACACCGCCAATCTGGCCGCCTTCCTGACCGTTGAAACCATGGACACCCCTATTAAGAACGCCGATGACTTGCAGTCCCAGACCGATATCGCCTACGGCACCAGAAGAACCGGCTCTACCCGGACATTCTTCGAAAACAGCGAGATCGACGTGTACAAGAAAATGTGGATGTACATGAAGGACAAGGAAGACGTGTTTGTGAGCACCAATGACGAGGGCATCAAGAAGGTGAGAAACTCCAACGGAAAGTACGCCTACCTGATTGAGAGCGCCGCCAACGAGTACGTGCAGAGCCAGAAACCCTGCAACACCCTGATGGTGGGCCCCAATCTGGATTCCAAGGGCTACGGCATCGCCGCTAACCTGAATAGTACGTGGATCAAGAACATCACCCTGAAAATTCTGAAATACCAGGAGGACGGAGAACTGGAGAGACTGAAGACCACCTGGTGGGTGGACAAGGGCGAGTGCGGCGGCGCCTTTAGCTCTACAAAGACCGACTCTACAAACTCCCTGGGCCTGTCCAGCGTTGCCGGCATCTTCTACATTCTGATCAGCGGCCTGGCCCTGGCCATCATTAGCGCCATCCTGGAATACCTGCTCAAGAGCTGGCAGCACAGCATCAAAACCAAGTCTGGCGTGCGGAATAGCCTGCGGTACAACGCCGAGCTGAGCGTGATTGGAAGAGAACAGCAAGCCCCTCCTTCCTATACCAGCTACGTGAACTCCTCTCAGAGCACCGACTCTATGTTCAGCCATCCTATCCAGGGATTTAGCAGCCCCGCCGCCTCTCTGCACAAGAGAACCAATATCTACGAACACAATAGCCCTAACACCATCCACAGCACAGTTTACGACAGCCTGCCCAGCCCTTACGCAACAAAGCTGGTGCACAACAACAGCAAGAGCAGCCACACCGACGTGTGAGAATTC

*Hofstenia miamia* GluA-98014323 (codon-optimized for human)

GTCGACATGATGCTGGCCCTGATCTACCCTCTGTTCATGTTCCTCCAAAGCCCCACAGTGTCCCTCGAGAACTTCAAGATCTTCGGCCTGCTCGACAGACACCTGCTGGAGGAAGAGATCGTGTTCAACTACCTGAGCACACCAACCTACAACGAGCCTAAGCATTACATCGCTGTGGAGTTCCAGACCAACAAGGTGTTCGACCTTACCAAGATGCTGTGTGATCGATTTCACAACCAGGGCGTTGCCATCTTTACACCTGAGCTGCCTATAGTCGAAACCAGAGTGATCAACTCTTTTGCCCGGGCCTTCAGAGTCGGCCACGTGACACTGAGCCGGGCCCCTCTGCCCAACACCTTTCGGATCAGGCCTAGCCTGGCCAGACTGGTGGCTGAACTGACAATGAAATACTACATGTGGGACACCTTCGTGATCTTCTTCCAGTACGAGGAAGATGTGATCGAGCTGGAAGAGGAAATTAACATCAGAATCCCTTCCGGAATCTACGCCAATTACCGGAGATTCAACGTGAACAGCAACAGCGAGACAGTGGACAACCTGCTGATCCAATACCAGAAGATTGGAAAAGGCCGGTTCATCGTGGCCTGCGACCTGGAAACCACCGTCAGCATTATGAGCGCTGCTCAGAACCTGCAGATGCTGAGTAGAGACTTCCACTACATCCTGGCCGACATCGAGAGTCCTTGCAGCCAGCTGAATCTGACCAGCTCTGGCAGAATGAACATCACTTGTTTCCAGCTCCTTTCCAAAAGAAAGATCAACGACCTGTACACAGATGTGAGTAAGAGCCGCCTGGACATCAGCACCAAGGCCTTCCTCAACAGCAATGAGTTCAGCAAGCTGAAGATGGCCCTGGCCCACGACGCCTACAAGGTGATTAAATCTGCCTATGACAGATTCCTGCACGCCGCCCCTAACAATTACAAGGCCCCTAGCGACCCCCCCTGTCTGGGCAAAGAAATTAGCAAGATCCCGCGGTACCTGCTGTATAAGGAAATCGAGAATATCAGACTGGAGGGCCTGAGCGGCAACATCCAGTTCAACAAGTTTGGCCAGAGAGTGGACTTTAACGTGACAATCTCCGAGCGGCCATTCAGCAACGATAAGGATGTGAAGATCGGCACCTGGACAGACGACAAGGGCCTGTTGCTGACCAAGGCCGGGAGAAGCCTGCCAAGCATGAGCTCTGCCACCGTGCGGGTGGTGACAATCGAGAGCAAACCATTTACCAGCATCAAGAAGGGCAGAAGACCCGAGGAAGGCAATGACGCCTACGAAGGCTTCTGTATCGACCTGTTCAGCGAGATCGCCAAGAACCTGAACCTGAGCTACGAACTGCATCTGGTGGCCGATGGGAAATTCGGCGAGGAACTGAGCAACGGCACCTGGAACGGCATGATCGGGGAGCTGATGAGCGGCAAGGCTGATGTGGCCCTGGCCAGCCTGACCATCACATCTGAGAGACAGAAGGTGGTGGATTTCACAAAGCCATACATGGACGTGTCCGTATCTCTTATGGCTAAGATCCCTGAACACGAGAAGTCCAGCTTCGCGTTTCTGATCCCCCTGCACTACGAGATCTGGCTGTGCATCCTCTTCTGTTATATCAGCGTGTCTATCGTGTACTTCATCACCGCCAGAATCAGCATGATGGCCTGGCGGGACGGAGATGAGAGATACTACGACCAGAATGGATTTAAGTACACCATCTCTAATTGCTTCTTTCTGACCCTGGGTGCTGTGCTGCAGAGAGGCGAGGACATGTGCACCAAGGAATCACCTTTCAGCTCCAGACTGGTGGGCGGCGGCTGGTGGTTCTTCACCCTGATCATCATCTCTTCCTACACCGCCAACCTGGCCGCTTTCCAAACCGTGGATAGAATGAAGACCGAAATCAACAGCGCCGAGGATCTGGCTGCCCAGACGGAGGTGCTGTACGGCAGCATTGACGGCGGCTCCACCAAGAGCTTCTTCAAGAACAGTCAATCTGGCACATTCCAGAGACTGTACATGTTCATGAAGGACCGGAAGACATTCACCAAGAGCACACTGGATGGCGTGCAAAAAGTGCGGGACTCTGACGGAAAGTTCATCTTCCTGCTGGAATCCACCATGAACGAGTATTACAACGCCAGACCCCCCTGCAACACCATGCGGATTGGAGGCAACCTGAACCTGAAGAACTATGGAATCGCCACACCTCAGGGCAGCCAGCTGCTCGAAAACTTCACAGTGCAGATCCTGGAGCTGGGCGAGAGCGGATTTATCGCCAACACAAAGGAAAGCTGGTGGTACGAGGGCAGCGAGTGCGTGGAAAAGAACTCTAAAAAGGAAGTGGGCAAGGATACAAACGCCCTGGAACTGGACAGCGTCTTTGGCGTGTTCTACATCCTGTTCGCCGGCATGGCCCTGGGATTTTCCGTGTGCATCTTTGAGTTCCTGGTGTTCTGCTACAGACAGTCTCGGCAGTTCAAGGTAAGCTTCTTCACCGTCGTGCACGCCAATTTCGTGACCTGCTTCAAAAGCATGTTCGAGATGAACAGCATCGGCTCCCTGTCCCGTGTGTACAGCCCCATCAAGCAGACACAGCTGCCTATCGCCAACAACAAAATCCACAGACTGCTGAGCAGCCAGCAGAGCAGCTTCGAGAGCAATATCAGCGACTGCCCCGTGCTGCCTAGCAAACCTACAACCAACAACTTCTTGCCTGTTCAGCACAGGCCTAATTCTTTCCAACTGGCTGCCGTAAGAGTGTCCACCAAGAGCAGTCCTTCTAACCACACCACCGTGTGAGAATTC

*Hofstenia miamia* GluA-98019615 (codon-optimized for human)

GTCGACATGACACTCACCAGCCACTGGCGGTACCTGTTTTTCTTTACCCTGTTCACATTCCCTCAGCAAGAGCTGGCCTTTAACAAGTACAAGATTGCCGCTTTCTTTCCTGAGGATGGCAGCCTGGAAAGAAAGATCTTTCAGTTCGCCGCCGAGGAATACAAGGACATCGAGATCGTGTCTCATGAGTTCTCCACCAGCAATCTGTTCAACCTGAGCAGCACCCTGTGCAACCTGGGCAAGTCTGGCATCGTGTCGGGCTTCAGCGGCAGCAACCAGTTCGCCACAACCTCTGTTATTCAGAGCTTCTTTGACGCCTTCGACCTGGCTCACGTGACATACCAGACTGAGCACAGCAAGATCAATCAGGCCTTCGAGCTGACCCCTAGATTCGGCCCTCTGATCTACAGCCTGCTGACCAAGATGGAAGTCAAGAAGGTGTACGTGATCTTCGACAAGCCCCACGCCATGAACAGAATACCTGAGCTGGTGAATATGGGACCCTACGTGATCAAGAACGAAGTGCTGAGAATCAACGATAACACCACCCTGAAGCGGAGAATGCAGGAAATCCAGAACGGTTCTATCCGGAACATCATTCTGATGCTGAGCTACCCCCTGACAGAGCTGTTTTTCCAAAACGCCCTGAAGATGCAGATGCTGAGCCCGGACTACCGGTACATTATCGCCGACTTCAGAACACCTTGTAGAAAACTTAAATTCAAGATCACCGGCAAAATCTCCGTGACATGCCTGCAACTGGTCAACGAGCAGGCCCCTCTGGTGCAGGCCTTCATGAAAAGATGGCTGCAGAGCGTGCAATACAAAGAATACACAAAGCTGCACCCCAAAACCCAGTTCTACAGACTGCCACCTAACGAGATCAAGCTGGAAATGGCTCTGGCTTATGACAGCTTTCAGGTGACCAAGGCCGCTATCAAGAACGTGAGCCCCTACAGCAAGATCGGCTGCGAGAACCTGGATAGAATCTGCATGTCTAGAAGCATCAAGATCAACTGCTTCGGCATCAAGCTGAAGCAGCAGCTGAGCAGAATCAAGATCAACGGCCTGACAGGAAACATCGAGTTCGACGGCAATGGACTGCGGAAAAATTTTAAGATCGAGGTGTGGGACGTGGGCTTCATGGAATACCACAGCAAAGTGGGCGTGTGGACCGATGGCGAGGGCTTTACTGAGATCACAAGCTCTAAATTCCCCGCCTCTTTCTCTGATATCTCCGACAGGGTTATGAGAGTGACCACCGTGCAGAACAGCCCTTTCACCATGATCAAGGAGGAATACAAGAACTGGAACAGCAGCGAGAAGGGCGATCTGCCCAATGACGCCTTTGAGGGCTACTGCGTGGACCTGATTAAGGAAGTGGCCAAGGAACTGAACGTGTCTTACAAGCTGACCCTGGTGAAGGACGGCAAGTTCGGAGCCGAGACAATCACCGGAAAGTGGAACGGAATGATCGGCGAGCTCCTCGAAGGCGAAGCTGATGTGGCCCTGGCCCCTCTGACCATCAACCTGAGCCGGCAGCAGGTGGTGGATTTCACCAAGCCTTTCATGACCCTGGGCATCAGCATCATGATGCGGAAGCCAATCAATAAATCTGATGTGTTCAGCTTCCTGTACCCTCTGCACAGAGATATATGGATGTGCTGCCTGTTCGCCTACATGAGCGTGTCTGTCGTGCTGTTCCTGGTTTCCAGATTTAGCAGCACCGAGTGGCACTCGATCAGCGTGGGAAATGAGAACGAACTGTACTTCGAAAAGGAAAACGAGTTCGGCATCATCAACTGTCTGTGGTTCAGCCTGGGCAGCTTCATGCAGCAAGGAGGAGATGTGTGTCCTACCAGCATGCCTGGCAGAATCGTGGCCGCCAGCTGGTGGTTCTTCACCATGATCATCATCTCTAGCTACACCGCTAATCTGGCTGCCTTCCTGACCGTGGACAGAATGAAGACACCCATCACAGGCGCTGACGACCTGTCTAGCCAGACAGCCATCGAGTATGGCGTGCTGGACCAGTCTAGCAGCCAGGCCTTTTTCGAGAAGTCCACAATGCGAACATACAGAAGAATGTGGAGCTTCATGAAGGAAAAAGGCAACTTCGAGAAGACCACAGCCGACGGCGTGAAACGCGTGATCGAGAGCGACAACTACGCCTTCCTGATCGAATCCAGCATGCTGGAGTATTACAACCAAAGAAAGCCATGTACCACCGTGCAGGCAGGCTACAACCTTAACGTGATCAACTATGGCCTGGCCACCCCTAAGAATAGCCCTTACCTGGAAAACCTGACGATCGTGGTGCTGCAGCTGAAGTCCAACTTCCTGGCCGTGAAGAAACACGAGTGGTGGGTCGAGAAGGGGGATTGCAACGAGGCCCCAATCAAGAAAGAGGACACCGGCGCCAACGCCCTGAAGATCGACTCCGTGGAAGGCATCTTCTACTTGATCAGCGGCGCCATCTTTTCTGCCGTGTTCATCACCGCCTTTGAGTGGGTTTTCGTGTCAGTACAGGACTCCCTGCGGTTCAAGGTGCCAGTGAAGAAAGCCCTTAAATCTAACTTCAACATGGCCGTGACAAGCGGATACGACCCTCCTAACCTGAGCCCTCGGCAGAACGAGGAAAAGAAGTACTTCGGCAGCCAGCGGAAGAGTTACCATAATGAGGCCAAGAACCCCACCGACGTGTCTTATTTTTCTCCATCTCACACCGACGTGTGAGAATTC
