# Supplementary material for: Tunable AMPA receptor function via recurrent evolution of heterotetramers
Source: J Gen Physiol. 2026 Aug 27;158(6):e202614039. doi: 10.1085/jgp.202614039 (PMC13520880; doi:10.1085/jgp.202614039)
Supplement: Supplementary file 4 [file jgp_202614039_tables4.docx]

**Table S4. Synthetic TARP genes used for expression in HEK293 cells**

*Saccoglossus kowalevskii* TARP-XP_006816113.1 (codon-optimized for human)

GTCGACATGCCCAGCTGCAGCGAGAGAATGCTGGTGATCAGCACCACAGCCGGCTACGTGGCCTGCCTGTGTCTGCTGAGCACAGGCGTGGCCACCAGCCACTGGCTGTATAGCGTGGAACGGTCTGAGTACAGCACCTTCAACGGCACCACCATGGTGGTGGAAGTCACAATGCATAGCGGACTGTGGCGGCTGTGCCCCCTGAGCGGCGAGAACGTGGGCGACTGTAGAATCATTGAATACTTCACCCCTAATCCTGAAATCACCAGCGACACCACAACAGCTGTGGCCAGACTGATCAGATCTTCTGCCGCCTTCCCCTGTGTGTCCCTGCTGCTGATGATCGTGGGTACCGCCCTGGGCGTGGCCTCTCAGAAGCGGCCAGAGCAAAAGCACCTGGTCTTTGTTGCTGGCATCGTGTTCGTGACAAGCGGCCTGTCTCTGTTCGTGGGCATCATCATCTACATCAGCAGCATCAACGACGCCCTGTCCGATCAGCCTACCTCCAACGGCCACAGATACACCTACTACTACGGATATTCCTTTGTGCTGGTGACCGCCAGCTTCGGCCTTAGCGAGGTGTGCGGCGTCATGTGCATGTACCTGTACATCCGGAGATACATGAACCAGGAGGAAAACGAGAGCAGAAGGAAAAGCCGGCTCAGACGCTACAACGTGGGAAACATTGAGGAAAACGAGGTGGCTAATAGACTGGTGTACAGATGCCCTGAGGTGATGATCCCTGCCTGAGAATTC

*Saccoglossus kowalevskii* TARP-XP_006822518.1 (codon-optimized for human)

GTCGACATGGCCAGAAGCTGCAACAGACGCAGACTGCTGATGCTGCTGACATGCCTGAGCAAAAGCAGTTCCGCCATCATGCTGGCCATCGCCCTGAGCACAGATTTCTGGATCAATACCAAGGACGTGTCCTACAAGGAAAAGCAACTGGCTGGCGAGGAAGGCTCTGAGAACGAGACAGCCATCCACGAGCACATCGGCCTGTGGCGGATGTGCACAAACCTGGGCATGGGCTACTTCGTGTGCACCAGAATCGACTATTTCCCTGAGGGCCTGGAAAGCGACTCTTCTACCGATAGCGAGGGCAAGATCCTGAGAGTGGTGCGGGCCAGCTCTGCTTTCCCCATCCTGGCCCTGATTGTGCAGACCATCGCCACCATCGTGTGTTTTGTGGGCCACTACAGAAAGCACAAGGTGCACCTGATCCTGATCGCTGGAGTGCTGAGCGTGCTGGCCGGCCTCTGTAGCATCGTCGGCCTGTTCATCTACATCAGCTCTGTGTCCGAAGAGTACGCCTCTAAGGCCCGTCCAAGAAGCGCTATTAGCGACCCCACCTTCTACTACCACTACGGCTGGTCCTTCTGGCTGGCTGTGTCTAGCTTCACCCTGACCGAGGCCGCCGGAGTTCTGTCTGTGTACCTGTACGTGAGCAGACATAGACACGTGATCAAGAAACACGTGGATAGCCGGAAGCACGTGGACGCCTTTCCTAGACTGAGGTCCGATAGCGGCAGCCAGGACATGTACCGGCTGACCAGAAACCAGCACGACTACTATAGCGCCCCTCACCTGCAGGCCAGCGATTACAGCACAGACAGCTCCCGGGACATCGGAATGAGCCTGCTGAACAGCACCGAAAGACACCCCAGCCGGAAGCTGTACATGCCTAGACGGGAAAGAAGACTTCCTAGACAGCATTCTTTCGAGACCGTGCGGAAAACCACACCTGTGTGAGAATTC

*Lingula anatina* TARP-g12410 (codon-optimized for human)

GTCGACATGAAATGCAACGAGAGGGCCATGACCCTGCTCGCTCTCGTGTTCGGCACCGTGGCCACAACCGGCCTGTTCCTGGCCCTGACCACAACCTACTGGCTGTATACCACCGAGAGACCTGAACAGCTGAGCAGCTCCGCTGCCAAGGAAAAGAAAAACACAACACGGGAAGAGGGCAATGTGACATACGCCCATCTGGGCCTGTGGCGGGTGTGTCTGTACAGCAAGCAGGACCCTGATAGACCCAGCGAGTGCGCCTCTATCGACTACCTGTCTAAGGACCCTCCTCAGCTGCTGGGACCTGAAAGAAAAAAGCTGCCTAGCGAGATCACCCTGGCCCTGGCTAAAGGCACAGGCATCGCCGCCCCTCTGTGCACCAGCGGCCTGGTGCTGCTGCTGATCGGAATCGTGATATCTGCCATCGGCATCGTGAAGCGGGACTTCAAGATCCTGATCGGCAGCGTTTTTCACATCCTGGCCGGCCTTTGTCTGGCCTCCGCCGTGATCATCTACATCAGCGCTATCAACGACGAGGTGGCTCACAGACCACAGCCTCAACCAGATGAGAACCCCGAGAATTTCCAGTTCATCTACACCTACGGCTGGTCCTTCTACTTCGCCGGATTTTCTTTTATCTTCACAGAACTGGATGCCATTATCAGCATCCTGCTGTATCTGAAGAGATACAAGCACCTGGACGACAAGATCCAGATGGTCCCCGGCCTGTACAGAAAGATTGAGAAGCACAACAAGGGGGGACACACCAACCAGAGCTGCGACACCTCTGTGGAAGCCAACACGAGCACCAGAGGCAGCCCCACCCTGATCCTGTGAGAATTC

*Lingula anatina* TARP-g21034 (codon-optimized for human)

GTCGACATGGCCTGGTGCGGCAGAAAGAGATTCCTGATGGTGCTGACAAGCCTGTGCGCCTCCACCGCCTTCGCCTTCCTGAGCATGGGCGTGGCCACCGACTACTGGATCTACATGACCGAGATCGTGACCGGCTGCAATCAGACCGAGGTGACCAAGAGCACCCACGCCGGCCTGTGGAAGAAGTGCTACAGCCAAGAGTGCATGAAGGTGAAGCTGAACTGCAGCTACATCAAGTACTTCAGCCTGGAGGAGAAGGACGACAGCCAAAGAACCGAGGCCATCCTGTACAACATCAAGACAAGCACCATGATGCCCCTGATCAGCCTGCTCCTGCAGATGACCGGCGGCATCGTGTGCTTCGCCTCCCACTGCTACGACAAGAAGCTGTACCTGAGCTTCGTGGCCGGCGTGCTGTTCGTGCTGGCCGGCCTGAGCACCCTGATCGGCCTGATCTTCTACATCGGCGCCATCGCCTCCGAGGCCGCCCACGTGAGACCCCCCGGCATCGGCGAGAAGCCCGTGTTCACCTACAGATACGGCAGCAGCTTCACCATGGTGGCCACAAGCTTCGCCATCATGGAGCTGAGCGGCGTGCTGAGCGTGTACCTGTTCATCTTCAGATACAAGAGCGAGAACAGAAAGAAGAGAGAGAGACTGAGAGCCATCAACGACAGCGTGCAGCCCACCGAGAACCACGTGGGCGGGGGCGCCTCCCACGTGATCAGAAACAGCAGACGGAGAAGAAGCAGAAGCAGAAGCCACGACAGAGAGCACAGCAGAGAGAACAGCCCTAGCCACAGCGAGAGAACCTACTTCACCTACGCCGCCGGCATGAGCAGAGAGACAAGCAACTACACCCTGAGCAGAGACTTCCCCGGGCAGAACCACGCCGACATGTTCCTGCTGAAGCCCCTGAACCCCAACAACCACCACAGCGACAGCAGCCACAGAATGACCCCCGTGTGAGAATTC

*Hofstenia miamia* TARP-98036255 (codon-optimized for human)

GTCGACATGGGAGAAGATGAGAAGGTGCGGCAACTGAAAATCAGACTGTTCCTGATCATGACCTCCTTTTGTAGCATCCTGGCTCTGCTGACCATCCTGCTGAGCTTCTTCAGCAGCTACTGGCTGCTGACAGCTGAAACCTGGAAGGGCCCTTCTGAGCGGAACGAATCTACAGTCAAGAAAGATGTGCACAGCGGCCTGTGGGAGTTCTGTTACTACGACAAGAAGGACCTGATGAACAACGAGTGGAAGGACACCATGAACAGCGACGGCTGCGTGCCAATCAGCGATAGCAAAAGCGAGAGCGACGACGAGAGACCTACAAGCACAAGAGGCGCCCTCAAAAAGATCACCCACTACAGCGGCATCCTGATGAGGATCTGCCTGGCCGTGCTGTCTACAGCTTTCATCATCAACGTGGCCGGCCATGTGCTGAAGACCAAGATCCACATCTGTTTTTTCGTCTCCGGCGTGCTTTTTGTGTTCAGTGGACTGTTGCTGATTCTGACCCTGGTGATCTACGCCTCTAAGACCAACGAGATTGCCGAGAGCTGGCCCACCGTGGTGGAAAGCGAAGATAAGACCCCTGGCAAGATGAAGTACCGGTATGGCTGGTCCTTCTTCTGCTGCGGCCTGTGCCTGATCGCCAGCGAGTGGGCCGGAGTTTTCGCCATGTACTTCGTGCTGGCTAGAGCCAACGACATCTTCATCAGCCAGCTGAACAAGGACTCTAAAGAGGAAAAGAACAATGACGGCCTGCTGTACAACTGCGACTACAGCTTTAGCGATGCCTTCTCTAGACAGATGATCACAAATATGAGAAGCATGACCAACGGCAATAACGCCAGACTGAATGCCAGCTCCCAGATGACAACCCTGTGAGAATTC
