# Supplementary material for: Tunable AMPA receptor function via recurrent evolution of heterotetramers
Source: J Gen Physiol. 2026 Aug 27;158(6):e202614039. doi: 10.1085/jgp.202614039 (PMC13520880; doi:10.1085/jgp.202614039)
Supplement: Supplementary file 5 [file jgp_202614039_tables5.docx]

**Table S5. Summary of outside-out patches**

| Construct (amount of DNA transfected, ng) | Ratio | No. patches | Current detected? |
| --- | --- | --- | --- |
| *Saccoglossus kowalevskii* AMPARs | | | |
| *Sac*-GluA (600) |  | 10 | x |
| *Sac*-GluA (600), *Sac*-TARP-XP_006816113.1 (200) | 3:1 | 9 | x |
| *Sac*-GluA (600), *Sac*-TARP-XP_006822518.1 (200) | 3:1 | 30 | x |
| *Sac*-GluA (400), *Sac*-TARP-XP_006822518.1 (400) | 1:1 | 2 | x |
| *Sac*-GluA (300), *Sac*-TARP-XP_006822518.1 (500) | 1:1.7 | 1 | x |
| *Lingula anatina* AMPARs | | | |
| *Lin*-g8707 (400), *Lin*-g16613 (400) | 1:1 | 10 | x |
| *Lin*-g8707 (500), *Lin*-g16613 (500), *Lin*-TARP-g21034 (200) | 5:5:2 | 10 | x |
| *Lin*-g8707 (500), *Lin*-g16613 (500), *Lin*-TARP-g12410 (200) | 5:5:2 | 9 | x |
| *Hofstenia miamia* AMPARs | | | |
| *Hof*-98007595 (400), *Hof*-98014323 (400) | 1:1 | 10 | x |
| *Hof*-98007595 (500), *Hof*-98014323 (500), *Hof*-TARP-98036255 (200) | 5:5:2 | 9 | x |
| *Hof*-98007595 (400), *Hof*-98019615 (400) | 1:1 | 10 | x |
| *Hof*-98007595 (500), *Hof*-98019615 (500), *Hof*-TARP-98036255 (200) | 5:5:2 | 6 | 3 patches |
